# Epigenetic signatures mark early peripheral human B lineage bifurcation and differential transcriptional profiles in mature populations

**DOI:** 10.1038/s41467-026-75364-3

**Published:** 2026-07-14

**Authors:** Chiara Dionisi, Audrey Kelly, Michael J. Pitcher, Sherine H. Kottoor, Alana Dalton, Lucia Montorsi, Pawan Dhami, Michelle Kleeman, Richard J. Ellis, Cynthia Bishop, Jahangir Sufi, Deena L. Gibbons, Paul Lavender, Jo Spencer

**Affiliations:** 1 School of Immunology and Microbial Sciences, King’s College London, London, UK; 2 Genomics Research Platform and Single Cell Laboratory at Guy’s and St Thomas’ NHS Foundation Trust, London, UK; 3 Advanced Cytometry Platform, Guy’s and St Thomas’ NHS Foundation Trust, London, UK

**Keywords:** Humoral immunity, Systems analysis, B cells

## Abstract

During human B cell maturation, immature transitional (T1) cells transit from bone marrow into the blood. At the subsequent immature T2 stage, a separation into IgM^hi^ (T2M^hi^) and IgM^lo^ (T2M^lo^) developmental trajectories has been proposed. Here, we isolate T1, T2M^hi^ and T2M^lo^ cells from human adult and cord blood for bulk and single-cell ATAC-seq, CUT&RUN, RNA-seq and CITE-seq to profile their transcriptomic and epigenetic differences. We identify accessible chromatin domains discriminating between T2M^hi^ and T2M^lo^ cells in peripheral B cell development, with signatures persisting during the differentiation of T2M^lo^ to naïve B cells. Similarly, memory and marginal zone B cells retain epigenetic hallmarks of their T2M^lo^ and T2M^hi^ precursors, coupled with transcriptional diversity. Imaging mass cytometry with RNAscope of spleen, appendix and tonsil further validates the spatial relationships of expressed genes. Our study thus provides insights into B lineage T2 bifurcation and describes epigenetic signatures of B cell developmental pathways.

## Abstract (web summary)

Developing transitional T2 B cells express differing levels of surface IgM. Here, the authors analyze epigenome and transcriptome dynamics in IgM^hi^ and IgM^lo^ T2 cells and find differences propagating from T2 cells to naïve and memory cells or to marginal zone B cells, demonstrating early lineage bifurcation to shape B cell maturation.

## Introduction

B cells give rise to plasma cells that secrete protective antibodies in response to pathogens and vaccines, and thus they are key contributors to adaptive immunity^1^. They circulate between blood and lymphoid tissues, where they may become activated by direct interaction of their specific surface receptor with native antigens^2^.

An early indication that human B cells have functional subsets that selectively respond to different antigen types was the observed association between splenectomy and loss of antibody-mediated protection against bacterial pathogens, including those that can cause pneumonia and sepsis^3,4^. The spleen is particularly enriched in marginal zone B cells (MZB) that respond to T-independent antigens that have repeating subunit structures and are largely responsible for the immune response to the carbohydrate pneumococcal vaccine Pneumovax. Their loss is considered to be a major contributor to the immunological impact of splenectomy^3,5–8^. Classical memory B cells, instead, are derived from recirculating naïve B cells following cognate interaction with T cells^9–11^. Although MZB have been described as memory B cells, they resemble closely, and with which they share a microanatomical splenic location^12–14^, they differ in many respects, including their transcriptome, association with innate signaling and functional responses^6,15–18^. Despite their differences, whether MZB represent the mature B cell end point of a distinct developmental B cell pathway or whether they are a memory B cell subtype originally derived from naïve B cell precursors has been controversial^5,18^.

Supporting the concept that MZB and memory B cells are fundamentally different, we have previously presented evidence that they are derived from different immature precursors in humans^17^. B cells exit the bone marrow as immature transitional T1 cells that mature to T2 cells with a wide range in levels of surface IgM expression^19^. T2 cells with higher levels of surface IgM (T2M^hi^) express the gut-homing integrin α4β7^17^. A proposed subsequent differentiation stage of these T2M^hi^ cells in the gut-associated lymphoid tissue (GALT) leads to MZB cells that have somatically mutated immunoglobulin heavy chain variable region genes, and receptors with specificity for gut bacteria consistent with involvement of GALT germinal centers (GC) in their maturation^6,14,20,21^. In contrast to T2M^hi^ cells, the fate of cells with lower surface IgM (T2M^lo^) has not been studied previously.

Whilst studies in mice have made an outstanding contribution to the elucidation of mechanisms of B cell development and function, B cells in mice and humans are markedly different. Mice have B1 and B2 lineages that have no direct equivalent in humans, where the B1 population appears to be small and restricted to fetal life, adipose tissue, and the pleura and blood of individuals with pleural infections^22–24^. Also, whilst mice have MZB in the spleen, these are receptor-selected naïve cells that are different in their derivation and microanatomical location compared to human MZB^25–28^. In mice, MZB tend to be static, whereas human MZB circulate in blood, where they are recognizable by their CD27^+^IgM^+^IgD^+^CD1c^+^ cell surface phenotype^3,29,30^.

Lineage commitment within the immune system is associated with orchestrated interaction between chromatin remodeling and differential gene transcription^31^. Here, by combining multimodal epigenetic and gene expression data, we identify that T2M^hi^ and T2M^lo^ immature B cells have different epigenetic and gene expression features that they share with innate-like MZB and naïve B cells, respectively. On cognate interaction in lymphoid tissues, naïve B cells may develop into memory cells that retain the major epigenetic and transcriptional signatures of the early T2M^lo^ precursors. The signatures of early human developmental bifurcation we observe will inform future studies of B cell function and should be considered when working to understand perturbations of B cell populations in disease.

## Results

### T2M^hi^ and T2M^lo^ B cells have distinct epigenetic landscapes

**Fig. 1:**
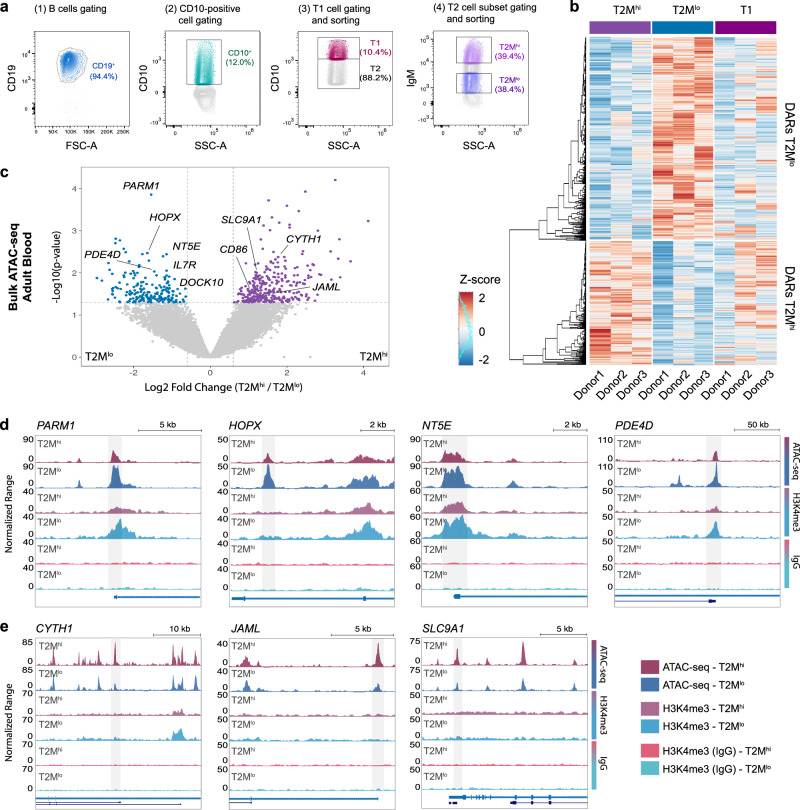
Bulk ATAC-seq analysis highlights epigenetic differences among transitional B cells in adult human blood. **a** FACS strategy for transitional B cell populations from MACS-sorted CD19^+^ B cells from human adult blood (*n* = 3 for bulk ATAC-seq; *n* = 2 for CUT&RUN). Cells were sorted according to their surface expression of CD19, CD10 and IgM. At the beginning of sorting, gates were set to capture the cells with the top 20% of CD10 expression within CD10 expressing cells. These were designated T1. Then gates were set to capture the top 35% and bottom 35% of IgM expression in the remaining CD10^+^ cells. These were designated T2M^hi^ and T2M^lo^, respectively. Percentages of cells at the end of sorting from a representative biological replicate are indicated in the plots. **b** Hierarchical clustering of statistically significant DARs between T2M^hi^ and T2M^lo^ cells (p-val <0.05 and abs(log_2_FC) > 0.6). Statistical analysis was performed using the DESeq2 method with Benjamini-Hochberg procedure for multiple testing. Chromatin accessibility for identified DARs was assessed in T2M^hi^, T2M^lo^ and T1 cells. **c** Volcano plot of DARs in the comparison of T2M^hi^ (violet) and T2M^lo^ (blue) cells. Statistical analysis was performed using the DESeq2 method with Benjamini-Hochberg for multiple testing. Colored dots represent statistically significant peaks with at least 1.5-fold increase in chromatin accessibility in each population (p-val <0.05 and abs(log_2_FC) > 0.6). Top DARs located at the TSS of coding genes are highlighted in the plot. **d**,**e** Bulk ATAC-seq (top) and H3K4me3 (middle) profiles of selected top DARs for T2M^lo^ (**d**) and T2M^hi^ (**e**) cells from adult PBMCs. IgG controls have been included for H3K4me3 CUT&RUN experiments (*n* = 2) in T2M^lo^ (**d**, bottom) and T2M^hi^ (**e**, bottom). FACS fluorescent-activated cell sorting, T1 transitional 1, T2M^hi^ transitional IgM^high^ T2 cell, T2M^lo^ transitional IgM^low^ T2 cell, DAR differentially accessible region, TSS transcriptional start site, PBMCs peripheral blood mononuclear cell.

To determine whether subsets of B cells at the T2 developmental stage discriminated by their relative surface IgM expression differed from each other and the more immature T1 population in their epigenetic profile, we sorted B cells by fluorescence-activated cell sorting (FACS) for analysis by assay for transposase-accessible chromatin with high-throughput sequencing (ATAC-seq)^32^ (Fig. 1a and Supplementary Fig. 1a, b and Supplementary Table 1). CD10 expression on human B cells is a continuum from high to low expression and it is important to acknowledge subjectivity in determining the precise subset boundaries. T1 cells were identified by their high expression of CD10. T2M^hi^ and T2M^lo^ cells were sorted based on their IgM expression, having ensured that these populations were equivalent in their maturity, as reflected in their equivalent CD10 expression (Supplementary Fig. 1c).

The efficiency of library generation was consistent across donors (Supplementary Fig. 1d). We observed that T1, T2M^hi^ and T2M^lo^ cells were epigenetically different (Supplementary Data 1). Epigenetic traits discriminating between T2M^hi^ and T2M^lo^ cells were consistent between donors and did not resemble DNA accessibility in the T1 population, demonstrating that chromatin remodeling at the T2 stage was not solely related to cell maturation, but was consistent with the emergence of divergent differentiation profiles in T2 cells currently defined by their level of surface IgM expression (Fig. 1b).

Among identified differentially accessible regions (DAR) in T2 cells (Supplementary Fig. 1e), we focused on statistically significant DARs localized at the transcriptional start sites (TSS) and adjacent promoter regions of protein-coding genes, to have greater certainty that they influenced the most proximal genes and their transcription (Fig. 1c). Again, identified regions were highly specific for each T2 cell subset and minimally shared with T1 precursors (Supplementary Fig. 1f). Differential accessibility was observed at genes including *PARM1, HOPX, NT5E*, a shorter isoform of the phosphodiesterase *PDE4D, IL7R* and *DOCK10* in T2M^lo^ cells (Fig. 1c, d and Supplementary Data 1).

The same *loci* were enriched with the H3K4me3 post-translational histone modification, as detected by Cleavage Under Targets & Release Using Nuclease (CUT&RUN) on the same B cell populations (Supplementary Fig. 1a, b, g, h and Supplementary Data 2), further supporting that these genes were in a chromatin conformation permissive for expression (Fig. 1d). DARs at TSSs for T2M^hi^ cells occurred at *loci* encoding *CYTH1, JAML* and *SLC9A1*(Fig. 1c, e and Supplementary Data 1). However, these were not decorated with H3K4me3, suggesting a different temporal deposition of post-translational histone modifications in comparison to DARs identified for T2M^lo^ cells (Fig. 1e).

**Fig. 2:**
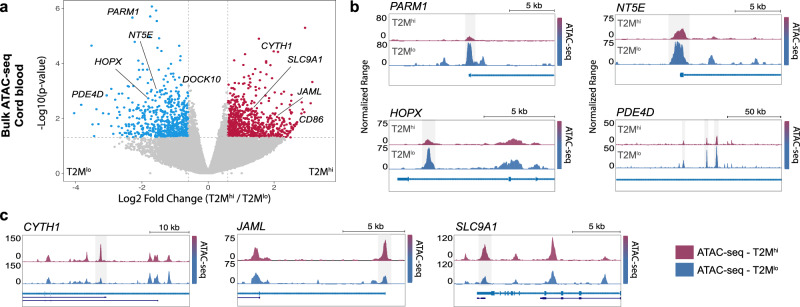
Epigenetic differences in transitional B cells are predetermined and detected in cord blood. **a** Volcano plot of DARs in the comparison of T2M^hi^ (red) and T2M^lo^ (blue) cells from human cord blood. Statistical analysis was performed using the DESeq2 method with Benjamini-Hochberg for multiple testing. Colored dots represent regions with a statistically significant differential accessibility (p-val <0.05 and abs(log_2_FC) > 0.6). **b**,**c** Bulk ATAC-seq profile of selected top DARs for T2M^lo^ (**b**) and T2M^hi^ (**c**) cells from cord blood. T2M^hi^ transitional IgM^high^ T2 cell, T2M^lo^ transitional IgM^low^ T2 cell, DAR differentially accessible region.

To test whether differences in chromatin landscape in T2M^hi^ and T2M^lo^ cells could be observed even before birth, we performed an ATAC-seq analysis on sorted T2M^hi^ and T2M^lo^ cells from human umbilical cord blood derived from the fetus, which is enriched in transitional cells and is antigen naïve^33^ (Supplementary Fig. 2a, b). Genomic distribution of regions with differential accessibility was consistent between donors and with previous observations in adults (Supplementary Fig. 2c, d). Cord blood T2M^hi^ and T2M^lo^ cells already shared DARs with their counterparts in adult blood, including DARs at *PARM1, HOPX, NT5E, PDE4D* in T2M^lo^ and *CYTH1, JAML* and *SLC9A1* in T2M^hi^ cells (Fig. 2a–c and Supplementary Data 3).

Thus, analysis of sorted B cell populations from adult and fetal blood identified epigenetic differences in immature B cells related to the level of surface IgM expression, independent of antigen exposure.

### Chromatin accessibility and gene transcription are not consistently coupled in early B cells

**Fig. 3:**
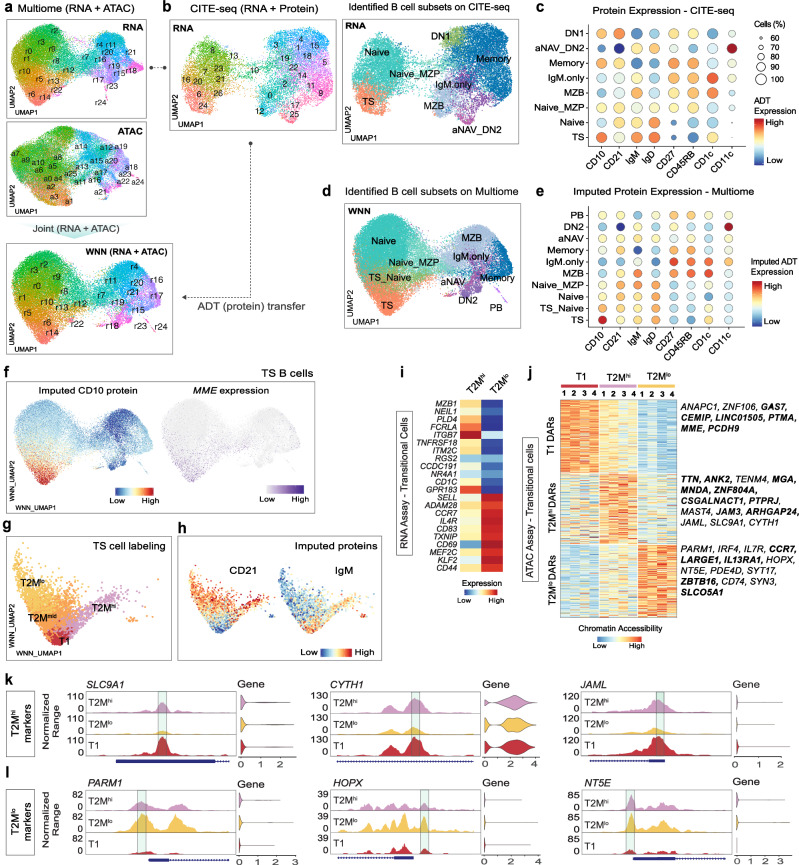
Multiome analysis allows the simultaneous investigation of the epigenetic and transcriptomic profiles of human B cells. **a** UMAP of nuclei from sorted B cells (*n* = 4 donors; nuclei *n* = 36905) from 10x Multiome based on snRNA-seq (top) or snATAC-seq (middle) profiling alone or in a joint (WNN, bottom) UMAP space. Nuclei in RNA and WNN UMAP plots are color-coded based on RNA clusters. Nuclei in ATAC UMAP are colored based on ATAC clusters. **b** UMAP of B cells from CITE-seq dataset based on scRNA-seq profiling (*n* = 3 donors; cell *n* = 17717). Cells are color-coded based on RNA clusters (left) or labeled B cell subsets (right). **c** Dot plot of z-score normalized mean expression of cell surface protein markers in CITE-seq dataset for labeled B cells. Dot size represents the percentage of cells expressing the protein, the color code represents the average protein expression per subset. **d** WNN UMAP of cell nuclei from Multiome colored by assigned B cell identities. **e** Dot plot of z-score normalized mean expression of imputed cell surface protein markers in Multiome dataset for labeled B cells. The color code represents the average imputed protein expression per subset. **f** Imputed ADT-CD10 protein (left) and nuclear *MME* gene (right) scaled expression from Multiome overlaid on WNN UMAP. **g** WNN UMAP of labeled transitional B cell subpopulations from Multiome. **h** Imputed ADT-CD21 and ADT-IgM protein expression on transitional cells overlaid on WNN UMAP. **i** Pseudo-bulk heatmap of z-score normalized mean expression of transitional B cell marker genes in T2M^hi^ and T2M^lo^ subsets. **j** Pseudo-bulk heatmap of z-score normalized chromatin accessibility of top 150 DARs for TS cell subsets, split between biological replicates (1–4). Genes associated with top DARs at TSSs are listed on the right-end side. Genes with association between higher chromatin accessibility and increased transcription are highlighted in bold. **k**,**l** Chromatin accessibility profile of T2M^hi^ (**k**) and T2M^lo^ (**l**) DARs common to bulk and single-nucleus ATAC-seq, and violin plots for gene expression. UMAP Uniform Manifold Approximation and Projection, WNN weighted-nearest neighbor, TS transitional, T1 transitional 1, T2M^hi^ transitional IgM^high^ T2 cell, T2M^lo^ transitional IgM^low^ T2 cell, MZP marginal zone precursor, MZB marginal zone B cell, aNAV activated-naïve cell, DN1 double negative 1, DN2 double negative 2 cell, IgM.only IgM-only, PB plasmablast, DAR differentially accessible region, TSS transcriptional start site.

To further characterize the B cell epigenome and to explore potential links between chromatin state and gene expression in distinct populations, we used the 10x Multiome method for the generation of paired single-nucleus (sn)RNA-seq and snATAC-seq data (Fig. 3a) on sorted CD19^+^ B cells from human adult healthy blood (*n* = 4). Quality control (QC), integration, clustering and analysis of data were performed using the Signac and Seurat packages for analysis of genomic single-cell assays^34–36^ (Supplementary Fig. 3a–c). B cells showed heterogenous transcriptomic and epigenetic profiles, as indicated by their relative positions within the uniform manifold approximation and projection (UMAP) spaces determined for nuclear RNA (Fig. 3a, top) and chromatin accessibility (ATAC) data (Fig. 3a, middle). We used the shared nearest neighbor (SNN) approach with high resolution parameter to efficiently discriminate among distinct cell types and identified 24 unbiased clusters in the RNA and 25 unbiased clusters in the ATAC dataset, respectively. RNA and ATAC modalities were integrated using the Weighted Nearest-Neighbor (WNN) metrics for downstream analyses and cells projected onto a joint neighbor graph, initially annotated with the RNA clusters (Fig. 3a, bottom).

We next sought to identify the B cell subpopulations within the dataset. To achieve this, we generated a CITE-seq dataset from sorted CD19^+^ B cells of three healthy blood samples (Fig. 3b) that included Antibody Derived Tag (ADT) data of major B cell surface markers, and we used it for the acquisition of cell surface features not provided by the Multiome dataset (Supplementary Table 1). The Seurat tool was used for QC and unsupervised clustering of the new dataset^34,35^ (Fig. 3b and Supplementary Fig. 3d, e). A combination of gene expression and protein markers was used to assign B cell subset identity to the unbiased RNA clusters in the CITE-seq data^15,17,37^ (Fig. 3b, c and Supplementary Fig. 4a, b). Frequencies of somatic mutations in the B cell subsets were consistent with assigned identities and in line with published work (Supplementary Fig. 4c)^38^. We then used a weighted anchor-based approach to transfer ADT measurements from CITE-seq to Multiome and impute protein expression onto the latter based on transcriptomic similarities between the two datasets (Fig. 3a–e)^39^. Imputed protein markers together with gene signatures from Multiome (Supplementary Fig. 4d, e), CITE-seq and published data (Supplementary Data 4 and Supplementary Fig. 4f–h), were used to assign a B cell identity to unbiased RNA clusters within the multiomic dataset^15,17,37,40^ (Fig. 3d). Major B cell populations were identified and agreement between normalized mean expression of observed and imputed ADT markers was observed for cell allocated subsets across the two datasets (Fig. 3c, e). B cell subsets were defined as follows: Transitional B cells as CD10^+++^IgD^+^IgM^+^CD27^-^; naïve cells as CD10^-^IgD^+^IgM^+^CD27^-^; MZB as CD27^+^IgD^+^IgM^++^CD1c^+++^CD45RB^+++^; class-switched memory cells as CD27^+^IgD^-^IgM^-^CD1c^-^CD45RB^++^; IgM-only as CD27^+^IgD^-^ IgM^+^CD1c^+++^; DN2 as CD27^-^IgD^-^CD11c^+^ (Supplementary Table 2). Cells on unclear boundaries between better-defined populations, and with no unique features, were excluded from downstream analyses to avoid ambiguity.

Transitional B cells expressing the *MME* gene, encoding CD10, in the snRNAseq of the Multiome, along with the imputed CD10 protein^33^ (Fig. 3f) were subclustered (Supplementary Fig. 5a) and further subclassified according to the imputed expression of CD21, lowest on T1 cells^17^, and imputed expression of IgM to enable identification of T2M^hi^ and T2M^lo^ cells (Fig. 3g, h and Supplementary Fig. 5b). A mixed population of T2M^hi^ and T2M^lo^ cells was labeled as T2M^mid^ (Fig. 3g) and excluded from the next stages, since accurate unbiased separation of cells in this group was not achievable. This was consistent with the approach used for B cell sorting in bulk ATAC-seq analysis and in other studies in recognition of indistinct boundaries between T2M^hi^ and T2M^lo^ cells^17^ (Fig. 1a). Since surface expression was imputed, it was important to validate the gene expression of T2M^hi^ and T2M^lo^ cells by comparison of snRNA with published gene signatures of these cells^17^ (Fig. 3i). T2M^hi^ cells showed higher levels of *CD1C*, *MZB1*, and *PLD4*, while T2M^lo^ cells were characterized by higher expression of *KLF2* and *CCR7*. The analysis also confirmed previously observed signatures of migratory biases of T2M^hi^ and T2M^lo^ cells, as indicated by the higher expression of genes encoding *ITGB7* and *SELL* on T2M^hi^ and T2M^lo^ cells, respectively^17^.

Identified T1, T2M^hi^, and T2M^lo^ transitional B cells had different epigenetic profiles with DARs consistent with those previously identified in bulk ATAC-seq analyses (Fig. 3j). SnATAC data uncovered additional differences between T2M^hi^ and T2M^lo^ cells (Fig. 3j and Supplementary Data 5). The investigation of top DARs located at the TSS of coding genes for T2M^hi^ cells revealed a greater accessibility at the genomic *loci* of *MNDA*, *ZNF804A*, *CSGALNACT1, TTN, ANK2, PTPRJ, MAST4* and *JAM3*. Top DARs for T2M^lo^ cells included the transcriptional regulator *ZBTB16*, along with *LARGE1, SYN3 and IL7R*. We observed that most genes associated with top DARs in TSSs identified here and in bulk ATAC-seq were not yet transcribed or were expressed at a very low level, suggesting that accessible chromatin regions identified at this stage only had the potential to be actively transcribed upon receipt of additional signals (Fig. 3k, l and Supplementary Fig. 5c and Supplementary Data 6). T2M^mid^ cells showed an intermediate epigenetic and transcriptional landscape in their comparison with other transitional cells for identified features, consistent with mixed population composition (Supplementary Fig. 5c, d).

We undertook Gene Ontology (GO) enrichment analysis of differentially accessible or differentially expressed genes (DEG) to identify any differences in pathways associated with T2M^hi^ and T2M^lo^ subsets (Supplementary Fig. 5e, f and Supplementary Data 7). Major enriched pathways included B cell differentiation and activation for the two populations, albeit involving dissimilar gene subsets, either for ATAC or RNA assay. Of note, genes contributing to over 15% of pathways identified for T2M^hi^ cells included the Toll-like receptor (TLR) family members *TLR*1,4,5,6, 7 or 9. No TLR signatures were evident for T2M^lo^ cells, instead, though in general fewer pathways were identified for this population. Genes associated with T2M^hi^ cell differentiation or activation included *MZB1*, *MNDA*, and *PTPRJ*, already identified as top features for this population (Fig. 3j and Supplementary Data 5 and 6). In contrast, class II-associated genes, including *CD74* and HLA class II alleles, were strongly associated with T2M^lo^ cells in both gene expression and ATAC assays alongside *IL4R* and *CCR7*, consistent with potential for cognate interaction with T cells and recirculation. GO identified the involvement of *SELL* in T2M^lo^ cell migration and adhesion and *ZBTB16* in cell activation. Of note, *SELL*, *ZBTB16, IL4R, CD74, and CCR7* were associated with T2M^lo^ cells earlier in the study (Fig. 3i, j and Supplementary Data 5 and 6).

Thus, we unveiled divergent chromatin states among early B cells, including *loci* not associated yet with gene transcription and not identifiable by transcriptomic analyses alone, but potentially correlated with distinctive functional capacities.

### Progression of T2M^lo^ to naïve B cells is marked by the transcriptional activation of euchromatin regions

**Fig. 4:**
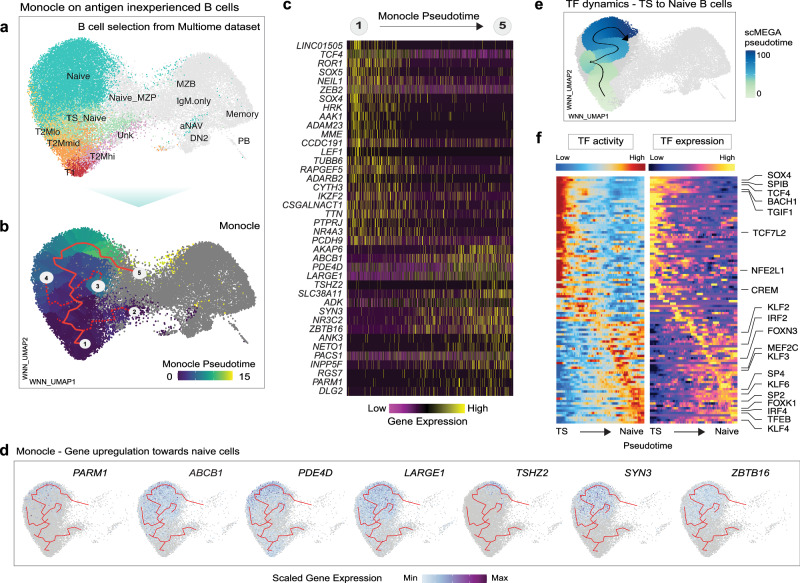
Trajectory analysis of antigen inexperienced B cells suggests developmental links between T2M^lo^ and naïve cells. **a** WNN UMAP of B cells from Multiome dataset highlighting cells selected for Monocle trajectory analysis, colored by cell type. **b** Pseudotime trajectory analysis of antigen inexperienced B cells by Monocle. Cells are color-coded by Monocle pseudotime. The trajectory node with earliest pseudotime is indicated as “1”. Remaining numbered circles represents end nodes of trajectories identified by Monocle. Pseudotime is overlaid on the WNN UMAP plot on a gradient color scale. **c** Heatmap of top B cell marker genes that showed changes in expression along the Monocle pseudotime trajectory from node 1 towards node 5. Statistical analysis was performed with Moran’s I test with Benjamini-Hochberg method for multiple testing. **d** Feature plots of selected genes with dynamic rates of transcription from T1 towards naïve cells, as detected by Monocle. **e** Analysis of TF activity by scMEGA along trajectories identified by Monocle. scMEGA inferred pseudotime based on paired RNA and ATAC profiles of selected cells from T1 towards naïve B cells. The arrow indicates the direction of TF dynamics investigation. **f** Heatmap of TF motif accessibility deviation scores (left) and TF expression (right) over the pseudotime trajectory defined by scMEGA using Pearson’s correlation analysis. Selected top differentially active TFs are annotated. WNN weighted-nearest neighbor; T1 transitional 1 cell T2M^hi^ transitional IgM^high^ T2 cell, T2M^lo^ transitional IgM^low^ T2 cell, MZP marginal zone precursor, MZB marginal zone B cell, aNAV activated-naïve cell, DN2 double negative 2 cell, IgM.only IgM-only, PB plasmablast, Unk unknown, TF transcription factor.

It has been suggested that T2M^lo^ B cells mature to become naïve B cells^41^. To investigate this, the Monocle package was used to identify sequential changes in gene expression in CD27^-^ antigen inexperienced B cells between neighbors in the WNN UMAP space, starting from T1 cells^42–44^ (Fig. 4a, b). Differentiation of T2M^hi^ to MZB was not analyzed in the same way, as this has been studied before^17^, and because MZB maturation is known to involve cellular activation in secondary lymphoid tissues, which would not allow comparable investigation of gene transcription dynamics^6,17^.

Monocle identified two early divergent branches extending from T1 (Fig. 4b, node 1) towards naïve cells via T2M^lo^ cells (Fig. 4b, towards nodes 3, 4 and 5), or towards T2M^hi^ (Fig. 4b, node 2). Genes along the longest identified connection between node 1 and 5 were identified. Genes with increasing expression included some that had previously been identified by their significantly enriched open chromatin state at their genomic *loci* but lack of expression in T2M^lo^ cells, including *PARM1, PDE4D, LARGE1, SYN3*, and *ZBTB16*, along with the naïve B cell marker *ABCB1* (Fig. 4c, d)^45^. This was consistent with a temporal switch from chromatin accessibility only to active gene expression during maturation from T2M^lo^ to naïve cells. In contrast, a progressive silencing of genes associated with early transitional state, such as *SOX4* or *MME*, and genes previously identified as T2M^hi^ ATAC features (*CSGALNACT1, TTN, PTPRJ*), was observed along the same path (Fig. 3j, Fig. 4c and Supplementary Data 8).

To understand the regulatory influence of transcription factors (TF) on early B cell development, cells identified along the Monocle trajectories from T1 to naïve cells were analyzed using the scMEGA package for regulatory network inference, and an scMEGA pseudotime plot was generated according to the earliest and latest nodes identified by Monocle (Fig. 4e). We observed a progressive chromatin closing at the binding motifs of the TFs SOX4, associated with B cell immaturity^41^, and TCF4 associated with T2M^hi^ cells^17^, in line with previous analyses and consistent with progressive enhancer decommissioning, along with increased expression and motif enrichment of members of the FOX and KLF families of TFs, including FOXK1, FOXN3 and also KLF2, a known regulator of naïve B cell function^17,46–53^ (Fig. 4f and Supplementary Data 8).

### Epigenetic landscape across diverse mature B cell subtypes matches transcriptional diversity and supports developmental links with precursors

**Fig. 5:**
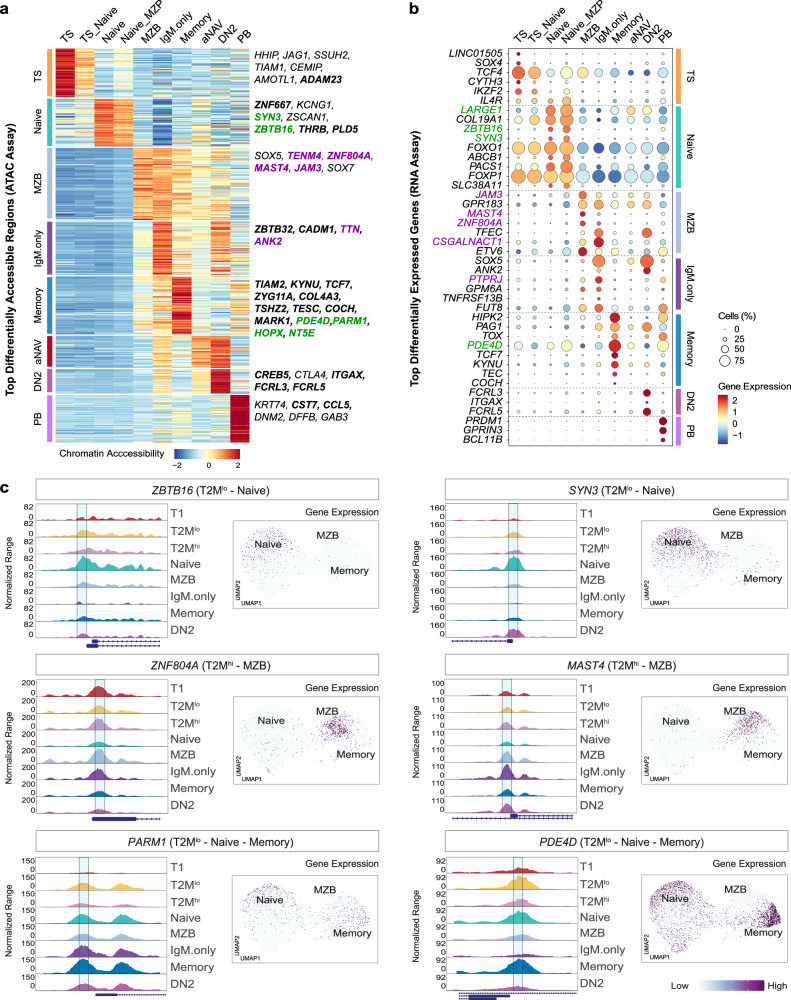
Multiome analysis across B cell subtypes highlights links between mature and transitional cells. **a** Pseudo-bulk heatmap of z-score normalized chromatin accessibility for top 50 DARs of major labeled B cell subpopulations. For each population, key genes associated with top DARs located at TSSs of protein-coding genes are listed on the right-hand side of the heatmap. Genes that showed association between higher chromatin accessibility and increased transcript abundance for the same population are highlighted in bold. Genes that previously shown increased chromatin accessibility in T2M^hi^ or T2M^lo^ cells over other transitional cells (Fig.3j) are highlighted in purple and green, respectively. **b** Dot plot of z-score normalized mean expression of selected top DEGs of major labeled B cell subpopulations. Dot size represents the percentage of cells expressing the gene, the color code represents the average gene expression per subset. **c** Chromatin accessibility profile of DARs at the TSS of selected genes for naïve, MZB and memory cells, showed among multiple B cell subtypes, flanked by feature plots of associated genes, overlaid on WNN UMAP. TSS transcriptional start site, TS transitional, T1 transitional 1, T2M^hi^ transitional IgM^high^ T2 cell, T2M^lo^ transitional IgM^low^ T2 cell, MZP marginal zone precursor, MZB marginal zone B cell, IgM-only IgM-only, PB plasmablast.

To investigate potential relationships between antigen-inexperienced and more mature B cell states, we analyzed the epigenetic and transcriptomic features of all labeled B cell subsets in the Multiome dataset (Fig. 5a,b and Supplementary Data 9, 10). Mature populations were characterized by distinct epigenetic profiles (Fig. 5a) and showed an association between chromatin accessibility at TSSs and gene expression (Fig. 5a, b), revealing a diverse relationship between chromatin remodeling and gene transcription in comparison to transitional cells. Top epigenetic and transcriptomic features were considered as illustrative of B-cell profiles. These showed a homogeneous intra-subset expression, as indicated by the investigation of unbiased clusters identified in snATAC and snRNA datasets (Supplementary Fig. 6a–d). Antigen-inexperienced B cells were epigenetically more similar to each other than to any of the more differentiated subsets, which were themselves highly diverse. A gradual increase in chromatin accessibility was observed for ATAC clusters of naïve cells up to cluster a7, suggestive of a progressive maturation of these cells and consistent with Monocle pseudotime (Supplementary Fig. 6a, c). Of the more mature subsets, notably, MZB were distinctive but had some epigenetic features shared with IgM-only and memory cells. DN2 cells shared very similar DARs with activated naïve cells (aNAV), consistent with their observed developmental relationship^40^. Memory B cells had a strong epigenetic identity, with few other strong resemblances. Plasmablasts had a different chromatin landscape from all other B cells (Fig. 5a).

When the most abundant nuclear transcripts were analyzed, the antigen-inexperienced subsets again resembled each other most closely. MZB, IgM-only and memory B cells were distinctive and different from each other (Fig. 5b and Supplementary Fig. 6b, d, e). A progressive increase in the transcription of top genes for naïve cells was observed towards cluster r2, in line with their epigenetic profile (Supplementary Fig. 6d).

Importantly, the simultaneous investigation of features from transitional subsets to mature B cells enabled the identification of an increased transcriptional abundance of genes that were first identified as discriminating attributes between transitional cell subpopulations (Fig. 3j, Fig. 5b and Supplementary Fig. 5c).

Statistically significant DARs and DEGs for memory cells included *PARM1*, *NT5E*, PDE4D and HOPX (Fig. 5b, c and Supplementary Data 9, 10) that were amongst the top DARs associated with T2M^lo^ in comparison to other transitional cells (Fig. 1c, d and Fig. 3j, l), and that became expressed during maturation from T2M^lo^ to naïve B cells (Fig. 4c,d).

Likewise, *SYN3*, ZBTB16 and LARGE1 were among genes showing a progressive increase in terms of chromatin accessibility and gene expression from T2M^lo^ to naïve B cells, further supporting their potential link with B cell development (Fig. 3j, Fig.4c, d and Fig. 5a–c).

On the other side, *ZNF804A*, *MAST4*, *JAM3, CSGALNACT1* and *PTPRJ*, among others, were genes showing increasing DNA accessibility and subsequent coupling with gene transcription in the transition from T2M^hi^ to MZB cells (Fig. 3j, Fig. 5a–c and Supplementary Data 9, 10).

**Fig. 6:**
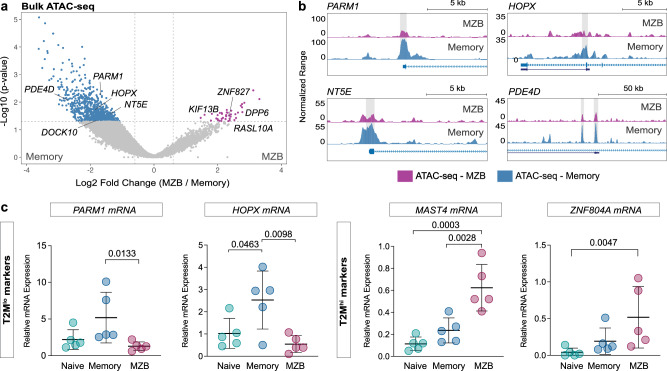
Validation of epigenetic features and transcriptomic detection of identified genes in mature B cells. **a** Volcano plot of DARs in the comparison of sorted memory (blue) and MZB (magenta) cells by bulk ATAC-seq. Statistical analysis was performed using the DESeq2 method with Benjamini-Hochberg for multiple testing. Colored dots represent statistically significant DARs (p-val <0.05 and abs(log_2_FC) > 0.06). Key peaks located at the TSS of protein-coding genes are highlighted in the plot. **b** ATAC-seq profile of major DARs for memory cells in the comparison between memory and MZB cells from bulk ATAC-seq. **c** Bulk RT-qPCR analysis of representative T2M^lo^ markers, *PARM1* and *HOPX*, and T2M^hi^ markers, *MAST4* and *ZNF804A*, in sorted naïve, MZB and memory B cells from human blood (*n* = 5 biological replicates). Colored dots represent mean expression of mRNA targets over endogenous control (18S) of three technical replicates. All error bars are mean ± SD. Normality of data distribution was assessed with the Kolmogorov-Smirnov test. Statistical analysis of normally distributed data (*HOPX* and *MAST4*) was performed with ordinary one-way ANOVA with post-hoc Tukey’s test for multiple comparisons; statistical analysis of non-Gaussian data (*PARM1* and *ZNF804A*) was performed with non-parametric Friedman test with Dunn’s correction for multiple comparisons. All statistical tests are two-sided. Statistically significant exact *p*-values are represented in the plots. Source data are provided as a Source Data file. DAR differentially accessible region, MZB marginal zone B cell T2M^hi,^ transitional IgM^high^ T2 cell, T2M^lo^ transitional IgM^low^ T2 cell.

Visualization of chromatin accessibility here and transcriptome here and elsewhere suggest that memory B cells and MZB are different^14,15,17,38^. Here, we propose that these are the contrasting endpoints in B cell differentiation from pathways that begin their divergence as T2M^lo^ and T2M^hi^ cells, respectively. To validate the differences between memory B cells and MZB identified by the Multiome analysis, we used bulk ATAC-seq to compare sorted MZB and memory B cells from human adult blood (*n* = 3, Fig. 6a, Supplementary Fig. 7a–d and Supplementary Table 1). Consistent with the model we propose, regions of chromatin that were differentially accessible in memory cells compared to MZB included the same regions that were selectively open but not yet transcribed in T2M^lo^ cells when compared with T2M^hi^ (Fig. 6a, b, Fig. 1c, d and Supplementary Data 11).

To validate the differences in expression of representative genes initially identified as T2 markers (*PARM1*, *HOPX*, *MAST4* and *ZNF804A*, Fig. 1c, d, Fig. 3j, l and Fig. 5a, b) in mature cells, we performed bulk RT-qPCR in sorted naïve, memory and MZB populations from human adult blood (Fig. 6c and Supplementary Fig. 7e, f). *PARM1* and *HOPX* showed the highest expression in memory cells, while *MAST4* and *ZNF804A* showed a higher transcript abundance in MZB cells, consistent with previous observations (Fig. 6c). These genes were undetectable after 45 cycles of RT-qPCR analysis of sorted transitional B cell subsets.

### Distinct relationships between epigenome and gene transcription are observed in B cells at different stages of maturation

The model we propose suggests a dynamic evolution from chromatin accessibility to active transcription for genes potentially linked with B-cell maturation. To investigate this further, we used the GeneActivity function of the Signac package^39^ to identify protein-coding genes exhibiting higher chromatin accessibility at the gene body and promoter region in each B cell population of the Multiome dataset in comparison to other B cells (Supplementary Data 12). Identified genes were investigated in parallel with those that had previously shown an increased transcript abundance in the same population in comparison to other B cell subsets (Supplementary Data 9). For identified features, gene (chromatin) accessibility was plotted against gene transcription (z-scores) to evaluate the extent of correlation between epigenomic traits and gene transcription in B cells at different stages of development.

**Fig. 7:**
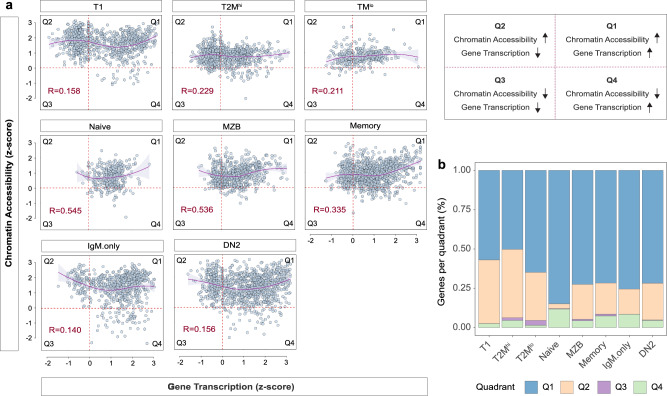
Correlation between gene accessibility and gene transcription in B cell subsets from Multiome analysis. **a** Scatter plots of chromatin accessibility at gene body and promoter region versus gene transcription for statistically significant protein-coding genes from “GeneActivity” and “RNA” assays, respectively, for selected B cell populations in comparison to other B cells. Statistical analysis of data was performed using a Wilcoxon Rank Sum test with Bonferroni correction for multiple comparisons. Gene selection from the two assays was performed based on adjusted *p*-value ≤ 0.05, and to include only genes with more than 20% increase in gene activity or transcription and expressed in at least 5% of cells within the population. X- and Y-axis represent average z-scores of gene transcription or gene accessibility of features in the population of interest. Each dot represents a gene. Genes in common between the two assays are represented only once within the plot. Quadrants in the scatter plots represent the following states: Q1: genes with higher chromatin accessibility and gene expression in comparison to other B cells; Q2: genes showing increased chromatin accessibility, but low or absent gene transcription in comparison to other B cells; Q3: genes with low or no chromatin accessibility or gene transcription; Q4: genes with higher transcript abundance in comparison to other B cells, but no or not differential chromatin accessibility. The non-parametric LOESS method was applied to fit polynomial regression curves of the distribution of points. LOESS curves are represented in magenta. Error bands represent the 95% confidence intervals of y estimates along the x-axes. Exact x and y values and confidence intervals of trend curves are provided in Source Data. A distance correlation analysis was performed to estimate the degree of correlation between gene accessibility and gene transcription for identified features (R: distance correlation coefficient; 0 ≤ R ≤ 1) in each population. **b** Bar plot of gene distribution within the scatter plots in (**a**) for each B cell population. The percentage of genes within each quadrant is indicated on the Y axis. Q quadrant, LOESS locally estimated scatterplot smoothing, T1 transitional 1, T2M^hi^ transitional IgM^high^ T2, T2M^lo^ transitional IgM^low^ T2, MZB marginal zone B, IgM.only IgM-only, DN2 double-negative 2.

A substantial proportion of genes showing high chromatin accessibility not yet associated with gene transcription (Quadrant Q2, Fig. 7a, b) was observed in transitional cells. B cell maturation was characterized by a progressive increase in the fraction of genes showing correlation between gene accessibility and RNA transcription (Quadrant Q1, Fig. 7a, b) or a higher transcript abundance no longer associated with differential DNA accessibility (Quadrant Q4, Fig. 7a, b). This is consistent with a temporal model in which chromatin opening begins in transitional cells (Q2), followed by transcriptional induction during maturation (Q1) and gene transcription persistence after chromatin closing in more mature cells (Q4). The complexity of the observed interactions was denoted by the low distance correlation coefficients identified for each population (R, Fig. 7a), although slightly higher values were observed for naïve, MZB and memory cells in comparison to TS populations.

### Diverse families of transcription factors exhibit differential activity in B cell subsets

**Fig. 8:**
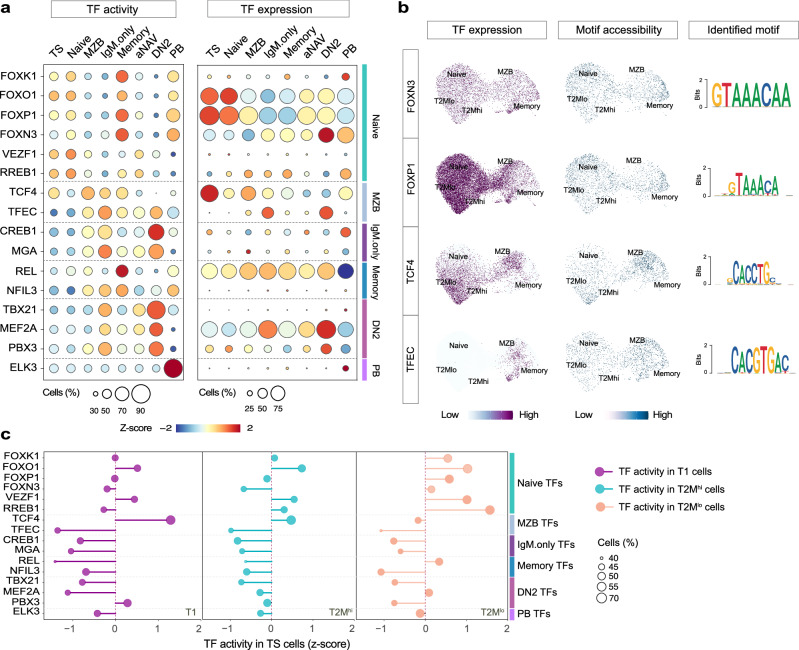
Investigation of Transcription Factor activity and expression in B cell subsets. **a** Dot plots of differential TF activity (left) or expression (right) in B cell subsets. (**a**, left) Dot plot of differential motif accessibility based on ChromVAR z-score for selected TFs from mature B cell populations. Differentially accessible motifs were identified using a Wilcoxon Rank Sum test with Bonferroni correction. TFs were selected based on their differential activity in each B cell subset in comparison to other B cells. Dot size represents the fraction of cells with genomic accessibility for a TF binding motif in each subset. Color code represents the average z-score for TF activity (motif accessibility) for each subset. (**a**, right) Dot plot of z-score normalized mean gene expression of TFs that showed a differential activity in B cell subsets. Dot size represents the percentage of cells expressing the TF in each subset. Color code represents the average z-score of TF expression in each subset. Related B cell subsets are indicated on the right-hand side of the plots. **b** Representative TFs from naïve or MZB populations. Feature plots comparing TF expression and motif accessibility in the Multiome dataset, projected onto the WNN UMAP space. Enriched binding motifs identified in the ChromVAR analysis are indicated on the right-hand side for each TF. **c** Cleveland dot plot of z-score normalized TF activity in transitional B cells for TFs identified for mature B cells and shown in (**a**). Dot size represents the percentage of cells with genomic accessibility for a TF binding motif in each transitional B cell subset. Bar length corresponds to average z-score normalized motif accessibility in each transitional B cell subset. Mature B cells subsets which shown differential activity for selected TFs in (**a**) are indicated on the right-hand side of the plot. TS transitional, MZB marginal zone B cell, aNAV activated-naïve cell, IgM.only IgM-only, DN2 double negative 2 cell, PB plasmablast, TF transcription factor.

To interrogate the underlying regulatory pathways across B cell populations, we investigated the differential activity of TFs using ChromVAR^54^ (Fig. 8a–c and Supplementary Data 13). A dynamic association between TF activity (motif accessibility) and TF expression in blood was observed among B cell subpopulations (Fig. 8a, b). Although broadly expressed in all immature B cells, identified FOX factors (FOXK1, FOXO1, FOXP1, FOXN3) showed a higher activity in naïve B cells, and in T2M^lo^ among transitional cells (Fig. 8c). Binding motif accessibility for these factors persisted in memory cells, despite a reduction in TF expression (Fig. 8a, b). On the other side, TCF4 was identified as a major transcriptional regulator for MZB cells (Fig. 8a, b) and showed a higher activity also in T1 and T2M^hi^ compared to T2M^lo^ cells (Fig. 8c). Other factors were identified as potentially linked to B cell function at mature stages: among these, TFEC binding motif showed increased accessibility in MZB, IgM-only and DN2 cells; REL had greater activity in memory cells (and T2M^lo^ cells among TS cells); targets of TXB21, a known DN2 associated TF^55^, were highly accessible in IgM-only and DN2 cells (Fig. 8a). Expression of identified TFs in blood was sometimes low, despite high binding motif accessibility (Fig. 8a), suggesting the existence of regulatory programs and genomic targets in place prior to or persisting during migration between lymphoid tissues, ready to be activated upon transcription of the related TF.

### Transcription associated with pathway progression in lymphoid-tissue space

**Fig. 9:**
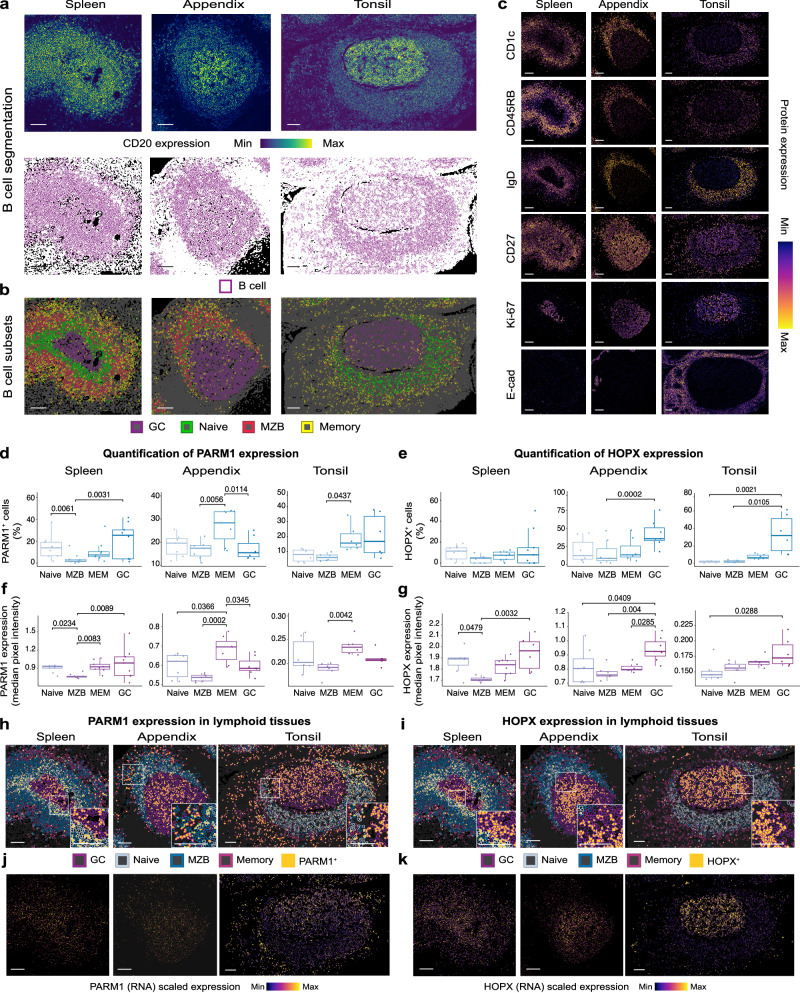
Genes originally identified as differential epigenetic features in transitional cells are transcribed in secondary lymphoid organs. **a** Representative single-channel images displaying CD20 protein expression (viridis color scale) in B cell follicles in spleen, appendix and tonsil (top), and segmented B cells in the same ROIs (bottom). **b** Representative ROIs showing distribution of labeled B cell populations in the secondary lymphoid organs included in the analysis. **c** Single channel images of markers used for B cell subset identification within lymphoid tissues. E-cadherin was included to detect epithelial cells. **d**,**e** Percentage of *PARM1* (**d**) and *HOPX* (**e**) -positive cells in secondary lymphoid organs. Each dot represents the percentage of positive cells for a single ROI (number of technical replicates: *n* = 9 for spleen, *n* = 7 for appendix, *n* = 6 for tonsil; three biological replicates per tissue). Statistical analyses were performed by Friedman test with Dunn’s correction for multiple comparisons. Statistically significant values are represented as exact *p*-values in the plots. **f**,**g** Quantification of *PARM1* (**f**) and *HOPX* (**g**) expression in *PARM1* and *HOPX*-positive cells. Each dot represents the median pixel intensity (normalized counts) in positive cells in a single ROI. Only ROIs containing positive cells in each B cell subset were included (number of technical replicates: *n* = 8 for spleen, *n* = 7 for appendix, *n* = 6 for tonsil; three biological replicates per tissue). Statistical analyses were performed by Kruskal-Wallis test with Dunn’s correction for multiple comparisons. Statistically significant values are represented as exact *p*-values in the plots. (**h**,**i**) Visualization of *PARM1* and *HOPX* expressing cells within selected ROIs from secondary lymphoid organs. B cell subsets are identified with outline color. Yellow fill identifies *PARM1* or *HOPX*-positive cells. **j**,**k** Location and scaled pixel intensity of signal from *PARM1* and *HOPX* mRNA probes in the same ROIs shown in (**h**,**i**), visualized with a gradient color scale. Horizontal central lines in box plots represents the median of observations; box plots extend from the first to the third quartile; whiskers extend to points within 1.5 times the interquartile range from the box. All statistical tests were two-sided. Source data are provided as a Source Data file. All scale bars represent 100 μm. ROI region of interest, MZB marginal zone B cell, GC germinal center.

We investigated human spleen, appendix and tonsil as examples of lymphoid tissues with different anatomical structure and different B cell subset components, including GCs. Imaging mass cytometry (IMC) incorporating RNAscope was performed to validate the expression of *PARM1* and *HOPX*, chosen as exemplars of genes that were consistently identified by all epigenetic methodologies and in all samples as being in regions of accessible chromatin in immature T2M^lo^ cells, and whose expression subsequently became enabled in more mature populations. IMC image pre-processing, segmentation and preliminary IMC data analysis were performed following the BodenmillerGroup pipelines^56^. B cells were initially identified among segmented cells by their expression of CD20 (Fig. 9a and Supplementary Fig. 8a). The Harmony algorithm on normalized counts was used for batch correction and data integration. Major subsets of B cells from spleen, appendix and tonsil were then identified using the SNN graph method, followed by annotation based on expression of major B cell markers^57,58^ (Supplementary Fig. 8b–j and Supplementary Table 1). Identified subsets were visualized in the selected regions of interest (ROI, Fig. 9b, Supplementary Fig. 8k and Supplementary Data 14), showing a distribution consistent with expression of individual B cell markers (Fig. 9c).

*PARM1* and *HOPX*-expressing B cells of each subset were identified within each of the tissues. Normalized counts were used for quantification of marker expression, setting a threshold at the third quartile of count distributions to discriminate between positive and negative cells (Supplementary Fig. 8l). The percentage of positive cells and the signal intensities within positive cells were quantified. In each tissue, memory cells and GC cells had significantly greater expression of each gene, either in terms of a higher proportion of *PARM1* and *HOPX*-positive cells in comparison to other B cell subsets (Fig. 9d, e) or higher transcript expression, as detected by investigation of median signal intensity of *PARM1* and *HOPX* in positive cells (Fig. 9f, g). The microanatomical location of identified positive cells within the tissues (Fig. 9h, i and Supplementary Fig. 8m) was consistent with the distribution of signal from *PARM1* and *HOPX* RNA probes (Fig. 9j, k), and with the organization of memory and GC cells within lymphoid organs^14,58^ (Fig. 9b, h, i).

Furthermore, a higher expression of *PARM1* and *HOPX* in naïve and memory cells compared to MZB was confirmed by bulk RT-qPCR on sorted B cells from human spleen cell suspensions (*n* = 5, Supplementary Fig. 9a–c), consistent with observations from above and published work^15^.

Our data are consistent with a model of B cell bifurcation beginning at the T2 stage of development in which regions of chromatin that are differentially accessible but not yet expressed in T2M^lo^ cells, become actively transcribed genes in mature canonical memory cells, which retain these transcripts in lymphoid tissues. These features help to discriminate memory B cells from MZB in the same microenvironments. Overall, the chromatin signatures of early pathway bifurcation extend progressively through the data, identifying distinct mature B cell endpoints in lymphoid tissue space.

## Discussion

Here, using three different methods to analyze chromatin configuration, we report differences in the epigenetic landscape of immature B cells as the population splits to form two developmental branches. Using undirected analytical methods, we observe similar epigenetic differences between cells in these branches, regardless of whether the cells were produced by a fetus or an adult, suggesting that this is fundamental programming rather than driven by antigen experience.

Genomic regions that were accessible to transposase and H3K4me3-enriched, but not yet transcribed in T2M^lo^ cells, subsequently became expressed by naïve B cells. Those features persisted in class-switched memory cells but were not observed in MZB cells, the mature cell endpoint of the opposing, T2M^hi^, pathway. Interestingly, the genes selected for identification in tissues as representatives of DARs observed in T2M^lo^ and naïve cells, were expressed by memory cells in blood and by both memory and GC cells in lymphoid tissues. When naïve B cells encounter specific antigen that has a protein component, they can undergo cognate interaction with primed T cells that ultimately results in GC formation and the production of memory B cells and plasmablasts^59,60^. It is possible that the DARs they all share are features that are retained through this line of differentiation during a classical adaptive B cell response.

We cannot as yet ascribe detailed functions related to B cell differentiation to the individual regions of open chromatin and gene expression profiles in the branches of B cell differentiation we observed, though the GO analysis of TS features suggested a potential involvement of those markers in B cell differentiation and activation, and some have been linked to B cell function previously^61–64^. The epigenetic landscapes we describe are robustly consistent between methodologies and show similar dynamic changes and associations with expression that are distinct between the different developmental pathways. It will be important to better resolve function in the future. Comparison of potential functions of T2M^hi^ and T2M^lo^ cells by GO analysis, identified signatures of the innate response through accessibility of *loci* encoding TLRs in T2M^hi^ cells. It is known that patients with defective innate signaling through IRAK-4, MyD88, and TIRAP have depleted MZB populations and reduced ability to respond to T-independent antigens^16,65^. On the other hand, pathway analysis identified IL-4 and class II MHC association with T2M^lo^ cells, consistent with the potential for cognate interactions with T cells and subsequent memory cell formation^66^. Open chromatin and gene expression at the CCR7 locus in T2M^lo^ cells is also consistent with the potential to recirculate between tissues, a feature of the naïve B cell population that forms the follicular mantle zone^67,68^.

A distinctive feature of MZB and their precursors is the high density of IgM they express^15,17,69^. In addition to being the first antibody to be produced after a primary immunization, IgM is a primitive immunoglobulin class phylogenetically^70^. Its pentameric structure when in blood, permits high avidity binding despite relatively low antigen affinity. Similarly, a high density of IgM expression on the surface of MZB would be expected to have the same biases and to contribute to the initiation of MZB responses to repeating antigenic structures, which include capsular polysaccharides such as those expressed by pneumococci^6,7,71^. We therefore propose that the MZB developmental pathway in humans is more primitive than the classical adaptive IgM^lo^ branch, and that receptor density contributes to the key role it plays in innate-like anti-bacterial responses.

The relative frequencies of MZB and naïve B cells can differ in disease states. In systemic lupus erythematosus (SLE), for example, MZB, their precursors, and T2M^hi^ cells become depleted, and a similar profile is observed in COVID19, consistent with developmental connection^17,72,73^. This suggests that the split between T2M^lo^ and T2M^hi^ is modifiable, and it may be possible to manipulate it for translational benefit to bias towards either T-dependent or T-independent functional capability, depending on the challenge. Although initially programmed to be enriched in T2M^hi^ cells that, like MZB, might be expected to be relatively more effective at recognition of bacterial antigens, a bias towards the T2M^lo^ pathway in SLE, for example, could reflect a cytokine-driven redirection of differentiation.

Our study has limitations. Gating strategies can be subjective for markers with indistinct boundaries, such as CD10. In addition, the gating strategy used to isolate MZB cells and memory B cells for analysis of regions of open chromatin may contain several B cell subsets with distinct antigen experience histories and epigenomic programming. This will reduce clarity in the data. In addition, our study was undertaken using anonymous samples of healthy blood and lymphoid tissues. Thus, we have no information on demographics. We have not undertaken any functional validation of the progressions we propose due to the unavailability of cells in B cell subsets in sufficient numbers. The data we provide, however, identifies where differences between groups of cells exist in healthy steady state that will allow further interrogation of defects in disease or on challenge in the future.

Historically, dynamic properties of epigenetic profiles have contributed to lineage definition of different immune cells^74^. Here, we show that regions of chromatin accessibility discriminate between human B cell developmental pathways starting from T2M^lo^ and T2M^hi^ cells even before birth. Understanding this and how B cell responses might be shaped by modifying this landscape, could vastly alter the way we view the capacity of B cells to respond to bacterial and viral antigens and the B cell mediators of low-affinity or high-affinity antibody responses.

## Methods

### Cell source

This study was approved by the UK Research Ethics Committees administered through the Integrated Research Application System. Cells and tissues from adults were analyzed with Research Ethics Committee (REC) approval number 11/LO/1274, London Camberwell St. Giles Research Ethics Committee. Cord blood was obtained via the Anthony Nolan research tissue bank, approved by the East Midlands - Derby Research Ethics Committee, approval number 25/EM/0038. Suspensions of spleen cells were obtained from anonymous deceased human liver or kidney donors with REC approval 09/H0802/100 with written informed consent. Peripheral blood samples were obtained from anonymous healthy adults. Sex of cell and tissue donors is unknown, and this is not a factor investigated in the study.

Peripheral blood mononuclear cells (PBMC) and spleen mononuclear cells were isolated by density gradient centrifugation using Ficoll-Paque PLUS (Cat.N. 17-1440-03, GE Healthcare). The buffy coat layer was collected, and cells were washed twice with 1xPBS. PBMCs and splenic mononuclear cells were cryopreserved in 50% FBS + 10% DMSO in RPMI-1640 in aliquots of 25 × 10^6^ cells until use.

### Fluorescence-activated cell sorting

Cryopreserved cells used for flow cytometry were thawed, washed and resuspended in RPMI-1640 and then rested at 37 °C for 10 min. For bulk experiments from adult human blood, B cells were isolated by magnetic-activated cell sorting (MACS) using the B cell Isolation Kit II human (Cat.N. 130-091-151, Miltenyi Biotec, Bergisch-Gladbach, Germany) prior to sorting. The gating strategy and staining protocol used for cell sorting was comparable between PBMCs and spleen cell suspensions for all samples included in the study. Cells were blocked on ice with Human TruStain FcX (Cat.N. 422302, BioLegend), diluted 1:50 in FACS buffer (1% FBS in 1xPBS) for 10 min and then stained for 40 min on ice with pre-titrated concentrations of antibodies against major B cell surface proteins (Supplementary Table 1). Cells were washed in FACS buffer and passed through a 70 μm cell strainer to generate a single cell suspension. Anti-Mouse Ig beads (Cat.N. 552843, BD Biosciences) were used for fluorescent compensation, and gates were set based on signals from unstained control cells. Cell sorting was performed using BD FACSAria IIu SORP (BD Biosciences) cell sorter equipped with BD FACSDiva software (v8.0.1), with a 100 µm nozzle and at low-pressure (20 PSI) to avoid cellular stress and increase cell viability. DAPI was added at a final concentration of 0.1 μg/ml to the single-cell suspension immediately prior to sorting to discard dead cells. Total CD19^+^ cells were sorted for single-cell experiments. We acknowledge that there is subjectivity in defining transitional B cells and their subsets. We therefore followed strict rules in the definition of subsets according to marker expression intensities. For bulk ATAC-sequencing, CUT&RUN and RT-qPCR experiments, live single CD19^+^ B cells were gated as follows: at the beginning of sorting, gates were set to capture the cells with the top 20% of CD10 expression within CD10-expressing cells. These were designated T1. Then, gates were set to capture the top 35% and bottom 35% of IgM expression in the remaining CD10^+^ cells. These were designated as T2M^hi^ and T2M^lo^, respectively. Mature B cell sorting was performed on CD19^+^CD10^-^ cells, using gates capturing IgD^+^CD27^+^ MZB cells, IgD^-^CD27^+^ memory cells and IgD^+^CD27^-^ naïve B cells (the latter when included in the experiment). B cell populations of interest were sorted into 200μl of 100% FBS in FBS pre-coated DNA LoBind tubes (Cat.N. 30108051, Eppendorf). Cell sorting for bulk ATAC-seq experiments was performed separately for transitional or mature cell populations. Only mature cells were sorted from spleen cell suspension, using the same gating approach indicated above for mature B cell sorting in blood.

### Bulk ATAC-seq

Sorted cells from each B cell population (*n* = 3 biological replicates) were washed with ice-cold 1xPBS, centrifuged at 500 g for 5 min at 4 °C. Visual assessment of nuclear integrity was performed by the Trypan Blue dye exclusion method. ATAC-seq and library barcoding was performed^75^. Library quality and quantity were assessed using Agilent Qubit and HS DNA tapestation. SPRIselect beads (Cat.N. B23317, Beckman Colter) were used to deplete the unligated adapter. Individually barcoded libraries were then pooled and sequenced on an Illumina NextSeq 2000 instrument with appropriate reagents (Cat.N. 20046811, Nextseq 1000/2000 P2 (100cycles) v3, Illumina).

### CUT&RUN

Similar to ATAC-seq, B cell populations (*n* = 2 biological replicates) were sorted into 100% FBS, then washed with ice-cold PBS and centrifuged at 500 g for 5 min at 4 °C. Nuclei were isolated and subjected to CUT&RUN^75^. An IgG (Cat.N. 12-370, Millipore) isotype control antibody was included and processed in parallel with the H3K4me3 (Cat.N. Ab8580, Abcam)-processed samples to assess background cleavage. Because of the greater fragility of flow-sorted primary B cell nuclei, all incubation times were reduced from those described for this technique^76^. Cell were lysed in cold lysis buffer (10 mM Tris-Cl, pH 7.4, 10 mM NaCl, 3 mM MgCl_2_ and 0.1% IGEPAL CA-630). Pelleted nuclei were resuspended in CUT&RUN Wash buffer (20 mM Tris-HCl pH 7.5, 150 mM NaCl, 0.5 mM spermidine, 0.1% BSA), and nuclei integrity was verified using trypan blue in a hemocytometer. Nuclei were incubated with 1 μg of rabbit anti-H3K4m3 or 1 μg IgG-isotype control antibody (Millipore, Cat.N. 12-370) in CUT&RUN Wash buffer for 10 min with rotation at 4 °C. Nuclei were then incubated without a washing step with pA-MNase (1.6 µg/µl; made by the Protein Production Facility at King’s College London using Addgene Vector no. 86973) at 4 °C with rotation for an additional 10 min. Nuclei were washed and resuspended in CUT&RUN wash buffer. DNA-tethered cleavage was induced by adding CaCl_2_ at a final concentration of 2 mM on ice water for 10 min. The reaction was stopped by adding a Stop buffer (200 mM NaCl, 20 mM EDTA, 4 mM EGTA, 50 µg/ml RNAse A (Cat.N. EN0531, Thermo Scientific) and 40 µg/ml glycogen (Cat.N. R0561, Thermo Scientific) at a 1-to-1 ratio. Nuclei integrity was assessed again. To release the DNA fragments of interest, nuclei were incubated at 37 °C for 20 min. Solubilized chromatin was separated from nuclei by centrifugation at 16,000 g at 4 °C for 5 min and then digested with SDS (0.1% final concentration) and Proteinase K (0.2 mg/ml final concentration) at 70 °C for 10 min. Released DNA fragments were then extracted with phenol:chloroform: isoamyl alcohol (25:24:1) at 16,000 xg for 15 min at room temperature. The aqueous upper layer was transferred to a fresh tube, and NaCl was added at a final concentration of 250 mM. An equal volume of isopropanol was further added, and the DNA was left to precipitate for 2 h at −80 °C. Precipitated DNA was then pelleted by centrifugation (16,000 x g for 15 min at 4 °C), washed with 70% ice-cold EtOH, and resuspended in distilled water. The liberated DNA was subjected to library generation using the NEBNext Ultra II library prep kit and Multiplex Oligo (Cat.N. E765S & Cat.N. E7500S, New England Biolabs), following the manufacturer's protocol and thermocycling temperatures. Briefly, DNA ends from fragments harvested from the CUT&RUN experiment were repaired, 5’ ends phosphorylated and 3’ ends A-tailed prior to ligation with NEBNext Adapter, UNG was then used to linearize the ligated adapter. SPRIselect beads were used to deplete the unligated adapter. Adapter-ligated libraries were then barcoded by PCR following the manufacturer’s recommended protocol. Libraries were amplified for 11 cycles and purified using SPRIselect beads prior to quantitation using Agilent Qubit and HS DNA tapestation. Individually barcoded libraries were then pooled and sequenced on an Illumina NextSeq 2000 instrument.

### RT-qPCR

Total RNA was isolated from FACS–sorted B cell populations (*n* = 5 for human blood; *n* = 5 for human spleen). Complementary DNA (cDNA) was synthesized from RNA using the High-Capacity RNA-to-cDNA Kit (Cat.N. 4387406, Applied Biosystems) according to the manufacturer’s instructions. Quantitative PCR (qPCR) was performed using TaqMan Fast Advanced Master Mix (Cat.N. 4444557, Applied Biosystems) together with predesigned TaqMan Gene Expression Assays (Applied Biosystems) on a QuantStudio 7 Flex Real-Time PCR System (Applied Biosystems). Gene expression was quantified using target-specific TaqMan probes for PARM1 (Hs00209876_m1), HOPX (Hs00261238_m1), MAST4 (Hs00389519_m1), and ZNF804A (Hs00290118_s1). 18S rRNA (TaqMan Endogenous Control Assay 4318839, VIC-labeled) was used as the internal reference gene for normalization. Relative gene expression levels were calculated using the ΔCt method. All reactions were performed in technical triplicate following the manufacturer’s recommended thermocycling conditions.

### Single-nucleus multiome (ATAC + gene expression) sequencing

Cryopreserved PBMCs from four healthy donors were thawed and live single CD19^+^ B sorted into 200μl of 100% FBS, washed with ice-cold 1xPBS and then centrifuged at 500 x g for 5 min at 4 °C. Supernatant was removed and cells were lysed in cold lysis buffer (10 mM Tris-Cl, pH 7.4, 10 mM NaCl, 3 mM MgCl_2_ and 0.1% IGEPAL CA-630)^75^. Isolated nuclei were centrifuged at 500 x g for 10 min at 4 °C. Supernatant was removed and nuclei resuspended in the appropriate volume of 1x Nuclei Buffer (Cat.N. PN-2000153, 10x Genomics) in nuclease-free water to achieve an optimal concentration of ~5000 nuclei/μl. Joint snRNA/ATAC-seq libraries were generated using the 10x Genomics Single Cell Multiome ATAC + Gene Expression kit (Cat.N. 1000285), following the manufacturer’s instructions to achieve a Targeted Nuclei Recovery of 8000 nuclei/sample. Quality control assessment of the libraries was carried out using Agilent TapeStation. Libraries were diluted to 800 nM, pooled, and sequenced on a NextSeq 2000 P3 Flow cell (Illumina) with paired-end 138-bp reads to a target depth of 25,000 read pairs per nucleus for each snRNA-seq library, and 50,000 read pairs per nucleus for each snATAC-seq library.

### Single-cell CITE-seq (RNA + protein expression) sequencing

Cryopreserved PBMCs from three healthy donors were thawed and FACS sorted as described above. Cells were stained at the same time with PE-Dazzle-CD19 antibodies for sorting and with TotalSeq-C (5’ chemistry) oligonucleotide-tagged antibodies (BioLegend, Supplementary Table 1) for major B cell surface markers, diluted in FACS buffer (PBS **+** 2% FBS). Live single CD19^+^ B cells were sorted into 200 μl 100% FBS, washed with ice-cold 1xPBS, centrifuged at 500 x g for 5 min at 4 °C. Supernatant was removed, and cells resuspended in the appropriate volume of FACS buffer to achieve an optimal concentration of approximately 5000 cells/μl. After a quality-control step for evaluation of cell viability, cells were loaded onto a 10X Genomics Chromium controller, and libraries (5′ gene expression, V(D)J, and ADT) were prepared according to the manufacturer’s guidelines. The Illumina HiSeq 2500 High Output platform was used for sequencing (30-100-100 sequencing configuration).

### Bulk ATAC-seq and CUT&RUN analysis

Galaxy.org platform (www.usegalaxy.org) was used for bulk ATAC and CUT&RUN analyses. FASTQ Groomer (v 1.1.5+ Galaxy2) was applied to raw sequence files to ensure they were in the correct format.

FASTQC was applied to assess quality, and the Trim and Clip pipelines were used to remove adapters and low-quality reads. Reads were aligned to the hg38 reference genome using Bowtie (v 2.5.3) with default settings. Uniquely mapped reads were extracted using bamFilter (v 2.4.1) with parameters mapQuality >=30, isProperPair=True, reference = !chrM, and duplicates removed with Mark Duplicates= false (v 2.18.2.2). Peak calling was performed with the MACS2 tools with ‘no mode’ and by setting extension size to 200 and shift size to −100, using a q value of 0.05. Differentially accessible regions (DARs) (ATAC) and differentially enriched regions for H3K4me3 (CUT&RUN) were computed and annotated through DiffBind (v 3.12.0) with the DESeq2 method and ChIPseeker (v 1.28.3). Bamcoverage was applied for visualization of identified regions in UCSC Genome Browser (https://genome.ucsc.edu/index.html).

### Single-cell CITE-seq (RNA + protein expression) data analysis

Raw reads were processed using CellRanger (pipeline version cellranger-arc-2.0.2) for quality control and generation of fastq files for each biological replicate (*n* = 3). Transcripts were aligned to a reference genome (GRCh38) and feature-barcoded matrices generated from scRNA outputs. Gene expression and surface antibody-derived tags (ADT) matrices were read into Seurat (v5.1.0)^34,35^ in R (v4.3.1). Low-quality cells with a low or high number of genes and RNA counts, or with a high percentage of mitochondrial DNA, were removed. Gene expression measurements were normalized and scaled, and cells clustered based on their RNA-seq profiles. Top variable RNA features were used for PCA and dimensionality reduction, and cells were projected onto a UMAP space. RNA clusters were defined using the FindClusters function with default parameters. Surface protein expression measurements were normalized using the centered log ratio transformation (CLR) method, and protein expression was visualized in the previously generated UMAP space. Clusters were manually assigned a B cell label based on the expression of known B cell subset genes and surface protein markers. Differential gene expression among labeled clusters was assessed using the Seurat FindMarkers function with a Wilcoxon Rank-Sum test and Bonferroni correction for multiple comparisons. The BCR repertoire of cells included in the dataset was analyzed using the Immcantation framework (version 4.7.0)^38,77^. V, D, J assignment was performed using IgBLAST and non-productive sequences were removed. Only cells with one productive IGH chain were selected for the identification of clones and calculation of somatic hypermutation in the V gene. BCR mutation frequency values were matched to CITE-seq data by cell ID for data visualization.

### Single-nucleus multiome (ATAC + gene expression) data analysis

Raw reads were processed using CellRanger for quality control and generation of fastq, bam and bed files for each biological replicate (*n* = 4). Reads were aligned to a reference genome (GRCh38) and feature-barcoded matrices generated from snRNA and snATAC-seq outputs. Matrices were read into Seurat and Signac (v1.13.0)^36^. Low-quality cells with low or high RNA and ATAC counts, or with low TSS-enrichment and nucleosome-signal scores, were removed. Efficient chromatin fragmentation and optimal distribution of fragment lengths were observed in all replicates. No mitochondrial contamination was observed.

Gene expression and DNA accessibility data were processed for each donor. Gene expression measurements were normalized with the “LogNormalize” method and scaled. A Principal Component Analysis was performed using default parameters. Latent Semantic Indexing (LSI) was used to normalize and linearly reduce the dimensionality of ATAC matrices, with default parameters.

Integration was performed for RNA and ATAC matrices. The four replicates were merged into a unique dataset. RNA matrices were then integrated using the IntegrateLayers function and the anchor-based RPCA integration method. Layers were joined after integration. For ATAC assay integration, datasets were projected into a shared low-dimensional space using a reciprocal LSI projection (“rlsi”). LSI coordinates (embeddings) across the datasets were then integrated using the IntegrateEmbeddings function. Normalization, scaling, dimensionality reduction and clustering were performed for RNA and ATAC assays in the integrated object and cells projected onto a UMAP space. If needed, clusters were further subdivided to achieve a greater separation. Then, the weighted nearest neighbor (WNN) method was used to compute a joint neighbor graph that represents both gene expression and DNA accessibility measurements, and a joint UMAP was constructed. A total of 36,905 high-quality cells were derived from four samples.

In order to acquire protein expression measurements, the FindTransferAnchors function in Seurat was used to identify a set of anchors between the RNA assay of CITE-seq dataset (reference) and the RNA assay from Multiome (query). Those were used to transfer ADT measurements from CITE-seq to the Multiome dataset with the TransferData function. Finally, Multiome unbiased RNA clusters were assigned a B cell identity using a combination of RNA features and imputed surface protein markers.

For labeled B cells, differential gene expression and chromatin accessibility analyses were performed using the Seurat FindMarkers function, with a Wilcoxon Rank-Sum test and a Bonferroni correction for multiple comparisons. Pseudo-bulk investigation of DARs was performed using the AverageExpression function to return a Seurat object with averaged expression values for each identity class, and the DoHeatmap function was applied to display top DARs among clusters. Single-nucleus motif enrichment was computed for a set of 746 transcription factors from the JASPAR 2020 database, using the Signac wrapper for ChromVAR^54^. Differentially accessible TF motifs were identified using a built-in t-test function with Benjamini-Hochberg for multiple testing, and differential motif accessibility between B cell clusters was identified using a Wilcoxon Rank Sum test with Bonferroni correction for multiple comparisons.

Gene activity analysis for the identification of genes showing differential chromatin accessibility among B cell subsets was performed using the GeneActivity function of the Signac package, extending the investigation of accessible regions to gene bodies and promoter regions (2000 bp upstream of the TSS) of protein-coding genes. Genes with differential accessibility among B cells were identified using the FindMarkers function for the GeneActivity (“GeneAct”) assay^39^, with a Wilcoxon Rank Sum test and Bonferroni correction for multiple comparisons.

Labeled transitional (TS) B cells were split and further sub-classified based on snRNA-seq profiles. Unbiased subclusters were assigned a transitional B cell identity based on a combination of known gene features and imputed surface protein markers. T1 cells were identified as CD10^high^IgM^high^CD21^low^ cells. T2 cells were separated based on the expression of IgM. Unbiased clusters combining cells with differential expression of IgM were assigned a T2M^mid^ identity and excluded from downstream analyses to avoid ambiguity when boundaries were uncertain. Differential gene expression and chromatin accessibility analyses were performed as described above.

Gene Ontology analysis of statistically significant DARs associated with protein-coding genes (ATAC assay) and DEGs (RNA assay) for T2 cell subsets was performed using the enrichGO function of the clusterProfiler (v4.10.1) package in R^78^. DARs used for GO analysis were selected based on their proximity (distance ≤ 2000 bp) to DARs located at the UTRs or coding regions of protein-coding genes. GO enrichment analysis was performed using a one-sided Fisher’s exact test, followed by the Benjamini-Hochberg test for multiple comparisons, as per the package default settings.

Correlation analysis between chromatin accessibility and gene transcription in B cell subsets was performed as follows: average expression values of RNA or gene activity features were calculated using the Seurat AverageExpression function and normalized. RNA and GeneAct assay matrices were extracted from the Multiome dataset and filtered to remove mitochondrial, ribosomal or other non-specific gene patterns. A unique data frame, including features from RNA and GeneAct assays, was then generated, containing mean z-score values for all B cell populations in the Multiome dataset. For each population, the generated data frame was filtered to include only statistically significant DEGs or differentially accessible genes. Statistically significant RNA and GeneAct features in each population were identified using a Wilcoxon Rank Sum test with Bonferroni correction and selected based on adjusted p-value (≤0.05), and to include only those showing more than 20% increase in accessibility or transcription and expressed by at least 5% of the population of interest. Z-scores from RNA and GeneAct assays of selected features were plotted on an XY graph. The geom_smooth() function of the ggplot2 R package was used to run the LOESS (Locally Estimated Scatterplot Smoothing) method for polynomial regression of distributions and fit a LOESS curve with 95% confidence intervals of y estimates (confidence band). Distance correlation analysis to investigate the degree of correlation between gene accessibility and transcription of features of interest was performed in Python using the dcor.distance_correlation() function of the dcor (v0.6) package: the distance correlation coefficient, R, varying from 0 (independent variables) to 1 (dependent variables) was inferred^79^.

### Trajectory analyses on antigen-inexperienced B cells

Trajectory analysis was performed using the Monocle3 package^42^. Cells belonging to transitional (TS), TS_Naive and Naïve subsets were separated from the dataset and converted into a cell_data_set (cds) object. Cluster information and UMAP embeddings were transferred from Multiome to the cds object and used to plot cells in the new dataset. The learn_graph function from Monocle3 was used to learn the sequence of gene expression changes and construct a trajectory graph within the UMAP space. Pseudotime analysis was performed, choosing as root node the one identified in correspondence to T1 cells (node 1). Differential gene expression analysis was performed on branches extending from node 1 towards naïve cells. Temporal investigation of the identified DEGs was performed on cells that developed along the longest trajectory (nodes 1–5) in the original Multiome dataset, with cells ordered by Monocle pseudo-time. The Moran’s I test was used to measure spatial autocorrelation along selected trajectories, followed by the Benjamini-Hochberg method for multiple testing and calculation of false discovery rate (q-value).

Investigation of TF factor dynamics for antigen inexperienced B cells was performed using the scMEGA package^80^ on the same cells included in Monocle trajectory analysis, developing from T1 to naïve cells. Cells from Multiome were selected using their cell IDs to include only cells along the major branches analyzed with Monocle. A pseudo-time trajectory was inferred based on the supervised input and a trajectory plot was built. Candidate TFs with differential activity along the inferred trajectory were selected based on Pearson’s correlation between gene expression and motif accessibility, the latter inferred from previously computed ChromVAR activity scores, using a built-in t-test function with Benjamini-Hochberg for multiple testing.

### RNAscope-imaging mass cytometry sample preparation

A combined RNAscope-IMC protocol was used for simultaneous investigation of proteins and mRNA molecules in spleen, appendix and tonsil tissue sections. RNA probes against PARM1 (Cat.N. 851001-C2, Bio-Techne) and HOPX (Cat.N. 423001, Bio-Techne) were included in the study. Samples were prepared as described in ref. ^58^, in three biological replicates for each tissue. RNAscope was performed on 5 μm thick FFPE sections from each organ using the Multiplex Fluorescent Reagent Kit V2 Assay (Cat.N. 323110, ACDBio) following manufacturer’s instructions. Slides were dewaxed, subjected to antigen retrieval and incubated with probes for candidate transcripts. Each probe was then incubated with the appropriate HRP-tagged oligos for amplification of the signal. PARM1 probes were conjugated with TSA-Biotin, and HOPX probes with TSA-FITC reagents. At the end of the RNAscope protocol, tissues were washed, blocked and incubated with the mix of metal-conjugated antibodies specified in Supplementary Table 1, overnight at 4 °C. PARM1 and HOPX probes were detected with metal-tagged antibodies against Biotin and FITC, respectively. Nuclei detection was performed using the DNA intercalator Iridium Cell-ID Intercalator-Ir (Cat.N. 201192 A, Standard BioTools). Data acquisition was conducted on a Hyperion Imaging System (Fluidigm). Regions of up to 1 mm^2^ were selected and laser-ablated at 200-Hz frequency at 1 μm/pixel resolution.

### Imaging mass cytometry data analysis

IMC data pre-processing and major downstream single-cell data analysis were performed following the Bodenmiller workflow (https://bodenmillergroup.github.io/IMCDataAnalysis/)^58^. Upon image acquisition with the Hyperion System, an MCD data file containing sample metadata and multi-channel images was generated. Images were converted from.mcd to.ome.tiff and.tiff format in Python and cell segmentation was performed via supervised pixel classification in Ilastik (v.1.4.0.post1) and CellProfiler (v4.2.6). Spatially resolved single-cell data of pixel intensities for each channel and cell morphology were quantified and exported for downstream analyses. Images, masks and single-cell data were read in R and the latter stored into a SpatialExperiment object. Pixel intensities were arcsin transformed, and data preprocessing was performed for spillover correction, segmentation and sample quality control. Cell phenotyping was performed with a semi-supervised approach combining manual labeling and the usage of a random forest classifier to assign a phenotype to unlabeled cells, and cells were gated^58^. Only identified B cells were retained for downstream analyses. Pixel intensity matrices were normalized to a range from 0 to 3. Normalized counts were used for dimensionality reduction, batch-effect correction, and data integration in Harmony. Cells were then clustered with the Shared Nearest Neighbor Graph method and manually labeled based on the expression of major B cell markers included in the panel. Cells that could not be assigned a clear identity were excluded from datasets. Naïve, MZB, GC and memory cells were represented in all tissues and were used for downstream analyses. Labeled B cells or scaled pixel intensities for individual channels were visualized on images using the plotCells and plotPixels functions of the cytomapper package, respectively^81^.

Investigation of PARM1 and HOPX expression was performed on normalized counts, with a threshold set at the 3rd quartile of the count distribution in each tissue to distinguish positive from negative cells. Quantification of positive cells in each B cell population was performed for all ROIs included in the spatial experiments. For PARM1 or HOPX-positive cells, PARM1 or HOPX expression was calculated as the median of normalized counts for each ROI. PARM1 or HOPX -positive cells within each subset were labeled and visualized on images, along with B cell identity labels. Scaled pixel intensities for PARM1 and HOPX probes were visualized on the same images to validate the assigned identities.

Metadata of ROIs included in the IMC analysis is provided in Supplementary Data 14.

### Statistics, data analysis, and visualization

Statistical analyses for identification of differentially accessible regions in bulk ATAC-seq and differentially H3K4me3-enriched regions in CUT&RUN were performed in Galaxy using the DESeq2 multi-step method, as the default method in DiffBind for statistical testing. A p-val <0.05 and abs(log2FC) **>** 0.6 were used as cut-off for statistical significance. Statistical analyses of single-cell data were performed in R. Differential analyses for gene expression and chromatin accessibility in single-cell data were performed using a Wilcoxon Rank Sum test with Bonferroni correction, as per default parameters of Signac and Seurat packages. Identification of TFs with differential activity in single-cell data was performed with a ChromVAR built-in parametric t-test for differential analysis, followed by the Benjamini-Hochberg method for multiple testing. Differential motif accessibility between clusters was performed with a Wilcoxon Rank Sum test with Bonferroni correction. Plots for CITE-seq and Multiome data were generated using functions from Seurat and Signac packages and customized with additional ggplot2 layers. Image visualization in IMC data analysis was performed using functions from the cytomapper package. All other plots in the manuscript were generated using the ggplot2 package.

Further two-sided statistical analyses were carried out using Prism v10. Data were tested for consistency with a Gaussian distribution using the Kolmogorov-Smirnov test. If data were normally distributed, they were compared by one-way Analysis of Variance (ANOVA) with Tukey’s post-hoc test for multiple comparisons; if not, they were analyzed by non-parametric Friedman or Kruskal-Wallis test with Dunn’s correction for multiple comparisons.

### Reporting summary

Further information on research design is available in the Nature Portfolio Reporting Summary linked to this article.

## Supplementary information

Supplementary Information

Description of Addtional Supplementary Files

Supplementary Data 1

Supplementary Data 2

Supplementary Data 3

Supplementary Data 4

Supplementary Data 5

Supplementary Data 6

Supplementary Data 7

Supplementary Data 8

Supplementary Data 9

Supplementary Data 10

Supplementary Data 11

Supplementary Data 12

Supplementary Data 13

Supplementary Data 14

Reporting Summary

Transparent Peer Review file

## Source data

Source data

## Data Availability

All data are included in the Supplementary Information or available from the authors, as are unique reagents used in this Article. The raw numbers for charts and graphs are available in the Source Data file whenever possible. The bulk ATAC-seq and H3K4me3 CUT&RUN data originated in this study have been deposited in the GEO database under accession code GSE326191 (https://www.ncbi.nlm.nih.gov/geo/query/acc.cgi?acc=GSE326191). The single-cell CITE-seq and Multiome data originated in this study have been deposited in the GEO database under accession codes GSE321688 (https://www.ncbi.nlm.nih.gov/geo/query/acc.cgi?acc=GSE321688) and GSE322590 (https://www.ncbi.nlm.nih.gov/geo/query/acc.cgi?acc=GSE322590), respectively. The IMC data generated in this study have been deposited on Zenodo under record 17541644 (https://zenodo.org/records/17541644). Source data are provided with this paper.

## Code availability

Custom codes used in this paper are available at github.com/jspencer-lab/Bcell_Epigenetics_2026 and have been deposited on Zenodo under record 20274604 (https://zenodo.org/records/20274604).
