# Supplementary material for: Epigenetic signatures mark early peripheral human B lineage bifurcation and differential transcriptional profiles in mature populations
Source: Nat Commun. 2026 Jul 14;17:8628. doi: 10.1038/s41467-026-75364-3 (PMC13487194; doi:10.1038/s41467-026-75364-3)
Supplement: Supplementary file 1 [file 41467_2026_75364_MOESM17_ESM.pdf]

# nature portfolio

Corresponding author(s): Jo Spencer

Last updated by author(s): 15MAY 2026



# Reporting Summary

Nature Portfolio wishes to improve the reproducibility of the work that we publish. This form provides structure for consistency and transparency in reporting. For further information on Nature Portfolio policies, see our Editorial Policies and the Editorial Policy Checklist.

## Statistics

For all statistical analyses, confirm that the following items are present in the figure legend, table legend, main text, or Methods section.

| n/a | Confirmed | |
|---|---|---|
| ☐ | ☒ | The exact sample size ($n$) for each experimental group/condition, given as a discrete number and unit of measurement |
| ☐ | ☒ | A statement on whether measurements were taken from distinct samples or whether the same sample was measured repeatedly |
| ☐ | ☒ | The statistical test(s) used AND whether they are one- or two-sided<br>*Only common tests should be described solely by name; describe more complex techniques in the Methods section.* |
| ☐ | ☒ | A description of all covariates tested |
| ☐ | ☒ | A description of any assumptions or corrections, such as tests of normality and adjustment for multiple comparisons |
| ☐ | ☒ | A full description of the statistical parameters including central tendency (e.g. means) or other basic estimates (e.g. regression coefficient) AND variation (e.g. standard deviation) or associated estimates of uncertainty (e.g. confidence intervals) |
| ☐ | ☒ | For null hypothesis testing, the test statistic (e.g. $F$, $t$, $r$) with confidence intervals, effect sizes, degrees of freedom and $P$ value noted<br>*Give P values as exact values whenever suitable.* |
| ☒ | ☐ | For Bayesian analysis, information on the choice of priors and Markov chain Monte Carlo settings |
| ☒ | ☐ | For hierarchical and complex designs, identification of the appropriate level for tests and full reporting of outcomes |
| ☒ | ☐ | Estimates of effect sizes (e.g. Cohen's $d$, Pearson's $r$), indicating how they were calculated |

*Our web collection on statistics for biologists contains articles on many of the points above.*

## Software and code

Policy information about availability of computer code

| | |
|---|---|
| Data collection | Data availability statements are included in the manuscript |
| Data analysis | Custom codes used in this paper are available at github.com/jspencer-lab/Bcell_Epigenetics_2026 and have been deposited on Zenodo under record 20274604. |

For manuscripts utilizing custom algorithms or software that are central to the research but not yet described in published literature, software must be made available to editors and reviewers. We strongly encourage code deposition in a community repository (e.g. GitHub). See the Nature Portfolio guidelines for submitting code & software for further information.

## Data

Policy information about availability of data

All manuscripts must include a data availability statement. This statement should provide the following information, where applicable:

- Accession codes, unique identifiers, or web links for publicly available datasets
- A description of any restrictions on data availability
- For clinical datasets or third party data, please ensure that the statement adheres to our policy

The bulk ATAC-seq and H3K4me3 CUT&RUN data originated in this study have been deposited in the GEO database under accession code GSE326191. The single-cell CITE-seq and Multiome data originated in this study have been deposited in the GEO database under accession codes GSE321688 and GSE322590, respectively. The IMC data generated in this study have been deposited on Zenodo under record 17541644. Source data are provided with this paper.

## Research involving human participants, their data, or biological material

Policy information about studies with human participants or human data. See also policy information about sex, gender (identity/presentation), and sexual orientation and race, ethnicity and racism.

| | |
|---|---|
| Reporting on sex and gender | Samples used in this study are from anonymous donors. This information is not available. |
| Reporting on race, ethnicity, or other socially relevant groupings | Samples used in this study are from anonymous donors. This information is not available. |
| Population characteristics | Samples used in this study are from anonymous donors. This information is not available. |
| Recruitment | Samples used in this study are from anonymous donors. |
| Ethics oversight | All aspects of the use of human tissues has Research Ethics Committee approval. |

Note that full information on the approval of the study protocol must also be provided in the manuscript.

# Field-specific reporting

Please select the one below that is the best fit for your research. If you are not sure, read the appropriate sections before making your selection.

☒ Life sciences ☐ Behavioural & social sciences ☐ Ecological, evolutionary & environmental sciences

For a reference copy of the document with all sections, see nature.com/documents/nr-reporting-summary-flat.pdf

# Life sciences study design

All studies must disclose on these points even when the disclosure is negative.

| | |
|---|---|
| Sample size | Bulk ATAC-seq: n=6. CUT&RUN: n=2. Multiome: n=4. RT-qPCR: n=10. Imaging Mass Cytometry: n= 9. |
| Data exclusions | Imaging data was only excluded if the structure of a lymphoid follicle was not present in the region of interest. |
| Replication | RT-qPCR was carried out in triplicate technical replicates. For the imaging, multiple ROI were selected for each of three biological replicates of each tissue. 9ROI from spleen, 7 ROI from appendix and 6 ROI from tonsil were analysed. |
| Randomization | All tissues studied were normal and there was no grouping other than original of tissue. |
| Blinding | It was not possible to blind this study though much of the work uses undirected analysis methods which by their nature are blinded to the researcher. |

# Behavioural & social sciences study design

All studies must disclose on these points even when the disclosure is negative.

| | |
|---|---|
| Study description | N/A |
| Research sample | N/A |
| Sampling strategy | N/A |
| Data collection | N/A |
| Timing | N/A |
| Data exclusions | N/A |
| Non-participation | N/A |
| Randomization | N/A |

# Ecological, evolutionary & environmental sciences study design

All studies must disclose on these points even when the disclosure is negative.

| | |
|---|---|
| Study description | N/A |
| Research sample | N/A |
| Sampling strategy | N/A |
| Data collection | N/A |
| Timing and spatial scale | N/A |
| Data exclusions | N/A |
| Reproducibility | N/A |
| Randomization | N/A |
| Blinding | *Describe the extent of blinding used during data acquisition and analysis. If blinding was not possible, describe why OR explain why blinding was not relevant to your study.* |

Did the study involve field work? ☐ Yes ☐ No

## Field work, collection and transport

| | |
|---|---|
| Field conditions | N/A |
| Location | N/A |
| Access & import/export | N/A |
| Disturbance | N/A |

# Reporting for specific materials, systems and methods

We require information from authors about some types of materials, experimental systems and methods used in many studies. Here, indicate whether each material, system or method listed is relevant to your study. If you are not sure if a list item applies to your research, read the appropriate section before selecting a response.

### Materials & experimental systems

| n/a | Involved in the study | |
|---|---|---|
| ☐ | ☒ | Antibodies |
| ☐ | ☐ | Eukaryotic cell lines |
| ☐ | ☐ | Palaeontology and archaeology |
| ☐ | ☐ | Animals and other organisms |
| ☐ | ☐ | Clinical data |
| ☐ | ☐ | Dual use research of concern |
| ☐ | ☐ | Plants |

### Methods

| n/a | Involved in the study | |
|---|---|---|
| ☒ | ☐ | ChIP-seq |
| ☐ | ☒ | Flow cytometry |
| ☒ | ☐ | MRI-based neuroimaging |

## Antibodies

Antibodies used

Antibodies for B cell sorting for bulk ATAC-seq and H3K4me3 - TS cells
Target Fluorophore Clone Cat. Number Supplier Dilution
CD19 APC HIB19 20-0199-T100 TONDO Bioscience 1 in 50
CD10 PE HI10a 312203 BioLegend 1 in 200
IgM BV711 MHM-88 314539 BioLegend 1 in 50

Antibodies for B cell sorting for bulk ATAC-seq and H3K4me3 - Mature cells
Target Fluorophore Clone Cat. Number Supplier Dilution

CD19 APC HIB19 20-0199-T100 TONDO Bioscience 1 in 50
CD10 PE HI10a 312203 BioLegend 1 in 200
IgD BV605 IA6-2 563313 BioLegend 1 in 100
CD27 PE-Cy7 M-T271 356411 BioLegend 1 in 50

Antibodies for CUT&RUN
Target Cat. Number Supplier
H3K4me3 Ab8580 Abcam
IgG isotype control 12-370 Millipore

Antibodies for B cell sorting for bulk qPCR - Blood and Spleen Samples
Target Fluorophore Clone Cat. Number Supplier Dilution
CD19 APC HIB19 20-0199-T100 TONDO Bioscience 1 in 50
CD10 PE HI10a 312203 BioLegend 1 in 200
IgD BV605 IA6-2 563313 BioLegend 1 in 100
CD27 PE-Cy7 M-T271 356411 BioLegend 1 in 50

Antibodies for B cell sorting for single-cell Multiome
Target Fluorophore Clone Cat. Number Supplier Dilution
CD19 PE-Dazzle SJ25C1 363031 BioLegend 1 in 50

Antibodies for B cell sorting for single-cell CITE-seq
Target Fluorophore Clone Cat. Number Supplier Dilution
CD19 PE-Dazzle HIB19 302251 BioLegend 1 in 100
CD19 PE-Dazzle SJ25C1 363031 BioLegend 1 in 100

ADT antibodies for CITE-seq experiments
Target Antibody Clone Barcode Sequence Cat. Number Dilution
CD1C TotalSeq-C0160 L161 GAGCTACTTCACTCG 331547 0.25 !g/sample
CD11C TotalSeq-C0053 S-HCL-3 TACGCCTATAACTTG 371521 0.25 !g/sample
CD45RB TotalSeq-C0844 MEM-55 AGATGGGACTCACCA 310211 0.25 !g/sample
CD10 TotalSeq-C0062 HI10a CAGCCATTCATTAGG 312233 0.25 !g/sample
CD21 TotalSeq-C0181 Bu32 AACCTAGTAGTTCGG 354923 0.25 !g/sample
CD27 TotalSeq-C0154 O323 GCACTCCTGCATGTA 302853 0.25 !g/sample
IgM TotalSeq-C0136 MHM-88 TAGCGAGCCCGTATA 314547 0.25 !g/sample
IgD TotalSeq-C0384 IA62 CAGTCTCCGTAGAGT 348245 0.25 !g/sample

Antibodies for Imaging Mass Cytometry
Metal Marker Clone Cat. Number Dilution
144Nd FITC (for HOPX_RNA probe) FIT-22 3144006C 1 in 100
146Nd CD21 EP3093 ab271855 1 in 100
148Nd CD45RB 310202 MEM-55 1 in 500
154Sm IgD EPR6146 ab236778 1 in 300
158Gd E-Cadherin 24E10 3158029D 1 in 3000
161Dy CD20 SP32 ab236434 1 in 300
164Dy CD1c OTI2F4 NBP2-46123 1 in 500
166Er BIOTIN (for PARM1_RNA probe) 409002 1D4-C5 1 in 150
168Er Ki-67 B56 3168022D 1 in 400

Validation

Antibodies were validated by agreement with manufacturers description of reactivity. For CUT&RUN data was compared with data obtained with an isotype control.

## Eukaryotic cell lines

Policy information about cell lines and Sex and Gender in Research

Cell line source(s): N/A

Authentication: N/A

Mycoplasma contamination: N/A

Commonly misidentified lines (See ICLAC register): N/A

## Palaeontology and Archaeology

| | |
|---|---|
| Specimen provenance | N/A |
| Specimen deposition | N/A |
| Dating methods | N/A |

☐ Tick this box to confirm that the raw and calibrated dates are available in the paper or in Supplementary Information.

| | |
|---|---|
| Ethics oversight | N/A |

Note that full information on the approval of the study protocol must also be provided in the manuscript.

## Animals and other research organisms

Policy information about studies involving animals; ARRIVE guidelines recommended for reporting animal research, and Sex and Gender in Research

| | |
|---|---|
| Laboratory animals | N/A |
| Wild animals | N/A |
| Reporting on sex | Human tissues were all anonymous |
| Field-collected samples | N/A |
| Ethics oversight | Human tissues studies were carried out with research ethics approval. |

Note that full information on the approval of the study protocol must also be provided in the manuscript.

## Clinical data

Policy information about clinical studies

All manuscripts should comply with the ICMJE guidelines for publication of clinical research and a completed CONSORT checklist must be included with all submissions.

| | |
|---|---|
| Clinical trial registration | N/A |
| Study protocol | N/A |
| Data collection | N/A |
| Outcomes | N/A |

## Dual use research of concern

Policy information about dual use research of concern

### Hazards

Could the accidental, deliberate or reckless misuse of agents or technologies generated in the work, or the application of information presented in the manuscript, pose a threat to:

| No | Yes | |
|---|---|---|
| ☒ | ☐ | Public health |
| ☒ | ☐ | National security |
| ☒ | ☐ | Crops and/or livestock |
| ☒ | ☐ | Ecosystems |
| ☒ | ☐ | Any other significant area |

## Experiments of concern

Does the work involve any of these experiments of concern:

| No | Yes | |
|---|---|---|
| ☒ | ☐ | Demonstrate how to render a vaccine ineffective |
| ☒ | ☐ | Confer resistance to therapeutically useful antibiotics or antiviral agents |
| ☒ | ☐ | Enhance the virulence of a pathogen or render a nonpathogen virulent |
| ☒ | ☐ | Increase transmissibility of a pathogen |
| ☒ | ☐ | Alter the host range of a pathogen |
| ☒ | ☐ | Enable evasion of diagnostic/detection modalities |
| ☒ | ☐ | Enable the weaponization of a biological agent or toxin |
| ☒ | ☐ | Any other potentially harmful combination of experiments and agents |

# Plants

| | |
|---|---|
| Seed stocks | N/A |
| Novel plant genotypes | N/A |
| Authentication | N/A |

# ChIP-seq

## Data deposition

☐ Confirm that both raw and final processed data have been deposited in a public database such as GEO.

☐ Confirm that you have deposited or provided access to graph files (e.g. BED files) for the called peaks.

| | |
|---|---|
| Data access links<br>*May remain private before publication.* | N/A |
| Files in database submission | N/A |
| Genome browser session<br>(e.g. UCSC) | N/A |

## Methodology

| | |
|---|---|
| Replicates | N/A |
| Sequencing depth | N/A |
| Antibodies | N/A |
| Peak calling parameters | N/A |
| Data quality | N/A |
| Software | N/A |

# Flow Cytometry

## Plots

Confirm that:

- [x] The axis labels state the marker and fluorochrome used (e.g. CD4-FITC).
- [x] The axis scales are clearly visible. Include numbers along axes only for bottom left plot of group (a 'group' is an analysis of identical markers).
- [x] All plots are contour plots with outliers or pseudocolor plots.
- [x] A numerical value for number of cells or percentage (with statistics) is provided.

## Methodology

| | |
|---|---|
| Sample preparation | Cryopreserved cells used for flow cytometry were thawed, washed and resuspended in RPMI-1640 and then rested at 37oC. For bulk experiments from adult human blood, B cells were isolated by magnetic-activated cell sorting (MACS) using the B cell Isolation Kit II human (Cat.N. 130-091-151), Miltenyi Biotec, Bergisch-Gladbach, Germany) prior to sorting. The gating strategy and staining protocol used for cell sorting was comparable between PBMCs and spleen cell suspensions for all samples included in the study. Cells were blocked on ice with Human TruStain FcX (Cat.N. 422302, BioLegend), diluted 1:50 in FACS buffer (1% FBS in 1xPBS) for 10 min and then stained for 40 min on ice with pre-titrated concentrations of antibodies against major B cell surface proteins (Supplementary Table 1). Cells were washed in FACS buffer and passed through a 70 µm cell strainer to generate a single cell suspension. |
| Instrument | BD FACSAria IIu SORP (BD Biosciences) cell sorter |
| Software | BD FACSDiva software (v8.0.1) |
| Cell population abundance | Cell population abundances are included in the manuscript, including the source data. |
| Gating strategy | For bulk ATAC-sequencing, CUT&RUN and RT-qPCR experiments, live single CD19+ B cells were gated as follows at the beginning of sorting: T1 cells were chosen with CD10 gates capturing top 20% of CD10 expression; remaining 80% of CD10+ cells were gated on IgM, where T2Mhi and T2Mlo gates captured the 35% of the highest and lowest IgM-expression, respectively. Mature B cell sorting was performed on CD19+CD10- cells, using gates capturing IgD+CD27+ MZB cells, IgD-CD27+ memory cells and IgD+CD27- naïve B cells (the latter when included in the experiment). B cell populations of interest were sorted into 200microliters of 100% FBS in FBS pre-precoated DNA LoBind tubes (Cat.N. 30108051, eppendorf). Cell sorting for bulk ATAC-seq experiments was performed separately for transitional or mature cell populations. |

- [x] Tick this box to confirm that a figure exemplifying the gating strategy is provided in the Supplementary Information.

# Magnetic resonance imaging

## Experimental design

| | |
|---|---|
| Design type | N/A |
| Design specifications | N/A |
| Behavioral performance measures | N/A |

## Acquisition

| | |
|---|---|
| Imaging type(s) | N/A |
| Field strength | N/A |
| Sequence & imaging parameters | N/A |
| Area of acquisition | N/A |

Diffusion MRI ☐ Used ☐ Not used

## Preprocessing

| | |
|---|---|
| Preprocessing software | N/A |
| Normalization | N/A |
| Normalization template | N/A |
| Noise and artifact removal | N/A |

Volume censoring | N/A

## Statistical modeling & inference

Model type and settings | N/A

Effect(s) tested | N/A

Specify type of analysis: ☐ Whole brain ☐ ROI-based ☐ Both

Statistic type for inference | N/A

(See Eklund et al. 2016)

Correction | N/A

## Models & analysis

| n/a | Involved in the study |
|---|---|
| ☒ | ☐ Functional and/or effective connectivity |
| ☒ | ☐ Graph analysis |
| ☒ | ☐ Multivariate modeling or predictive analysis |

Functional and/or effective connectivity | N/A

Graph analysis | N/A

Multivariate modeling and predictive analysis | N/A
