# Supplementary material for: Epigenetic signatures mark early peripheral human B lineage bifurcation and differential transcriptional profiles in mature populations
Source: Nat Commun. 2026 Jul 14;17:8628. doi: 10.1038/s41467-026-75364-3 (PMC13487194; doi:10.1038/s41467-026-75364-3)
Supplement: Supplementary file 2 [file 41467_2026_75364_MOESM18_ESM.pdf]

nature portfolio

Peer Review File

Epigenetic signatures mark early peripheral human B lineage bifurcation and differential transcriptional profiles in mature populations

Corresponding Author: Professor Jo Spencer

This file contains all reviewer reports in order by version, followed by all author rebuttals in order by version.

Version 0:

Reviewer comments:

Reviewer #1

(Remarks to the Author)
The manuscript by Dionisi et al. combines transcriptomics and chromatin accessibility analyses to characterize a point of divergence at the transitional 2 stage between the human marginal zone and the follicular B cell fates. They identify two epigenetic signatures, consistent from the T2 to the memory subpopulations, and associate them to the expression of specific genes in the mature cells.
The study convincingly identifies the epigenic differences underlying the bifurcation between the marginal zone and follicular developmental pathways in human B cells. The analysis of the omics data is coherent and insightful, and the validation of the gene expression in the tissues strengthens the findings.
I suggest the following modifications:
- The study involves many different human B cell populations, and the authors need to improve their nomenclature and be more specific about how they define these populations. For example, it would be clearer to call the “naïve cells” naïve follicular B cells to oppose them to the “naïve MZP”. Similarly canonical memory, instead of just “memory”, would be more explicit. Are the “Ts naïve” transitional T3? There is no definition of many of the populations, and some names, even if they can be found in the literature, are not explained (IgM only, aNAV, DN2…). It makes it very challenging for the non-specialists to understand fully the data.
- In figure 1, a MDS plot would allow to appreciate the proximity between the populations.
- In figure 2, could the authors measure the mutation rates of the different populations to assess their antigen-experienced status ?
- In figure 4ab, could the correlation between chromatin accessibility at TSS and gene expression be quantified to demonstrate the differences between precursors and mature cells in this regard?
- In figure 4, could you define signatures of T2Mlo and T2Mhi and test them on MZB and canonical memory cells?
- The conclusions of figure 4g do not seem supported by the data. The transcription factor activity scores appear to be more correlated to the stage of maturation than the developmental pathway.

Reviewer #2

(Remarks to the Author)
The authors present a multi-omic study of developing and transitional B cells in the human. The goal was to better understand the molecular underpinnings on some emergent B cell populations and contextualize them back to locations in situ. Generally, the different omic analyses performed seem to be of good quality (though the data availability statements need to be added to the manuscript with DOI linked datasets). However, the underpinning premise of the first ½ of the manuscript seems to be based on staining and cell sorting patterns that we do not find credible (see below). Unless there is some significant misunderstanding on our part, we don’t believe the manuscript is publishable in the current form.

- The CD10 staining in peripheral B cells shown in Figure 1A is suspicious. 20% CD10 positivity on CD19+ cells is not normal in “adult human peripheral blood” (based on our own work profiling 100s of healthy samples, and what is widely accepted in the literature). This would be unusually high even if it was bone marrow or cord blood or in the situation of someone undergoing hematopoietic cell reconstitution. A hematopathologist colleague we enquired with regarding this

stated that if a sample in the clinical flow lab looked like this, they would be working up a diagnosis for leukemia or lymphoma. Further inspection of the flow cytometry panel shows multiple B cell markers (IgD, CD27..ect) that have fluorophores with potentially overlapping profiles with CD10 on PE here – perhaps this is the issue? We would suggest the authors look deeply at this to understand potential error and better explain the nature of the staining patterns they sorted the cells on (i.e., leave one channel out controls, use of true positive CD10 cells like REH or NALM6 cells). Finally, this is also inconsistent with the foundational reference (Ref 3) that the authors provide to establish the existence of these cells. They are exceedingly rare in this report in peripheral blood – not 20% of the population.

- The cytometry data / gating scheme for the sorts is also insufficient. All gates / cell events need to be shown. Control data? Purity controls? The data in the figures has been processed further than what has been disclosed.

- Paper narrative – the Summary and Introduction are more editorial about the problem and don't contextualize the 'solution this study brings. The authors should really lay down more specific high-level claims – B cell branching points – what they did - how are they identified / defined / refined them?

Reviewer #3

(Remarks to the Author)
Summary

Dionisi et al present multiomics analysis of adult and fetal human B cells at pseudobulk and single-cell resolution to study the epigenetic basis of B cell lineage specification. The manuscript primarily focuses on transitional B cell stages and the genome-wide differences between T2 B cells conventionally delineated by surface IgM levels (T2Mlo vs T2Mhi). The study design is reasonable, and the evidence for prenatal epigenomic programming of B cell lineage specification is interesting. However, the key claim pertaining to poised chromatin vs active transcription between B cell developmental stages requires additional empirical support from further experiments described below (independent validation and controls). Additional analyses of the presented data (high-level summaries of shared vs. differential accessibility, ontology or gene set enrichment analyses) are also needed. Comparison of the presented data from healthy donors with publicly available B cell scRNA-seq datasets that include transitional B cell phenotypes from diseases is optional but would broaden the study impact.

Major

1. Additional expression analyses are needed to support the key claim of genes poised for transcription in transitional B cells vs actively transcribed in differentiated B cell states. The single-cell multiome data are consistent with the conclusion; however, both scRNA-seq and scATAC-seq are inherently sparse assays prone to undersampling artifacts (specifically, false negatives due to read dropout). Thus, single-cell sequencing data alone cannot definitively support determination of poised vs. actively transcribed genes – especially for genes expressed at low levels (an absence of evidence vs. evidence of absence problem). Ideally, the authors should assay subset-delineating genes (e.g., PARM1, HOPX, NT5E, PDE4D for T2Mlo; CYTH1, JAM1, SLC9A1 for T2Mhi) in sorted B cell populations (T1, T2Mlo, T2Mhi) with RT-PCR. Confirming the absence of expressed transcripts from gene loci with open chromatin using an orthogonal experimental method will substantially strengthen the evidence for early poised chromatin. On this point – the spatial analyses presented in figure 5 are useful. However, there appears to be substantial variation in PARM1 and HOPX between B cell subsets across the different analyzed tissues (tonsil, spleen, appendix). Elevated expression in naïve vs MZB cells is clear in the spleen but is debatable (or even reversed) in the appendix and tonsil tissues. Here again, a simple RT-PCR analysis of sorted B cell subsets is important. While cell segmentation from spatial imaging makes sense, analyses of disaggregated cells would likely be cleaner to prove the point.

2. The activating mark H3K4me3 alone is not sufficient to formally define poised chromatin. The authors need to perform at least two additional CUT&RUN (or ChIP-seq) experiments on sorted B cell populations: 1) an assay of H3K27me3, which will support the identification of poised regions, and 2) an Ig isotype control to account for non-specific signals. The Ig experiment is especially important – while the CUT&RUN assay has lower background than ChIP-seq, the modest H3K4me3 intensities shown for some genes of interest underscores the need for presentation alongside control tracks. Analysis of H3K27ac would also be useful, however this is not strictly necessary to support the authors' claim.

3. A relatively small number of individual genes are investigated to distinguish T2Mlo vs T2Mhi B cells, but a global view of functional biological differences between these clusters is currently lacking. The study would benefit greatly from high-level ontology or enrichment analyses of differentially expressed (or differentially accessible) genes between the delineated cell phenotypes. Such analysis may reveal previously unknown aspects of biological changes through B cell development.

Minor

1. The authors should include a global summary of overlapping DARs among T1, T2Mlo, T2Mhi, etc. subsets as a Venn diagram or table. This is a simple but important analysis that will establish high-level epigenomic similarities and differences before jumping into the comparative studies of a much smaller number of genes. What percent of accessible regions (# of called peaks or overlapping peak intensities) are common or variable across the defined B cell clusters?

2. The gating strategy used to isolate MZB cells (CD19+CD10-IgD+CD27+) for pseudobulk ATAC-seq experiment may contain several B cell subsets with distinct antigen experience histories and epigenomic programming. The single-cell ATAC data are not prone to this issue, however the authors should acknowledge this possibility / limitation in interpreting pseudobulk accessibility in the text.

3. The T2Mmid scMultiome cluster was excluded from many analyses. The provided rationale for excluding this state is that it is “consistent with the approach used for B cell sorting in bulk ATAC-seq analysis and in other studies in recognition of indistinct boundaries between T2Mhi and T2Mlo cells.” (p6, line 175). This reviewer feels that the T2Mmid cells should be included precisely because they appear to provide phenotypic continuity between states that have been conventionally defined on the basis of limited biomarkers. This is arguably the whole point of transcriptome-wide single-cell analysis – to discover or define cell states and identities that are not captured by low-dimensional techniques. What DARs and DEGs define T2Mmid versus T2Mhi and T2Mlo? Specifically, is T2Mmid clearly a continuum between the terminal T2M phenotypes, or are there genomic features that are unique to T2Mmid versus either T2Mhi or T2Mlo? Based on Figure 2G-H, T2Mmid it would be interesting to know whether T2Mmid pseudotemporally precedes both T2Mlo and T2Mhi or only one of these. It seems that T2Mhi may directly develops from T1 without passing through T2Mmid?

4. While not essential to support the claims in the manuscript, the authors are encouraged to explore intra-subset chromatin accessibility between fetal and adult cells. For example, to what extent are accessible chromatin regions conserved between cord blood and adult T2Mlo B cells? There appear to be enough donor replicates for adult and cord blood pseudobulk ATAC-seq to identify conserved accessibility features associated with age / developmental stage across donors. This analysis would be interesting and may provide additional nuance to the timing of B cell epigenetic identity.

5. The authors present single-cell sequencing data from healthy donor B cells and briefly discuss possible perturbations to transitional B cell populations in disease (specifically, SLE) – these could be changes in B subset frequency and/or transcriptomic changes within a given subset between health and disease. There are ample publicly available peripheral B cell scRNA-seq from autoimmune and chronic infectious diseases that the authors could analyze without additional wet lab experiments to explore this possibility. Here is a non-exhaustive list of potentially relevant NIH Gene Expression Omnibus accessions: SLE (GSE135779), multiple sclerosis (GSE1330028, GSE138266, GSE267750), and chronic infections (HIV, Malaria: GSE149729). Again, this analysis is not strictly necessary to support the manuscript claims, but such comparative analysis is well within reach and would substantially enhance the study’s impact.

6. The manuscript is well-written overall; however, the introduction would benefit from improved continuity for introducing B cell lineages and nomenclature. For example, please clearly frame the relation of T2M cell subsets and splenic MZB cells between the 2nd and 3rd paragraphs. The T2Mlo and T2Mhi designations are introduced early but not mentioned after the 2nd paragraph of the introduction.

7. Page 3, line 97: the details are provided in the methods section, but please specify the source of the B cells used for sorting here as well.

8. Page 12, line 402: the Miltenyi B cell enrichment kit product number is missing a digit (130-091-151)

Inquiries

1. Page 4, line 102: the authors state: “We observed that T1, T2Mhi , and T2Mlo cells were different epigenetically, with transposase-accessible regions identified for each population being consistent between 3 donors” – Quantitatively, how much conservation was observed across donors (see comment on the need for global accessibility analysis)? Can the authors provide additional detail on the age and sex differences for donor-derived B cells?

2. Page 4, line 109: please re-phrase or define what is meant by “functional genes” in this context – protein-coding?

3. Page 4, line 120: the authors use the phrase “post-transcriptional histone modifications” – please correct or clarify. H3K4me3 is a post-translational modification – alternatively, are the authors referring to an implied temporal difference in transcriptional control regulated by H3K4me3?

4. The authors propose that the T2M subset split is epigenetically predefined from birth yet modifiable as indicated in some autoimmune diseases. Can they speak to cause versus effect on this point? Specifically, is there evidence of an early life difference in epigenetic equilibrium specifying development along one branch versus another that associated with disease risk? Alternatively, are there (epi)genetic predispositions for shifts in B cell development in response to environmental factors that could be discussed?

Version 1:

Reviewer comments:

Reviewer #1

(Remarks to the Author)

The modifications and additions in response to the reviews of this manuscript address adequately the questions raised and further support the conclusions of this study. I have no further comments.

Reviewer #3

(Remarks to the Author)
Please see the attached document for a discussion of CD10 transitional B cell frequencies. In general, I believe the results for CD10+ cells presented in the revised manuscript are justifiable.

Author responses to Reviewer #3 comments

Major Comment #1
I thank the authors for the new experiments they performed and show in Figure 4G. Please also include the note in the new text section that these genes were undetectable after 45 RT-PCR cycles and/or provide this negative result in a supplementary figure – this is the critical component of the poised-vs-expressed questions at hand.

The authors have substantively addressed other major comments and most minor comments through new experiments, inclusion of important control data, and text revisions.

Open Access This Peer Review File is licensed under a Creative Commons Attribution 4.0 International License, which permits use, sharing, adaptation, distribution and reproduction in any medium or format, as long as you give appropriate credit to the original author(s) and the source, provide a link to the Creative Commons license, and indicate if changes were made.
In cases where reviewers are anonymous, credit should be given to 'Anonymous Referee' and the source.
The images or other third party material in this Peer Review File are included in the article's Creative Commons license, unless indicated otherwise in a credit line to the material. If material is not included in the article's Creative Commons license and your intended use is not permitted by statutory regulation or exceeds the permitted use, you will need to obtain permission directly from the copyright holder.
To view a copy of this license, visit https://creativecommons.org/licenses/by/4.0/

Responses to reviewers' comments

We are grateful to the reviewers for the time spent on our work and for their helpful comments. The reviewers' comments below are in grey italics and our responses are in black plain font.

Reviewer #1
The modifications and additions in response to the reviews of this manuscript address adequately the questions raised and further support the conclusions of this study. I have no further comments.

We thank the reviewer for their positive assessment of our work.

Reviewer #3 (Remarks to the Author):
Please see the attached document for a discussion of CD10 transitional B cell frequencies. In general, I believe the results for CD10+ cells presented in the revised manuscript are justifiable.

We are grateful to the reviewer for time spent considering this issue, and for their positive overall assessment.

CD10+ B cell frequencies
I agree with Reviewer #2 that 20% of B cells being CD10+ would be a cause for concern, though the odds that 5 anonymous donors would all have malignancy resulting in high CD10+ fractions is astronomically low. That suggests (but does not guarantee) technical rather than biological explanations for the higher than expected frequency. Based on the authors' response, this appears to have been due in part to a figure annotation error that has been corrected in the revised manuscript. Figure 1A now shows ~12% CD10+ frequency within the peripheral B cell pool.

We apologise for the mislabelling of the flow plot in the first submission. This was copied by mistake from another plot.

This frequency is still higher than the expected 3% frequency cited as normal by Reviewer #2. Based on my own brief review of relevant literature, Reviewer #2 is correct that 3% is a normal CD10+ B cell frequency in healthy adults. While the precise composition of the B cell compartment is not my direct area of expertise, two explanations for the still elevated observed frequency seem most plausible (see below).

Whilst we acknowledge that average transitional B cell frequency of 3% of CD19 has been observed, this is very subjective. The values we observe are within the ranges observed in many other studies including:

https://pubmed.ncbi.nlm.nih.gov/19965666/
https://pubmed.ncbi.nlm.nih.gov/26525227/
https://pubmed.ncbi.nlm.nih.gov/27780620/
https://pubmed.ncbi.nlm.nih.gov/30018075/
https://www.mdpi.com/1422-0067/26/19/9539/
https://pubmed.ncbi.nlm.nih.gov/41540267/

Possibility #1: immune status / recent infection history of anonymous donors

12% CD10+ B cell frequency may still be considered normal / healthy in the context of convalescence after infection due to immune reconstitution. The transitional B cell compartment can be temporarily expanded for extended periods (weeks to months) following

infection by numerous viruses, including those with high prevalence. In contrast to the malignancy hypothesis, receiving blood from donors who had experienced viral infections weeks to months prior to blood donation seem statistically plausible. For example, the linked publication shows B cell compartment composition in the months following SARS-CoV-2 infection: https://www.cell.com/cell/fulltext/S0092-8674(21)00093-3?elqTrackId=73de71bf0319482899d7d15d53e960e0. Figure 2B demonstrates an expansion from ~3-5% Naïve/transitional B cells at 0 months post-infection to ~12.5% Naïve/transitional B cells at 6 months post-infection (depicted in dark green bar). While the fraction of naïve cells in this mixed group is not specified, it indicates an expansion post-infection. Moreover, it is useful to note the frequency of the Transitional B cell cluster (distinguished from naïve cells) apparent from the UMAP in 2A is also roughly consistent with the frequencies found by the authors in the submitted manuscript.

Thank you for bringing this interesting paper to our attention. This is a study of memory responses to SARS-CoV-2. The first step in their sorting strategy before acquiring data that is analysed in Figure 2 is to sort cells not expressing IgD. Since most transitional cells express IgD, this would exclude most transitional B cells from the data in Figure 2. The populations in the Figure the reviewer refers to are therefore not analogous to the populations we study.

A separate study of individuals recently infected with HIV demonstrates a substantial increase in transitional B cell frequency (median ~15% of peripheral B cell pool; see Figure 1B): https://www.cell.com/cellreports/ fulltext/S2211-1247(21)00720-8

In this interesting paper, a population of CD27-CD38+ cells is investigated that increases in frequency in HIV. These cells are then investigated further and shown to have features of transitional B cells. However, CD27-CD38+ is not a conventional way of sorting the whole transitional cell population, so again, we think that whilst this is an interesting piece of work this is not giving an accurate comparison with our data. If transitional cells are gated using CD38, this usually involves analysis of co-expression of CD24 that enables visualization of the point at which CD38 and CD24 expression exceed the levels seen on naïve B cells. We therefore found comparison of our data with this data challenging.

In this very specific respect, immune system recovery after infection may resemble a similar effect observed in immunocompromised contexts: https://academic.oup.com/jimmunol/article-abstract/176/3/1506/8059613

This interesting study describes an increase in transitional B cells in immunocompromised individuals. Although the cells we studied were from anonymous donors and details relating to the donors were not available, the system that collects these cells from blood donors would exclude those who had known conditions including immunodeficiencies. We do not therefore think this could impact our data.

While immune system recovery weeks to months after an infection may explain the result, the authors would not be able to test this possibility due to the anonymous nature of the donors. This possibility could be noted in the discussion regarding the CD10+ B cell frequency, but it would remain speculative.

We are grateful for this thought. However, as the reviewer acknowledges, this is not something we could test and the field is complex. We were able to find papers describing both increased and decreased frequencies of transitional B cells in infection and therefore felt that it would be too much of a distraction from the main theme of the paper to discuss this here in a balanced way. URLs of examples are included below and for interest we have also included the approximate range of transitional B cell frequencies in healthy controls

observed in these studies extracted from the Figures, that are consistent with the frequencies of transitional B cells that we sort.

Increased TS in infection:
https://pubmed.ncbi.nlm.nih.gov/33519823/
(range of transitional B cells as % of B cells approximately 1-14.5%)

https://pubmed.ncbi.nlm.nih.gov/20656924/
(range of transitional B cells in health as %CD19 approximately 1-60%)

Decreased TS in infection:
https://www.mdpi.com/1467-3045/47/4/245
(range of transitional B cells in health as %CD19 approximately 0-18%)

https://pubmed.ncbi.nlm.nih.gov/31736948/
(range of transitional B cells in health as %CD19 approximately 0-22%)

https://pubmed.ncbi.nlm.nih.gov/36357845/
(range of transitional B cells in health as %CD19 approximately 1-17%)

Possibility #2: flow gating cutoffs
I agree with the authors' response to Reviewer #2 that the flow panel is appropriate. The fluorophores are well-separated and the emission bandpass filters on the cytometer would minimize spectral overlap (possibly even without matrix compensation).

Thank you for this comment. We were very careful with our panel design.

The higher-than-expected CD10+ fraction may simply redound to the chosen gate. While it skews toward permissivity instead of stringency in calling CD10 positivity, the gate the authors use in Figure 1A seems justifiable given the subsequent conservative gating of CD10-high signal to sort T1 cells and further gating by IgM status to delineate T2 subsets.

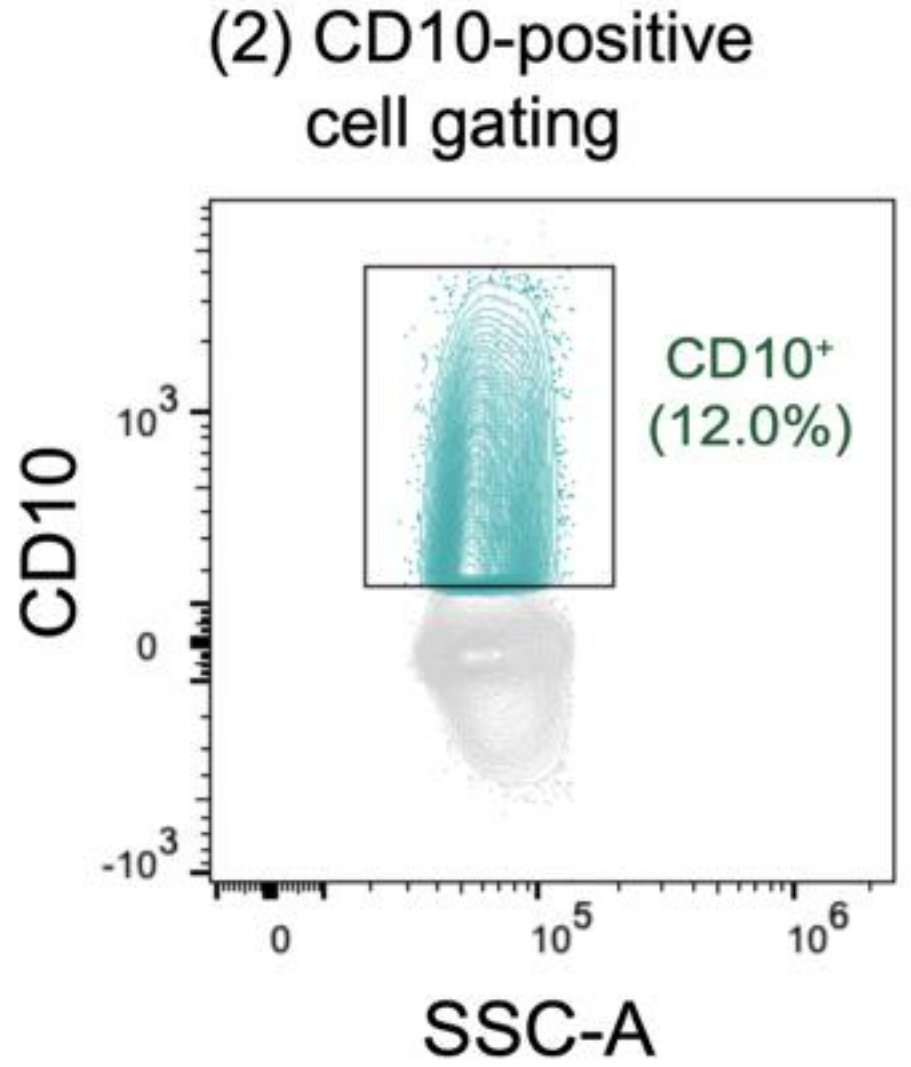

A stricter CD10 lower threshold (e.g., 500 or 1000 instead of 100 counts) could easily lead to a conclusion of 3-5% CD10+ fraction of peripheral B cells. Discretizing continuous staining distributions inherently introduces a degree of subjectivity that affects cell population quantification. The transitional cells are, by nature, part of a phenotypic continuum. Called gates are inescapably arbitrary to varying degrees (e.g., see Fig. 5 here: https://ashpublications.org/blood/article/105/11/4390/19512/Identificationand-

characterization-of-circulating?guestAccessKey=). This warrants the subsequent use and focus of the study on genome-wide single-cell analyses.

Thank you for this comment and for acknowledging our use of IgM expression as an additional check, so that the percentage of transitional cells is less than the total CD10 expressing cells. We agree that setting gates for transitional B cells, especially their subsets, is challenging. We have therefore now mentioned in the results, Methods and Discussion, problems associated with the subjectivity of gating cells that lie on a continuum of expression.

Given the concordance between flow phenotype composition and single-cell gene expression, this issue seems resolved by the authors' error correction, address of numerous other studies, and the technical impact of gate definitions on quantitation. The fact that multiple gene indicated from sorted fraction ATAC-seq experiments are validated by the pan-B single-cell expression data generally appears to vindicate the defined gating.

We appreciate this view, thank you. Multiple methodologies were used in our study to cross reference between datasets to ensure that outcomes are mutually supportive.

Author responses to Reviewer #3 comments
Major Comment #1
I thank the authors for the new experiments they performed and show in Figure 4G. Please also include the note in the new text section that these genes were undetectable after 45 RTPCR cycles and/or provide this negative result in a supplementary figure – this is the critical component of the poised-vs-expressed questions at hand.

Thank you for this comment. We have now included the text "These genes were undetectable after 45 cycles of RT-qPCR analysis of sorted transitional B cell subsets".

The authors have substantively addressed other major comments and most minor comments through new experiments, inclusion of important control data, and text revisions.

We thank the reviewer for this positive response to our revisions.
