# Supplementary material for: Epigenetic signatures mark early peripheral human B lineage bifurcation and differential transcriptional profiles in mature populations
Source: Nat Commun. 2026 Jul 14;17:8628. doi: 10.1038/s41467-026-75364-3 (PMC13487194; doi:10.1038/s41467-026-75364-3)
Supplement: Supplementary file 3 [file 41467_2026_75364_MOESM1_ESM.pdf]

Epigenetic signatures mark early peripheral human B lineage bifurcation and differential transcriptional profiles in mature populations

Dionisi C. et al.

Corresponding Author: Spencer J.

Supplementary Information

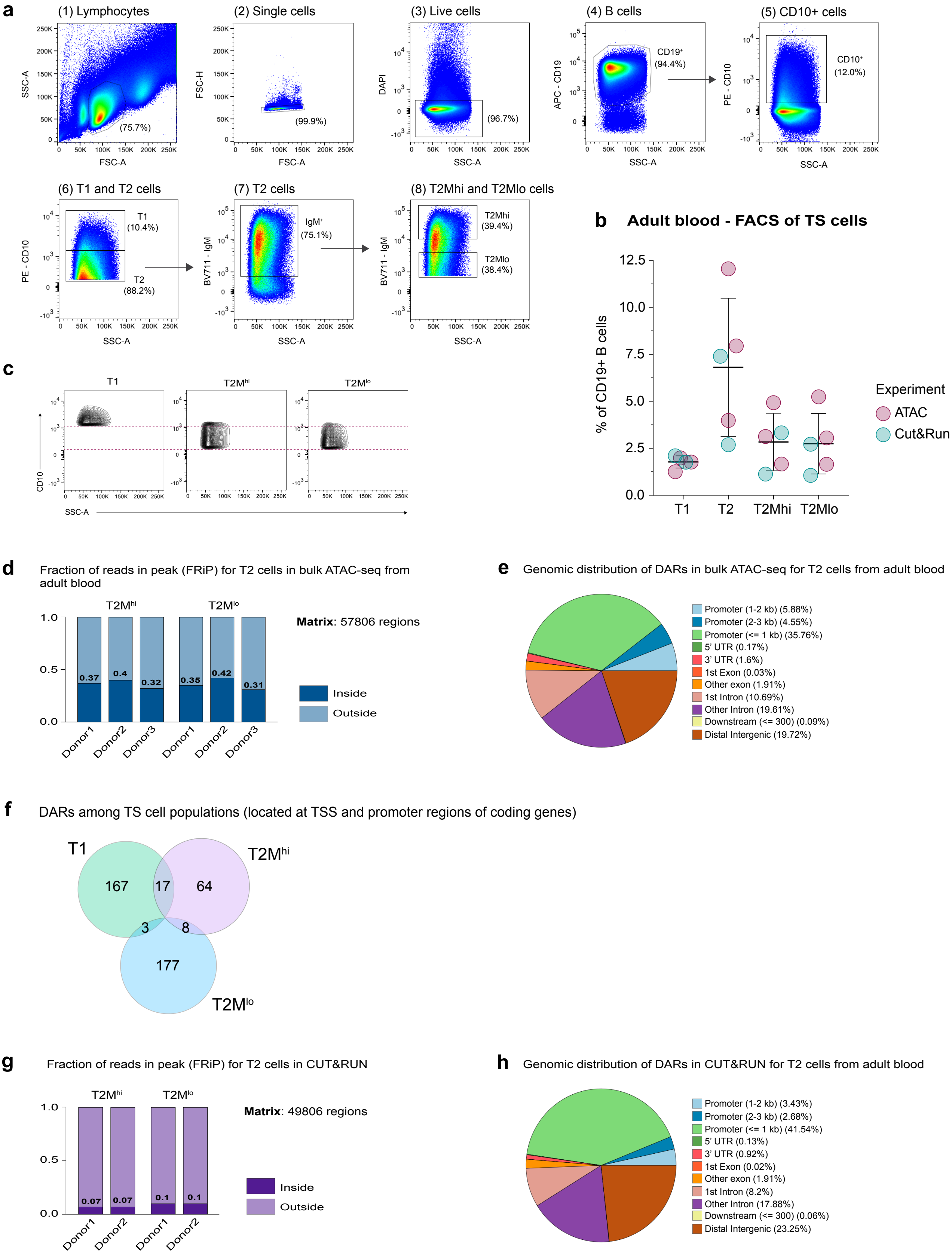
a
(1) Lymphocytes
SSC-A
FSC-A
(75.7%)
(2) Single cells
FSC-H
FSC-A
(99.9%)
(3) Live cells
DAPI
SSC-A
(96.7%)
(4) B cells
APC - CD19
SSC-A
CD19⁺
(94.4%)
(5) CD10+ cells
PE - CD10
SSC-A
CD10⁺
(12.0%)
(6) T1 and T2 cells
PE - CD10
SSC-A
T1
(10.4%)
T2
(88.2%)
(7) T2 cells
BV711 - IgM
SSC-A
IgM⁺
(75.1%)
(8) T2Mhi and T2Mlo cells
BV711 - IgM
SSC-A
T2Mhi
(39.4%)
T2Mlo
(38.4%)
b
Adult blood - FACS of TS cells
% of CD19+ B cells
T1
T2
T2Mhi
T2Mlo
Experiment
ATAC
Cut&Run
c
T1
T2Mʰⁱ
T2Mˡᵒ
CD10
SSC-A
d
Fraction of reads in peak (FRiP) for T2 cells in bulk ATAC-seq from adult blood
T2Mʰⁱ
T2Mˡᵒ
0.37
0.4
0.32
0.35
0.42
0.31
Donor1
Donor2
Donor3
Donor1
Donor2
Donor3
Matrix: 57806 regions
Inside
Outside
e
Genomic distribution of DARs in bulk ATAC-seq for T2 cells from adult blood
Promoter (1-2 kb) (5.88%)
Promoter (2-3 kb) (4.55%)
Promoter (<= 1 kb) (35.76%)
5' UTR (0.17%)
3' UTR (1.6%)
1st Exon (0.03%)
Other exon (1.91%)
1st Intron (10.69%)
Other Intron (19.61%)
Downstream (<= 300) (0.09%)
Distal Intergenic (19.72%)
f
DARs among TS cell populations (located at TSS and promoter regions of coding genes)
T1
T2Mʰⁱ
T2Mˡᵒ
167
17
64
3
8
177
g
Fraction of reads in peak (FRiP) for T2 cells in CUT&RUN
T2Mʰⁱ
T2Mˡᵒ
0.07
0.07
0.1
0.1
Donor1
Donor2
Donor1
Donor2
Matrix: 49806 regions
Inside
Outside
h
Genomic distribution of DARs in CUT&RUN for T2 cells from adult blood
Promoter (1-2 kb) (3.43%)
Promoter (2-3 kb) (2.68%)
Promoter (<= 1 kb) (41.54%)
5' UTR (0.13%)
3' UTR (0.92%)
1st Exon (0.02%)
Other exon (1.91%)
1st Intron (8.2%)
Other Intron (17.88%)
Downstream (<= 300) (0.06%)
Distal Intergenic (23.25%)

Supplementary Figure 1. Bulk ATAC-seq and H3K4me3 CUT&RUN analysis of transitional B cells from adult human PBMCs.

(a) Representative B cell sorting strategy for transitional B cell subsets from MACS-sorted adult human peripheral blood mononuclear cells (PBMCs) in bulk ATAC-seq (n=3 biological replicates) and H3K4me3 CUT&RUN (n=2 biological replicates) experiments. Single live cells were selected based on negative DAPI staining. B cells were selected based on $CD19^+$ expression, allowing the exclusion of $CD19^-$ cells persisting after MACS sorting. At the beginning of sorting, gates were set to capture the cells with the top 20% of CD10 expression within CD10 expressing cells. These were designated T1. Then gates were set to capture the top 35% and bottom 35% of IgM expression in the remaining $CD10^+IgM^+$ cells. These were designated $T2M^{hi}$ and $T2M^{lo}$ respectively. Percentages of cells at the end of sorting from a representative biological replicate are indicated. Source data is provided as a Source Data file.

(b) Combined dot plot of final percentages of T2 gated and T1, $T2M^{hi}$ and $T2M^{lo}$ gated and sorted $CD19^+$ B cells from adult human PBMCs in bulk ATAC-seq and H3K4me3 CUT&RUN experiments (n=5). Error bars represent mean ± SD.

(c) Representative plot of CD10 expression in distinct transitional B cell subpopulations as assessed during fluorescence-activated cell sorting for bulk ATAC-seq experiments.

(d) Matrix size and FriP for each of the individual bulk ATAC-seq libraries for T2 cells from adult blood.

(e) Proportions of DARs in adult blood T2 cells mapping to distinct genomic regions in bulk ATAC-seq.

(f) Venn Diagram of statistically significant DARs (p-val < 0.05) between T1, $T2M^{hi}$ and $T2M^{lo}$ cells located at the TSS or promoter region of genes.

(g) Matrix size and FriP for each of the individual bulk H3K4me3 CUT&RUN libraries for T2 cells from adult blood.

(h) Proportions of DARs in adult blood T2 cells mapping to distinct genomic regions in bulk H3K4me3 CUT&RUN.

DAR: differentially accessible region; UTR: untranslated region; TSS: transcriptional start site; FriP: fraction of reads in peaks; T1: transitional 1; T2: transitional 2; $T2M^{hi}$: transitional IgM^{high} T2 cell; $T2M^{lo}$: transitional IgM^{low} T2 cell.

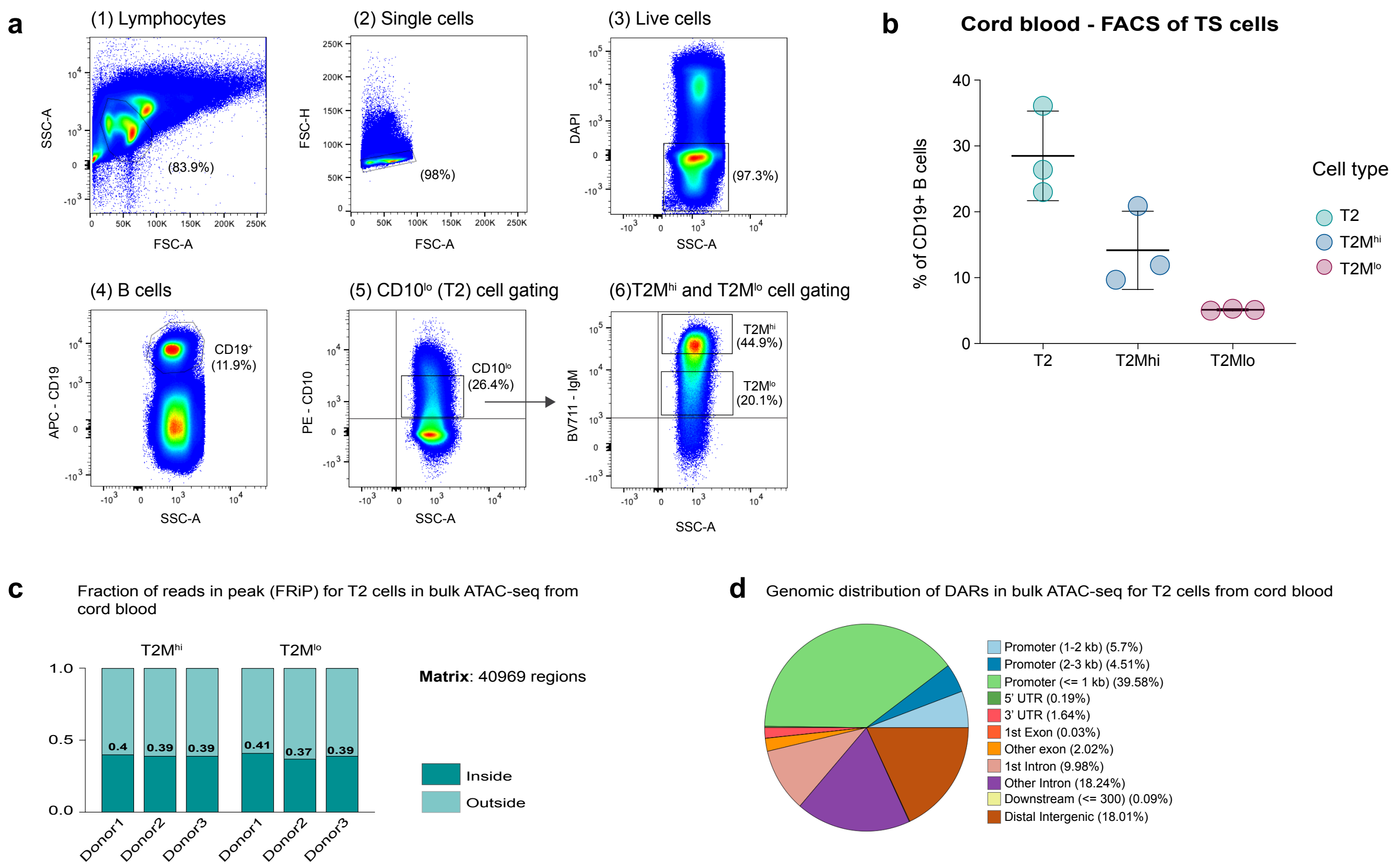

Supplementary Figure 2. Bulk ATAC-seq analysis of transitional B cells from cord human PBMCs.

(a) Representative B cell sorting strategy for transitional B cell subsets from MACS-sorted cord human PBMCs in bulk ATAC-seq experiments (n=3 biological replicates). Single live cells were selected based on negative DAPI staining. B cells were selected based on CD19^{+} expression. At the beginning of sorting, gates were set to capture the cells with bottom 80% of CD10 expression. Then gates were set to capture the top 35% and bottom 35% of IgM expression within this gate. These were designated T2M^{hi} and T2M^{lo} respectively. Percentages of cells at the end of sorting from a representative biological replicate are indicated. Source data is provided as a Source Data file.

(b) Dot plot of final percentages of T2 gated and T2M^{hi} and T2M^{lo} gated and sorted CD19^{+} B cells from cord human PBMCs in bulk ATAC-seq experiments (n=3). Error bars are mean ± SD.

(c) Matrix size and FriP for each of the individual bulk ATAC-seq libraries for T2 cells from cord blood.

(d) Proportions of DARs in cord blood T2 cells mapping to distinct genomic regions in bulk ATAC-seq.

PBMC: peripheral blood mononuclear cell; DAR: differentially accessible region; UTR: untranslated region; TSS: transcriptional start site; FriP: fraction of reads in peaks; T1: transitional 1; T2M^{hi}: transitional IgM^{high} T2 cell; T2M^{lo}: transitional IgM^{low} T2 cell.

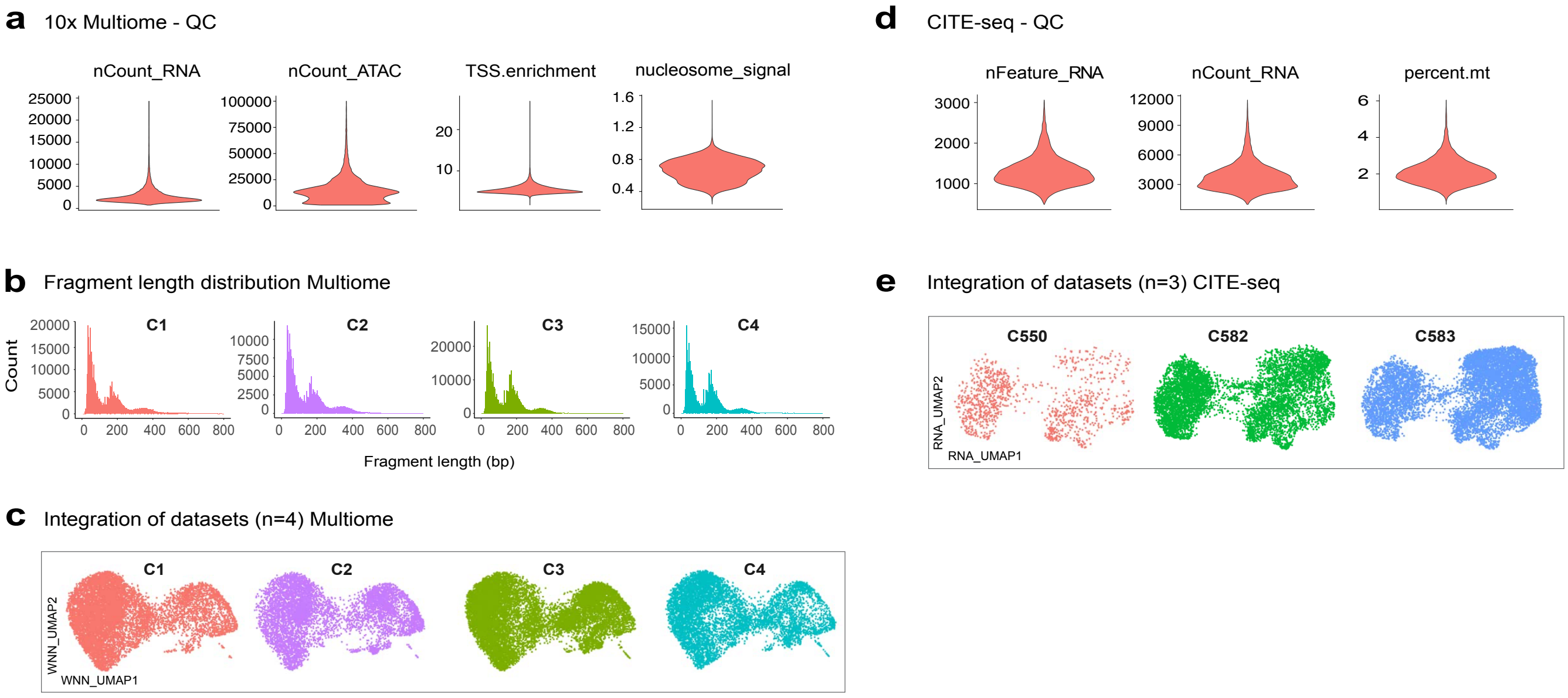

Supplementary Figure 3. QC and data integration in Multiome and CITE-seq datasets.

(**a**) QC steps of Multiome dataset for gene expression and chromatin accessibility. Low quality cells with low or high RNA and ATAC counts, or with low TSS-enrichment and nucleosome-signal scores were removed (n=4).

(b) Consistent high-quality fragment length patterns across biological replicates, corresponding to nucleosome-free (< 100 bp), mono- (100-200 bp), di-nucleosome (200-400 bp) or longer (> 400 bp) chromatin regions.

(c) Efficient integration of the 4 datasets (biological replicates C1-4) included in the Multiome experiment.

(d) Quality control steps of CITE-seq dataset. Low quality cells with a low or high number of genes (nFeature_RNA) and RNA counts, or with high percentage of mitochondrial DNA were removed (n=3).

(e) Efficient integration of the 3 datasets (biological replicates C550, C582, C583) included in the CITE-seq experiment.

QC: quality control; TSS: transcriptional start site.

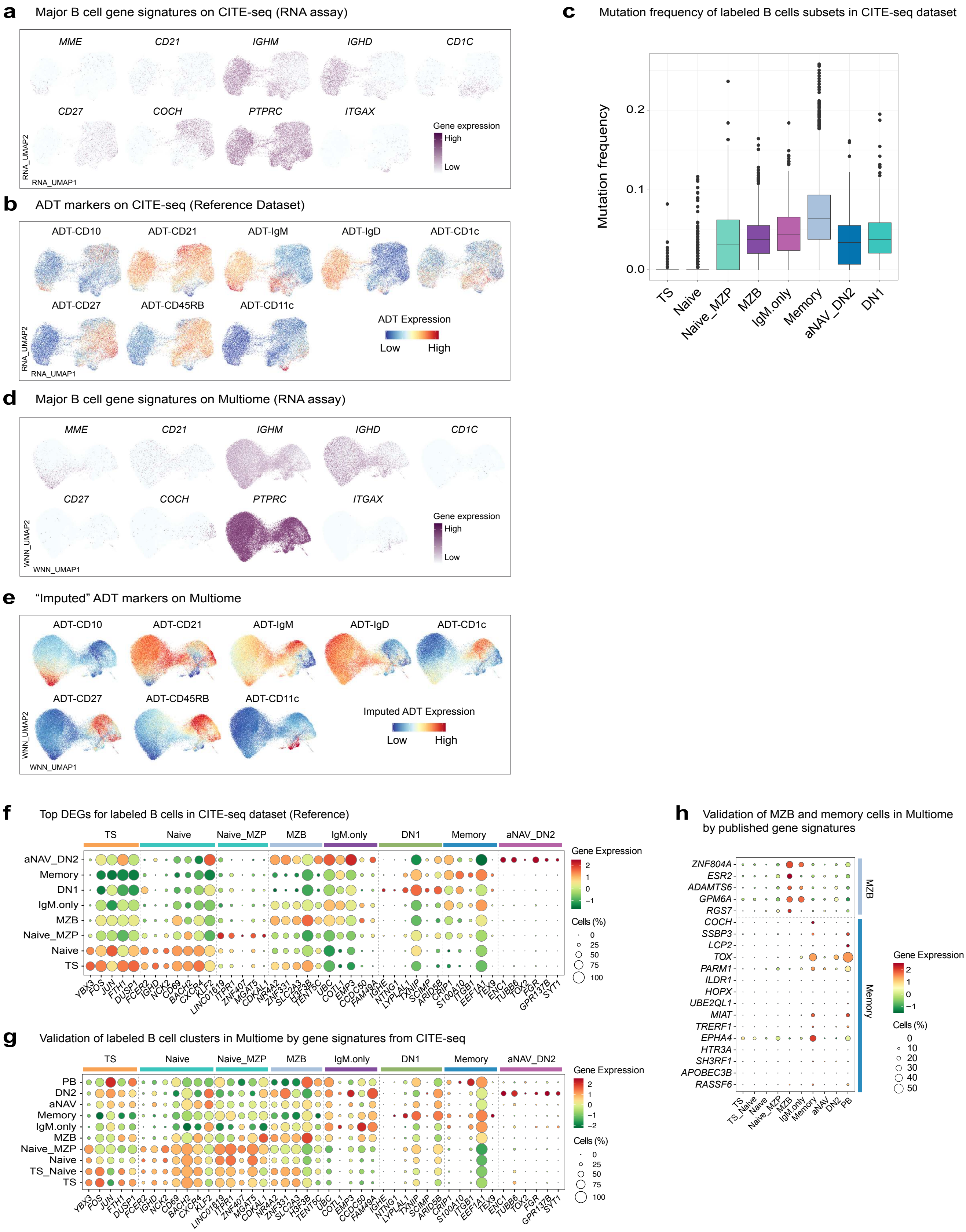

a
Major B cell gene signatures on CITE-seq (RNA assay)
MME
CD21
IGHM
IGHD
CD1C
CD27
COCH
PTPRC
ITGAX
Gene expression
High
Low
RNA_UMAP2
RNA_UMAP1
b
ADT markers on CITE-seq (Reference Dataset)
ADT-CD10
ADT-CD21
ADT-IgM
ADT-IgD
ADT-CD1c
ADT-CD27
ADT-CD45RB
ADT-CD11c
ADT Expression
Low
High
RNA_UMAP2
RNA_UMAP1
c
Mutation frequency of labeled B cells subsets in CITE-seq dataset
Mutation frequency
0.0
0.1
0.2
TS
Naive
Naive_MZP
MZB
IgM.only
Memory
aNAV_DN2
DN1
d
Major B cell gene signatures on Multiome (RNA assay)
MME
CD21
IGHM
IGHD
CD1C
CD27
COCH
PTPRC
ITGAX
Gene expression
High
Low
WNN_UMAP2
WNN_UMAP1
e
"Imputed" ADT markers on Multiome
ADT-CD10
ADT-CD21
ADT-IgM
ADT-IgD
ADT-CD1c
ADT-CD27
ADT-CD45RB
ADT-CD11c
Imputed ADT Expression
Low
High
WNN_UMAP2
WNN_UMAP1
f
Top DEGs for labeled B cells in CITE-seq dataset (Reference)
TS
Naive
Naive_MZP
MZB
IgM.only
DN1
Memory
aNAV_DN2
aNAV_DN2
Memory
DN1
IgM.only
MZB
Naive_MZP
Naive
TS
Gene Expression
Cells (%)
0
25
50
75
100
g
Validation of labeled B cell clusters in Multiome by gene signatures from CITE-seq
PB
DN2
aNAV
Memory
IgM.only
MZB
Naive_MZP
Naive
TS_Naive
TS
Gene Expression
Cells (%)
h
Validation of MZB and memory cells in Multiome by published gene signatures
ZNF804A
ESR2
ADAMTS6
GPM6A
RGS7
COCH
SSBP3
LCP2
TOX
PARM1
ILDR1
HOPX
UBE2QL1
MIAT
TRERF1
EPHA4
HTR3A
SH3RF1
APOBEC3B
RASSF6
MZB
Memory
Gene Expression
Cells (%)
10
20
30
40
50

Supplementary Figure 4. B cell labeling and B cell identity validation in Multiome and CITE-seq datasets.

(a) Gene expression of major B cell markers overlaid onto the RNA UMAP space in CITE-seq dataset.

(b) Expression of major B cell surface protein markers overlaid onto the RNA UMAP space in CITE-seq dataset.
(c) Bar plot of mutation frequencies for labeled B cell subsets in CITE-seq dataset.

(d) Nuclear gene expression of major B cell markers overlaid onto the WNN UMAP space in Multiome dataset.

(e) Imputed expression of major B cell surface protein markers on Multiome after ADT data transfer from CITE-seq dataset onto the Multiome dataset, visualized onto the WNN UMAP space.

(f) Dot plot of z-score normalized mean expression of selected genes from top DEGs of labeled B cell clusters in CITE-seq dataset. Dot size represents the percentage of cells expressing the gene, the color code represents the average gene expression per subset.

(g) Dot plot of z-score normalized mean gene expression of DEGs from (**f**) in labeled B cell populations in Multiome dataset. Labels on top of the plot indicate the clusters of origin from CITE-seq. B cell populations from Multiome dataset are indicated on the left-end side of the plot. Consistency in gene expression was observed for equally labeled clusters between Multiome and CITE-seq datasets and was used for validation of B cell identity in Multiome. Dot size represents the percentage of cells expressing the gene, the color code represents the average gene expression per subset.

(h) Z-score normalized expression in labeled B cells from Multiome of previously identified markers for MZB and memory cells, used as further validation of assigned B cell identities within the dataset. Dot size represents the percentage of cells expressing the gene, the color code represents the average gene expression per subset.

TS: transitional; T1: transitional 1; T2M^{hi}: transitional IgM^{high} T2 cell; T2M^{lo}: transitional IgM^{low} T2 cell; MZP: marginal zone precursor; MZB: marginal zone B cell; DN1: double negative 1; DN2: double negative 2; IgM.only: IgM-only; aNAV: activated naïve; PB: plasmablast; ADT: antibody-derived tag.

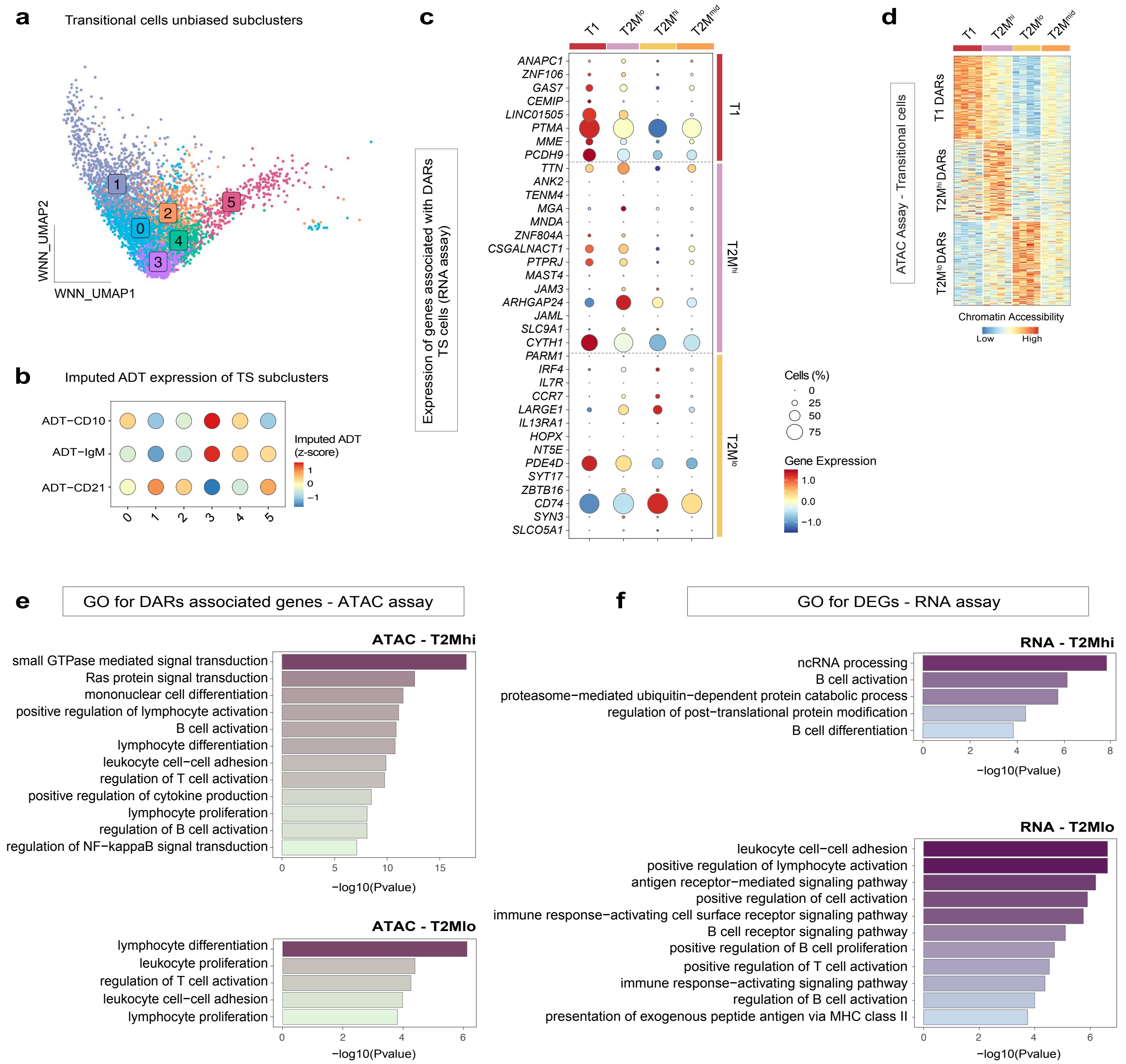
a
Transitional cells unbiased subclusters
WNN_UMAP1
WNN_UMAP2
b
Imputed ADT expression of TS subclusters
ADT−CD10
ADT−IgM
ADT−CD21
Imputed ADT (z-score)
c
Expression of genes associated with DARs TS cells (RNA assay)
T1
T2Mlo
T2Mhi
T2Mmid
ANAPC1
ZNF106
GAS7
CEMIP
LINC01505
PTMA
MME
PCDH9
TTN
ANK2
TENM4
MGA
MNDA
ZNF804A
CSGALNACT1
PTPRJ
MAST4
JAM3
ARHGAP24
JAML
SLC9A1
CYTH1
PARM1
IRF4
IL7R
CCR7
LARGE1
IL13RA1
HOPX
NT5E
PDE4D
SYT17
ZBTB16
CD74
SYN3
SLCO5A1
Cells (%)
Gene Expression
d
ATAC Assay - Transitional cells
T1 DARs
T2Mhi DARs
T2Mlo DARs
Chromatin Accessibility
Low
High
e
GO for DARs associated genes - ATAC assay
ATAC - T2Mhi
small GTPase mediated signal transduction
Ras protein signal transduction
mononuclear cell differentiation
positive regulation of lymphocyte activation
B cell activation
lymphocyte differentiation
leukocyte cell−cell adhesion
regulation of T cell activation
positive regulation of cytokine production
lymphocyte proliferation
regulation of B cell activation
regulation of NF−kappaB signal transduction
−log10(Pvalue)
ATAC - T2Mlo
lymphocyte differentiation
leukocyte proliferation
regulation of T cell activation
leukocyte cell−cell adhesion
lymphocyte proliferation
−log10(Pvalue)
f
GO for DEGs - RNA assay
RNA - T2Mhi
ncRNA processing
B cell activation
proteasome−mediated ubiquitin−dependent protein catabolic process
regulation of post−translational protein modification
B cell differentiation
−log10(Pvalue)
RNA - T2Mlo
leukocyte cell−cell adhesion
positive regulation of lymphocyte activation
antigen receptor−mediated signaling pathway
positive regulation of cell activation
immune response−activating cell surface receptor signaling pathway
B cell receptor signaling pathway
positive regulation of B cell proliferation
positive regulation of T cell activation
immune response−activating signaling pathway
regulation of B cell activation
presentation of exogenous peptide antigen via MHC class II
−log10(Pvalue)

Supplementary Figure 5. Investigation of transitional B cells in Multiome dataset.

(a) Unbiased RNA subclusters for transitional B cell subset from Multiome projected onto the WNN UMAP space.

(b) Dot plot of z-score normalized mean expression of imputed cell surface markers CD10, CD21 and IgM for unbiased transitional B cell subclusters in Multiome dataset.

(c) Dot plot of z-score normalized mean expression of genes indicated in **Fig. 3j**, associated with peaks that showed differential chromatin accessibility among transitional cells, represented in T1, $T2M^{hi}$, $T2M^{lo}$ and $T2M^{mid}$ cells. Dot size represents the percentage of cells expressing the gene, the color code represents the average gene expression per subset.

(d) Pseudo-bulk heatmap of z-score normalized chromatin accessibility of top 150 DARs for T1, $T2M^{hi}$ and $T2M^{lo}$ cells in comparison to other transitional cells compared in T1, $T2M^{hi}$, $T2M^{lo}$ and $T2M^{mid}$ cells. Columns are separated in biological replicates.

(e) Bar charts of top enriched pathways from GO analysis of genes associated with statistically significant DARs in $T2M^{hi}$ **(top)** or $T2M^{lo}$ **(bottom)** cells in comparison to other transitional cells. Genes used in the GO analysis were selected based on their proximity (distance ≤ 2000 bp) to DARs located at the UTRs or coding regions of protein-coding genes. Statistical analysis was performed with one-sided Fisher's exact test with Benjamini-Hochberg test for multiple comparisons. Bar length represents the statistical significance of enriched pathways, expressed as -Log_{10}(p-value).

(f) Bar chart of top enriched pathways from GO analysis of genes with increased transcript abundance (adjusted p-value ≤ 0.05) in $T2M^{hi}$ **(top)** or $T2M^{lo}$ **(bottom)** cells in comparison to other transitional cells. Statistical analysis was performed with one-sided Fisher's exact test with Benjamini-Hochberg test for multiple comparisons. Bar length represents the statistical significance of enriched pathways, expressed as -Log_{10}(p-value).

TS: transitional; T1: transitional 1; $T2M^{hi}$: transitional IgM^{high} T2 cell; $T2M^{lo}$: transitional IgM^{low} T2 cell; $T2M^{mid}$: transitional IgM^{mid} T2 cell; ADT: antibody-derived tag; GO: gene ontology; DAR: differentially accessible region; DEG: differentially expressed gene.

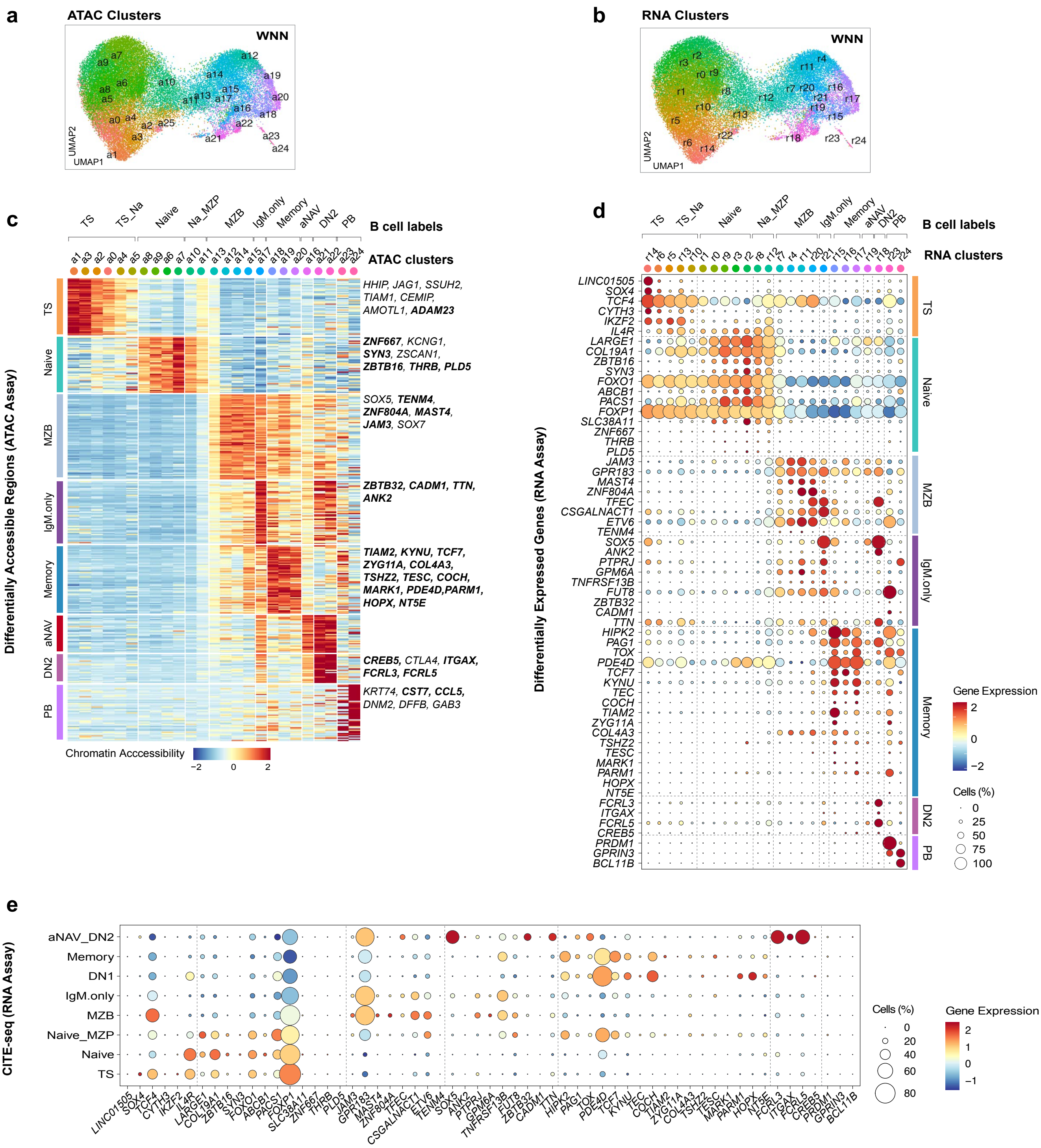
a
ATAC Clusters
WNN
UMAP1
UMAP2
b
RNA Clusters
WNN
UMAP1
UMAP2
c
B cell labels
ATAC clusters
TS
TS_Na
Naive
Na_MZP
MZB
IgM.only
Memory
aNAV
DN2
PB
Differentially Accessible Regions (ATAC Assay)
HHIP, JAG1, SSUH2, TIAM1, CEMIP, AMOTL1, ADAM23
ZNF667, KCNG1, SYN3, ZSCAN1, ZBTB16, THRB, PLD5
SOX5, TENM4, ZNF804A, MAST4, JAM3, SOX7
ZBTB32, CADM1, TTN, ANK2
TIAM2, KYNU, TCF7, ZYG11A, COL4A3, TSHZ2, TESC, COCH, MARK1, PDE4D,PARM1, HOPX, NT5E
CREB5, CTLA4, ITGAX, FCRL3, FCRL5
KRT74, CST7, CCL5, DNM2, DFFB, GAB3
Chromatin Acccessibility
d
B cell labels
RNA clusters
Differentially Expressed Genes (RNA Assay)
Gene Expression
Cells (%)
e
CITE-seq (RNA Assay)
aNAV_DN2
Memory
DN1
IgM.only
MZB
Naive_MZP
Naive
TS
Cells (%)
Gene Expression

Supplementary Figure 6. Investigation of features of B cells subsets in Multiome dataset.

(a, b) Unsupervised ATAC **(a)** and RNA **(b)** clusters on B cells from Multiome represented onto the WNN UMAP space.

(c) Pseudo-bulk heatmap of z-score normalized chromatin accessibility for top 50 DARs of major B cell subpopulations, as highlighted in **Fig. 5a**. DARs are represented along unbiased ATAC clusters, grouped based on common B cell labeling. Key genes associated with top DARs located at TSSs are listed on the right-hand side of the heatmap.

(d) Dot plot of z-score normalized mean expression of genes indicated in **Fig. 5b,** associated with top DARs of B cell subsets, along with additional genes from **(a)** that were not among top DEGs for related B cell subsets, hence not indicated in **Fig.5b**. Gene expression is represented along unsupervised RNA clusters, grouped based on B cell labeling. Dot size represents the percentage of cells expressing the gene, the color code represents the average gene expression per subset.

(e) Validation of top DEGs from Multiome B cell clusters by investigation of their expression in CITE-seq dataset. B cell populations from CITE-seq dataset are indicated on the left-end side. Dot size represents the percentage of cells expressing the gene, the color code represents the average gene expression per subset.

TS: transitional; MZP: marginal zone precursor; MZB: marginal zone B cell; IgM.only: IgM-only; DN1: double negative 1; DN2: double negative 2; aNAV: activated naïve; PB: plasmablast.

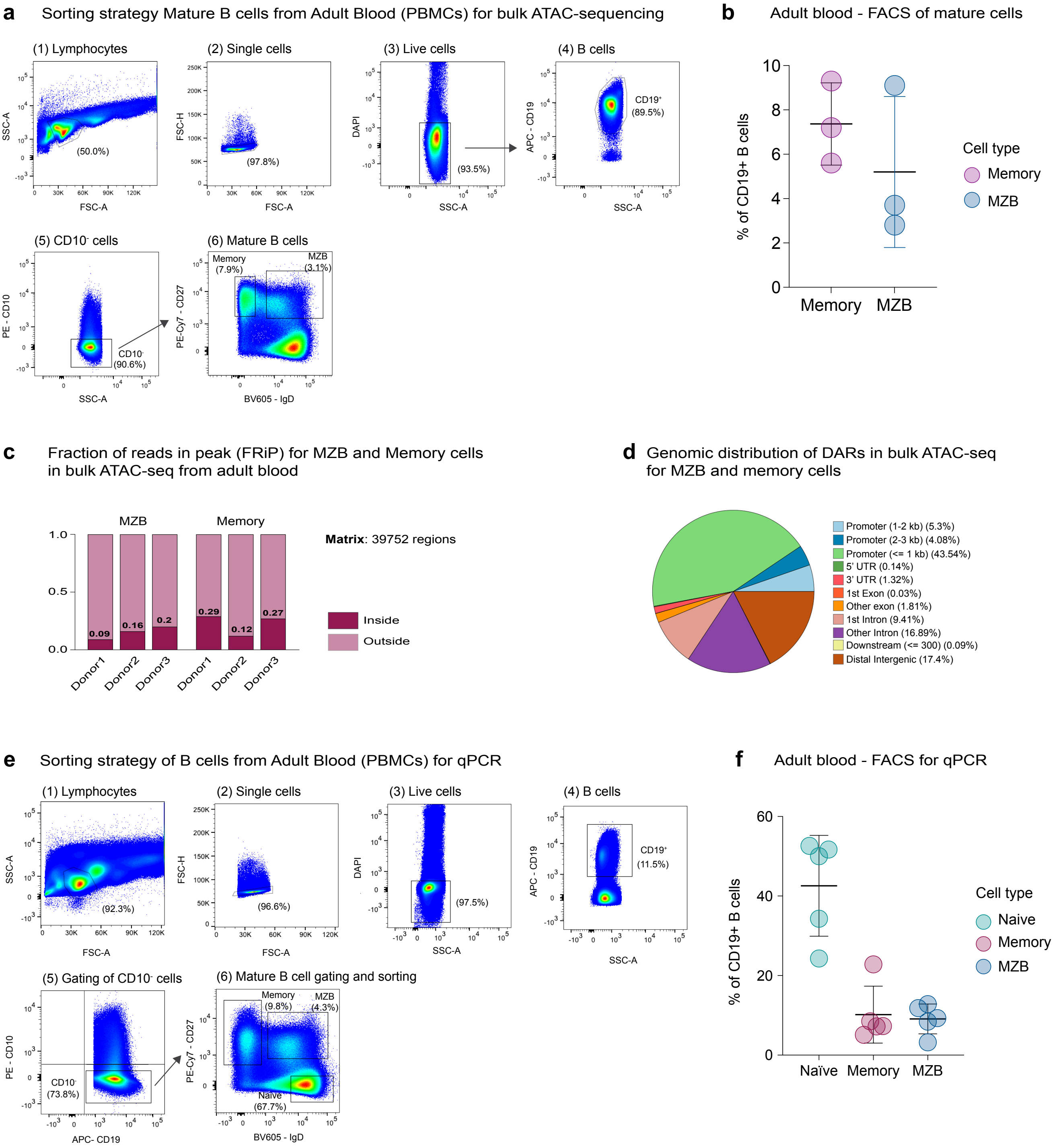

a
Sorting strategy Mature B cells from Adult Blood (PBMCs) for bulk ATAC-sequencing
(1) Lymphocytes
(50.0%)
SSC-A
FSC-A
(2) Single cells
(97.8%)
FSC-H
FSC-A
(3) Live cells
(93.5%)
DAPI
SSC-A
(4) B cells
CD19⁺
(89.5%)
APC - CD19
SSC-A
(5) CD10⁻ cells
CD10⁻
(90.6%)
PE - CD10
SSC-A
(6) Mature B cells
Memory
(7.9%)
MZB
(3.1%)
PE-Cy7 - CD27
BV605 - IgD
b
Adult blood - FACS of mature cells
% of CD19+ B cells
Memory
MZB
Cell type
Memory
MZB
c
Fraction of reads in peak (FRiP) for MZB and Memory cells in bulk ATAC-seq from adult blood
MZB
Memory
0.09
0.16
0.2
0.29
0.12
0.27
Donor1
Donor2
Donor3
Donor1
Donor2
Donor3
Matrix: 39752 regions
Inside
Outside
d
Genomic distribution of DARs in bulk ATAC-seq for MZB and memory cells
Promoter (1-2 kb) (5.3%)
Promoter (2-3 kb) (4.08%)
Promoter (<= 1 kb) (43.54%)
5' UTR (0.14%)
3' UTR (1.32%)
1st Exon (0.03%)
Other exon (1.81%)
1st Intron (9.41%)
Other Intron (16.89%)
Downstream (<= 300) (0.09%)
Distal Intergenic (17.4%)
e
Sorting strategy of B cells from Adult Blood (PBMCs) for qPCR
(1) Lymphocytes
(92.3%)
SSC-A
FSC-A
(2) Single cells
(96.6%)
FSC-H
FSC-A
(3) Live cells
(97.5%)
DAPI
SSC-A
(4) B cells
CD19⁺
(11.5%)
APC - CD19
SSC-A
(5) Gating of CD10⁻ cells
CD10⁻
(73.8%)
PE - CD10
APC- CD19
(6) Mature B cell gating and sorting
Memory
(9.8%)
MZB
(4.3%)
Naive
(67.7%)
PE-Cy7 - CD27
BV605 - IgD
f
Adult blood - FACS for qPCR
% of CD19+ B cells
Naïve
Memory
MZB
Cell type
Naive
Memory
MZB

Supplementary Figure 7. Bulk ATAC-seq and RT-qPCR analyses of B cells from adult PBMCs.

(a) Representative B cell sorting strategy for mature B cell subsets from adult PBMCs in bulk ATAC-seq experiments (n=3 biological replicates). Single live cells were selected based on negative DAPI staining. B cells were selected based on $CD19^+$ expression. $CD10^-$ cells were gated and separated based on expression of IgD and CD27. Memory cells were gated as $CD27^+IgD^-$ cells and sorted. MZB were gated as $CD27^+IgD^+$ cells and sorted. Percentages of cells at the end of sorting from a representative biological replicate are indicated. Source data is provided as a Source Data file.

(b) Dot plot of final percentages of memory and MZB sorted $CD19^+$ B cells from adult human PBMCs in bulk ATAC-seq experiments (n=3). Error bars represent mean ± SD.

(c) Matrix size and FriP for each of the individual bulk ATAC-seq libraries for MZB and memory cells from adult blood.

(d) Proportions of DARs in adult blood MZB and memory cells mapping to distinct genomic regions in bulk ATAC-seq.

(e) Representative B cell sorting strategy for mature B cell subsets from adult PBMCs in bulk RT-qPCR experiments (n=5 biological replicates). Single live cells were selected based on negative DAPI staining. B cells were selected based on $CD19^+$ expression. $CD10^-$ cells were gated and separated based on expression of IgD and CD27. Memory cells were gated as $CD27^+IgD^-$ cells and sorted. MZB were gated as $CD27^+IgD^+$ cells and sorted. Naïve cells were gated as $CD27^-IgD^+$ cells and sorted. Percentages of cells at the end of sorting from a representative biological replicate are indicated. Source data is provided as a Source Data file.

(f) Dot plot of final percentages of naïve, memory and MZB sorted $CD19^+$ B cells from adult human PBMCs in bulk RT-pPCR experiments. Error bars are mean ± SD.

PBMC: peripheral blood mononuclear cell; MZB: marginal zone B cell; FRiP: fraction of reads in peaks; DAR: differentially accessible region; FACS: fluorescent-activated cell sorting; UTR: untranslated region; RT-qPCR: reverse transcription quantitative polymerase chain reaction.

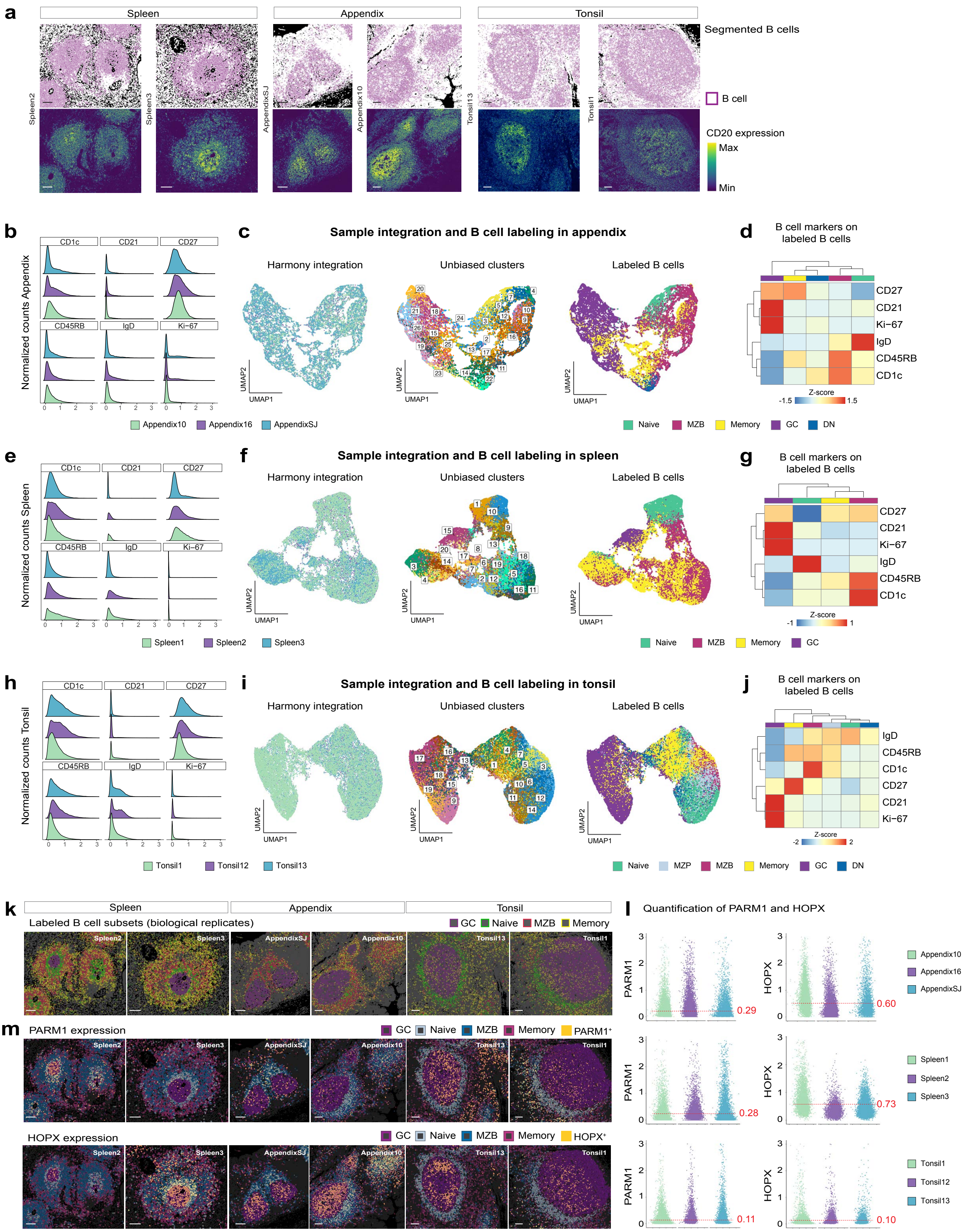

a
Spleen
Appendix
Tonsil
Segmented B cells
B cell
CD20 expression
Max
Min
Spleen2
Spleen3
AppendixSJ
Appendix10
Tonsil13
Tonsil1
b
Normalized counts Appendix
CD1c
CD21
CD27
CD45RB
IgD
Ki−67
Appendix10
Appendix16
AppendixSJ
c
Sample integration and B cell labeling in appendix
Harmony integration
Unbiased clusters
Labeled B cells
UMAP1
UMAP2
Naive
MZB
Memory
GC
DN
d
B cell markers on labeled B cells
Z-score
e
Normalized counts Spleen
Spleen1
Spleen2
Spleen3
f
Sample integration and B cell labeling in spleen
g
h
Normalized counts Tonsil
Tonsil1
Tonsil12
Tonsil13
i
Sample integration and B cell labeling in tonsil
j
MZP
k
Labeled B cell subsets (biological replicates)
l
Quantification of PARM1 and HOPX
PARM1
HOPX
m
PARM1 expression
PARM1⁺
HOPX expression
HOPX⁺

Supplementary Figure 8. Identification of B cell subsets and investigation of PARM1 and HOPX expression in lymphoid tissues by Imaging Mass Cytometry.

(a) B cell segmentation in representative ROIs from biological replicates for appendix, spleen and tonsil (n=3), along with single-channel images displaying CD20 protein expression (viridis color scale) in the same regions. Overlap between labeled B cells and CD20 expression was observed in all tissues.

(b, e, h) Density plots showing the distribution of normalized counts of major protein markers used for B cell labeling across tissue replicates for appendix, spleen and tonsil.

(c, f, i) Harmony algorithm was used for batch effect correction and data integration on segmented B cells from tissue replicates of appendix, spleen and tonsil. Unbiased clusters were generated using the Shared Nearest Neighbor Graph method and B cell identity was assigned based on expression of major B cell protein markers in each cluster. Clusters with no clear marker expression were excluded from subsequent analysis.

(d, g, j) Clustered heatmaps of z-score normalized mean expression of major B cell protein markers in labeled B cells in lymphoid tissues.

(k) Representative ROIs from biological replicates showing distribution of computationally labeled B cell populations in the secondary lymphoid organs included in the analysis.

(l) Scatter plots of *PARM1* and *HOPX* normalized counts across tissue replicates for appendix, spleen and tonsil. The third quartile of count distributions is indicated with a horizontal dotted red line and exact values are indicated on the right-end side.

(m) *PARM1* and *HOPX*-positive cells quantified in **Fig.9d,e** visualized in biological replicates of secondary lymphoid organs included in the study.

Scale bars represent 100 μm.

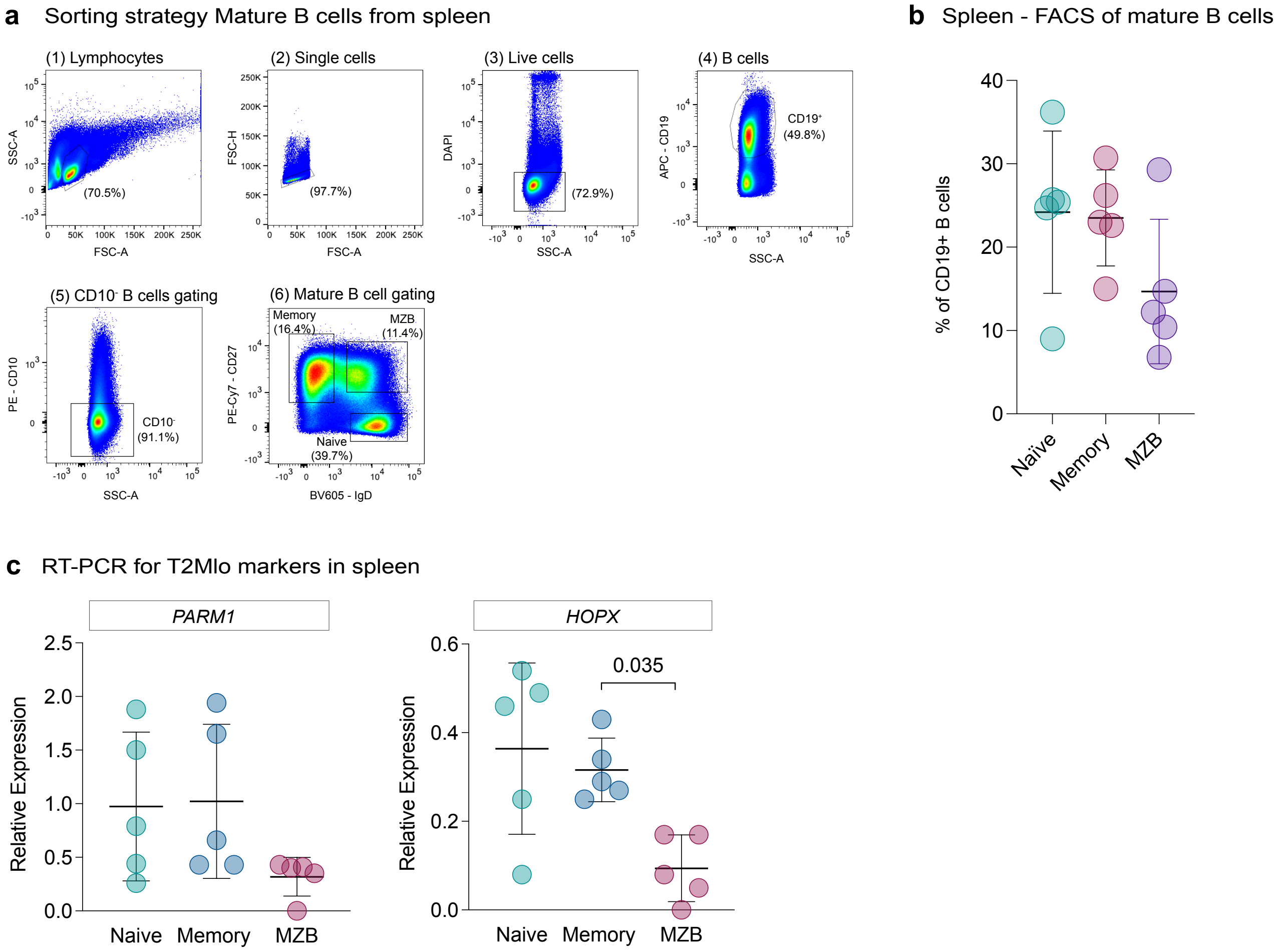
a Sorting strategy Mature B cells from spleen
(1) Lymphocytes
(70.5%)
SSC-A
FSC-A
(2) Single cells
(97.7%)
FSC-H
FSC-A
(3) Live cells
(72.9%)
DAPI
SSC-A
(4) B cells
CD19+
(49.8%)
APC - CD19
SSC-A
(5) CD10- B cells gating
CD10-
(91.1%)
PE - CD10
SSC-A
(6) Mature B cell gating
Memory
(16.4%)
MZB
(11.4%)
Naive
(39.7%)
PE-Cy7 - CD27
BV605 - IgD
b Spleen - FACS of mature B cells
% of CD19+ B cells
Naïve
Memory
MZB
c RT-PCR for T2Mlo markers in spleen
PARM1
Relative Expression
Naive
Memory
MZB
HOPX
0.035
Relative Expression
Naive
Memory
MZB

Supplementary Figure 9. Investigation of *PARM1* and *HOPX* expression in sorted B cells from human spleen by bulk RT-qPCR.

(a) Representative sorting strategy of B cells from human spleen cell suspensions (n=5 biological replicates). Single live cells were selected based on negative DAPI staining. B cells were selected based on $CD19^+$ expression. $CD10^-$ cells were gated and separated based on expression of IgD and CD27. Memory cells were gated as $CD27^+IgD^-$ cells and sorted. MZB were gated as $CD27^+IgD^+$ cells and sorted. Naïve cells were gated as $CD27^-IgD^+$ cells and sorted. Percentages of cells at the end of sorting from a representative biological replicate are indicated. Source data is provided as a Source Data file.

(b) Dot plot of final percentages of naïve, memory and MZB sorted $CD19^+$ B cells from adult spleen cell suspensions in bulk RT-qPCR experiments (n=5). Error bars represent mean ± SD.

(c) RT-qPCR analysis of $T2M^{lo}$ markers *PARM1* and *HOPX* in sorted naïve, MZB and memory B cells from human spleen cell suspensions (n=5). Colored dots represent mean mRNA expression of mRNA targets over endogenous controls (18S) for independent biological replicates. Mean values across biological replicates are represented as horizontal bars. For *PARM1,* statistical comparison was performed using the Friedman test with Dunn's correction for multiple comparisons. For *HOPX,* statistical comparison was made using one-way ANOVA with post-hoc Tukey's test for multiple comparisons. Only statistically significant values are represented as exact p-values. Error bars represent mean ± SD. All tests were two-sided.

FACS: fluorescent activated cell sorting; MZB: marginal zone B cell; RT-qPCR: reverse transcription quantitative polymerase chain reaction; $T2M^{lo}$: transitional IgM^{low} T2 cell.

Supplementary Table 1. Materials used in this study.

**Antibodies for B cell sorting for bulk ATAC-seq and H3K4me3 - TS cells**					
**Target**	**Fluorophore**	**Clone**	**Cat. Number**	**Supplier**	**Dilution**
CD19	APC	HIB19	20-0199-T100	TONDO Bioscience	1 in 50
CD10	PE	HI10a	312203	BioLegend	1 in 200
IgM	BV711	MHM-88	314539	BioLegend	1 in 50
**Antibodies for B cell sorting for bulk ATAC-seq and H3K4me3 - Mature cells**					
**Target**	**Fluorophore**	**Clone**	**Cat. Number**	**Supplier**	**Dilution**
CD19	APC	HIB19	20-0199-T100	TONDO Bioscience	1 in 50
CD10	PE	HI10a	312203	BioLegend	1 in 200
IgD	BV605	IA6-2	563313	BioLegend	1 in 100
CD27	PE-Cy7	M-T271	356411	BioLegend	1 in 50
**Antibodies for CUT&RUN**					
**Target**	**Cat. Number**	**Supplier**	**Dilution**		
H3K4me3	Ab8580	Abcam	1 µg/sample		
IgG isotype control	12-370	Millipore	1 µg/sample		
**Antibodies for B cell sorting for bulk qPCR - Blood and Spleen Samples**					
**Target**	**Fluorophore**	**Clone**	**Cat. Number**	**Supplier**	**Dilution**
CD19	APC	HIB19	20-0199-T100	TONDO Bioscience	1 in 50
CD10	PE	HI10a	312203	BioLegend	1 in 200
IgD	BV605	IA6-2	563313	BioLegend	1 in 100
CD27	PE-Cy7	M-T271	356411	BioLegend	1 in 50
**Antibodies for B cell sorting for single-cell Multiome**					
**Target**	**Fluorophore**	**Clone**	**Cat. Number**	**Supplier**	**Dilution**
CD19	PE-Dazzle	SJ25C1	363031	BioLegend	1 in 50
**Antibodies for B cell sorting for single-cell CITE-seq**					
**Target**	**Fluorophore**	**Clone**	**Cat. Number**	**Supplier**	**Dilution**
CD19	PE-Dazzle	HIB19	302251	BioLegend	1 in 100
CD19	PE-Dazzle	SJ25C1	363031	BioLegend	1 in 100
**ADT antibodies for CITE-seq experiments**					
**Target**	**Antibody**	**Clone**	**Barcode Sequence**	**Cat. Number**	**Dilution**
CD1C	TotalSeq-C0160	L161	GAGCTACTTCACTCG	331547	0.25 µg/sample
CD11C	TotalSeq-C0053	S-HCL-3	TACGCCTATAACTTG	371521	0.25 µg/sample
CD45RB	TotalSeq-C0844	MEM-55	AGATGGGACTCACCA	310211	0.25 µg/sample
CD10	TotalSeq-C0062	HI10a	CAGCCATTCATTAGG	312233	0.25 µg/sample
CD21	TotalSeq-C0181	Bu32	AACCTAGTAGTTCGG	354923	0.25 µg/sample
CD27	TotalSeq-C0154	O323	GCACTCCTGCATGTA	302853	0.25 µg/sample
IgM	TotalSeq-C0136	MHM-88	TAGCGAGCCCGTATA	314547	0.25 µg/sample
IgD	TotalSeq-C0384	IA62	CAGTCTCCGTAGAGT	348245	0.25 µg/sample
**Oligonucleotide probes for RT-qPCR - TaqMan gene Expression Assay**					
**Target**	**TaqMan Assay ID**	**Supplier**	**Chr location (GRCh38)**		
PARM1	HS00209876_m1	ThermoFisher	Chr.4: 74933075 - 75050115		
HOPX	HS00261238_m1	ThermoFisher	Chr.4: 56647988 - 56681866		
MAST4	HS00389519_m1	ThermoFisher	Chr.5: 66596348 - 67169595		
ZNF804A	HS00290118_S1	ThermoFisher	Chr.2: 184598366 - 184939487		
Eucaryotic 18S rRNA (control)	Hs99999901_s1	Fisher Scientific	NA		
**Antibodies for Imaging Mass Cytometry**					
**Metal**	**Marker**	**Clone**	**Cat. Number**	**Dilution**	
144Nd	FITC (for HOPX_RNA probe)	FIT-22	3144006C	1 in 100	
146Nd	CD21	EP3093	ab271855	1 in 100	
148Nd	CD45RB	310202	MEM-55	1 in 500	
154Sm	IgD	EPR6146	ab236778	1 in 300	
158Gd	E-Cadherin	24E10	3158029D	1 in 3000	
161Dy	CD20	SP32	ab236434	1 in 300	
164Dy	CD1c	OTI2F4	NBP2-46123	1 in 500	
166Er	BIOTIN (for PARM1_RNA probe)	409002	1D4-C5	1 in 150	
168Er	Ki-67	B56	3168022D	1 in 400	
171Yb	CD27	EPR8569	3171024D	1 in 300	

Supplementary Table 2. Definitions and notes related to human B cell clusters and subsets referred to in the manuscript.

Subset name	Abbreviation used in the manuscript	Biological relevance
Transitional B cells	TS	Refers to the total population of transitional B cells including the earliest bone marrow emigrants, T1, that express high levels of CD10, CD24 and CD38 and more mature T2 cells that have reduced expression of CD10, CD24 and CD38 compared to T1[1, 2, 3].
Type 1 transitional B cells	T1	Earliest bone marrow emigrants identifed in adult blood. Express highest level of CD10, CD24 and CD38 in the total B cell population[1, 4].
Type 2 transitional B cells	T2	Immature B cells that express CD10, CD24 and CD38 but at lower levels than T1 B cells[1, 2, 4].
T2 cells expressing the highest level of IgM	$T2M^{hi}$	T2 cells with highest expression of IgM protein. These have been proposed to be precursors of marginal zone B cells in an earlier study. They express a4b7 and have the potential to home to the gut[5].
T2 cells expressing the lowest level of IgM	$T2M^{lo}$	T2 cells with the lowest level of IgM expression. It has been proposed that they may develop into recirculating naïve B cells in a review but this is explored for the first time in this manuscript[5, 6].
Naïve B cells	Naïve	Mature B cells with the phenotype CD27-IgM+IgD+. They are considered to be the population that circulates between tissues forming the follicular mantle. They are often referred to as follicular B cells in the study of mouse B cells[7, 8, 9].
Activated Naïve cells	aNAV	aNAV are considered to be derived from naïve B cells by innate signals such as IFNg and TLR7 ligation[10].
Double negative 2	DN2	Derivation of double negative type 2 B cells from activated naive B cells has been described in other studies. They are 'double negative' because they lack both the memory associated marker CD27 and naïve B cell associated immunoglobulin IgD. They can be positively identified by their expression of CD11c encoding gene ITGAX.[10]
Marginal zone precursor cells	MZP	MZP were originally discriminated from naïve B cells by their high expression of IgM and also expression of the gycosylated form of CD45RB that is otherwise associated with memory B cells. They were shown to be precursors of MZB by trajectory analysis of cells in blood and also by invitro demonstration of a requirement of NOTCH2 ligation by DDL1 for maturation[5, 11, 12].
Marginal zone B cells	MZB	B cells expressing CD27, IgM, IgD and CD1c. They are derived in human from transitional B cells expressing high levels of surface IgM. They are associated with a developmental pathway from MZP involving NOTCH2/ delta ligand like 1 interactions[11, 12, 13].
Canonical class switched memory cells	Memory	Clusters designated memory B cells here are associated with expression of IgA and IgG, though some IgM expressing cells are also present. They are the population considered to be derived from germinal centres in lymphoid tissues following cognate interaction with T cells[14, 15, 16].
B cells expressing CD27 and IgM but no other immunoglobulin isotype.	IgM-only	This interesting population tends to cluster separately from class-switched memory and MZB, though has been linked to both in the literature. Strongly associated with mucosal IgM responses and IgM plasma cell production[17, 18, 19].
Intermediate/ mid and unknown populations		In general, UMAP illustrations of B cell gene expression or chromatin state tend to include clear clusters though often poorly separated and with unclear boundaries[5, 20, 21, 22]. In this study we excluded clusters we considered to be mixed, partially differentiated or in other circumstances where we were unsure about designation to avoid mistakes. The $T2M^{mid}$ population, for example, was interrogated but found to have no markers that aligned it to T1, T2Mhi or T2Mlo subsets (Supplementary Fig. 5). It was therefore not included in the deeper analysis.

References

1. Bemark M. Translating transitions - how to decipher peripheral human B cell development. *J Biomed Res* **29**, 264-284 (2015).

2. Palanichamy A*, et al.* Novel human transitional B cell populations revealed by B cell depletion therapy. *J Immunol* **182**, 5982-5993 (2009).

3. Wirths S, Lanzavecchia A. ABCB1 transporter discriminates human resting naive B cells from cycling transitional and memory B cells. *Eur J Immunol* **35**, 3433-3441 (2005).

4. Suryani S*, et al.* Differential expression of CD21 identifies developmentally and functionally distinct subsets of human transitional B cells. *Blood* **115**, 519-529 (2010).

5. Tull TJ*, et al.* Human marginal zone B cell development from early T2 progenitors. *J Exp Med* **218**, (2021).

6. Morgan D, Tergaonkar V. Unraveling B cell trajectories at single cell resolution. *Trends Immunol* **43**, 210-229 (2022).

7. Chen JW*, et al.* Positive and negative selection shape the human naive B cell repertoire. *J Clin Invest* **132**, (2022).

8. Weisel NM*, et al.* Surface phenotypes of naive and memory B cells in mouse and human tissues. *Nat Immunol* **23**, 135-145 (2022).

9. Watson SR, Bradley LM. The recirculation of naive and memory lymphocytes. *Cell Adhes Commun* **6**, 105-110 (1998).

10. Jenks SA*, et al.* Distinct Effector B Cells Induced by Unregulated Toll-like Receptor 7 Contribute to Pathogenic Responses in Systemic Lupus Erythematosus. *Immunity* **49**, 725-739 e726 (2018).

11. Koethe S*, et al.* Pivotal advance: CD45RB glycosylation is specifically regulated during human peripheral B cell differentiation. *J Leukoc Biol* **90**, 5-19 (2011).

12. Descatoire M*, et al.* Identification of a human splenic marginal zone B cell precursor with NOTCH2-dependent differentiation properties. *J Exp Med* **211**, 987-1000 (2014).

13. Weill JC, Weller S, Reynaud CA. Human marginal zone B cells. *Annu Rev Immunol* **27**, 267-285 (2009).

14. Ehrhardt GR, Hijikata A, Kitamura H, Ohara O, Wang JY, Cooper MD. Discriminating gene expression profiles of memory B cell subpopulations. *J Exp Med* **205**, 1807-1817 (2008).

15. Shlomchik MJ, Weisel F. Germinal center selection and the development of memory B and plasma cells. *Immunol Rev* **247**, 52-63 (2012).

16. Weisel NM*, et al.* Comprehensive analyses of B-cell compartments across the human body reveal novel subsets and a gut-resident memory phenotype. *Blood* **136**, 2774-2785 (2020).

17. Magri G*, et al.* Human Secretory IgM Emerges from Plasma Cells Clonally Related to Gut Memory B Cells and Targets Highly Diverse Commensals. *Immunity* **47**, 118-134.e118 (2017).

18. Zhao Y*, et al.* Spatiotemporal segregation of human marginal zone and memory B cell populations in lymphoid tissue. *Nat Commun* **9**, 3857 (2018).

19. Klein U, Kuppers R, Rajewsky K. Evidence for a large compartment of IgM-expressing memory B cells in humans. *Blood* **89**, 1288-1298 (1997).

20. Glass DR*, et al.* An Integrated Multi-omic Single-Cell Atlas of Human B Cell Identity. *Immunity* **53**, 217-232 e215 (2020).

21. Siu JHY*, et al.* Two subsets of human marginal zone B cells resolved by global analysis of lymphoid tissues and blood. *Sci Immunol* **7**, eabm9060 (2022).

22. Yang X*, et al.* Single-cell analysis reveals multi-faceted features of B cell development, together with age-associated B cell subpopulations. *Commun Biol* **9**, 240 (2026).
