# Supplementary material for: Epigenetic signatures mark early peripheral human B lineage bifurcation and differential transcriptional profiles in mature populations
Source: Nat Commun. 2026 Jul 14;17:8628. doi: 10.1038/s41467-026-75364-3 (PMC13487194; doi:10.1038/s41467-026-75364-3)
Supplement: Supplementary file 4 [file 41467_2026_75364_MOESM2_ESM.pdf]

Description of Supplementary Files

Supplementary Data 1 – Bulk ATAC-seq differential analysis on human adult blood for TS cell subsets.
Statistically significant DARs.

Supplementary Data 2 – Bulk H3K4me3 CUT&RUN on human adult blood for $T2M^{hi}$ and $T2M^{lo}$ cells.
Statistically significant differentially enriched regions.

Supplementary Data 3 – Bulk ATAC-seq differential analysis on human cord blood between$T2M^{hi}$ and $T2M^{lo}$ cells.
Statistically significant DARs.

Supplementary Data 4 – RNA assay from CITE-seq dataset.
Statistically significant DEGs for labeled B cell subsets in comparison to other B cells.

Supplementary Data 5 – ATAC assay from Multiome dataset for transitional B cell subset.
Statistically significant DARs for labeled transitional B cells compared to other transitional cells.

Supplementary Data 6 – RNA assay from Multiome dataset for transitional B cell subset.
Statistically significant DEGs for labeled transitional B cells compared to other transitional cells.

Supplementary Data 7 – Gene Ontology analysis for DEGs and DARs in transitional cells.

Supplementary Data 8 – Monocle and scMEGA trajectory analyses.
Statistically significant DEGs and TFs in the trajectory analyses performed with Monocle and scMEGA along identified trajectories.

Supplementary Data 9 – RNA assay from Multiome dataset for all B cells.
Statistically significant DEGs for labeled B cells compared to all other B cells.

Supplementary Data 10 – ATAC assay from Multiome dataset for all B cells.
Statistically significant DARs for labeled B cells compared to all other B cells.

Supplementary Data 11 – Bulk ATAC-seq differential analysis between memory and MZB cells from human adult blood.
Statistically significant DARs.

Supplementary Data 12 – GeneActivity assay from Multiome dataset for all B cells.
Statistically significant differentially accessible genes for labeled B cells compared to all other B cells.

Supplementary Data 13 – ChromVAR assay from Multiome dataset for all B cells.
TFs showing differential activity in labeled B cells compared to all other B cells, along with associated binding motifs.

Supplementary Data 14 – Metadata of Regions of Interest included in IMC study.
